# Conformational Preference of 2′-Fluoro-Substituted Acetophenone Derivatives Revealed by Through-Space ^1^H–^19^F and ^13^C–^19^F Spin–Spin Couplings

**DOI:** 10.1021/acs.joc.1c00051

**Published:** 2021-03-01

**Authors:** Chinatsu Otake, Takuya Namba, Hidetsugu Tabata, Kosho Makino, Kiriko Hirano, Tetsuta Oshitari, Hideaki Natsugari, Takenori Kusumi, Hideyo Takahashi

**Affiliations:** † Faculty of Pharmaceutical Sciences, Tokyo University of Science, 2641Yamazaki, Noda-shi, Chiba 278-8510, Japan; ‡ Faculty of Pharma Sciences, Teikyo University, 2-11-1 Kaga, Itabashi-ku, Tokyo 173-8605, Japan; § Bruker Japan K.K., 3-9 Moriya, Kanagawa-ku, Yokohama, Kanagawa 221-0022, Japan; ∥ Graduate School of Pharmaceutical Science, The University of Tokyo, 7-3-1 Hongo, Bunkyo-ku, Tokyo 113-0033, Japan; ⊥ Department of Chemistry, Tokyo Institute of Technology, Meguro-ku, Tokyo 152-8551, Japan

## Abstract

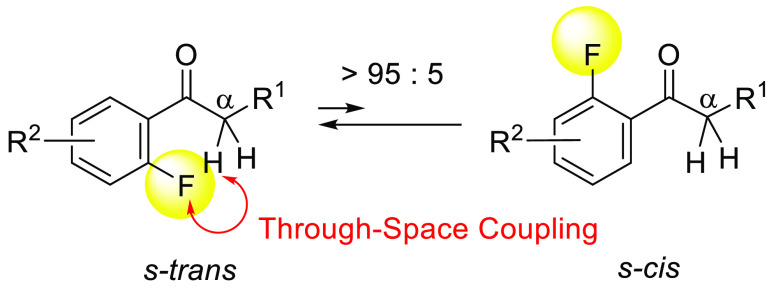

The conformational properties of 2′-fluoro-substituted acetophenone derivatives were elucidated based on H^α^–F and C^α^–F through-space spin–spin couplings (TS-couplings), which occur between two atoms constrained at a distance smaller than the sum of their van der Waals radii. This study revealed that 2′-fluoro-substituted acetophenone derivatives in solutions form exclusively *s*-*trans* conformers by analyzing their NMR spectra focused on the TS-couplings. The magnitudes of the coupling constants ^5^*J* (H^α^, F) and ^4^*J* (C^α^, F) correlate linearly with the value of the dielectric constant of the solvents. Furthermore, *s*-*trans* conformations of the two derivatives were confirmed by X-ray crystallographic analysis. These conformational preferences were consistent with the DFT calculations. The *s*-*cis* conformer, in which fluorine and oxygen atoms lie in a *syn*-periplanar mode, may be subject to strong repulsion between the two polar atoms and become unstable. The *s*-*trans* preference of the 2′-fluoro-substituted acetophenone derivatives may be utilized in drug design.

## Introduction

Fluorine, which exhibits a range of remarkable chemical, physical, and biological properties, has been recognized as a valuable element in various branches of science, including medicinal chemistry. More than 20% of known drugs contain fluorine atoms, and the immense stereoelectronic effects of fluorine on bioactive organic molecules have been extensively examined.^1^ One important tool to comprehend the structural features of fluorine compounds is NMR spectroscopy, in which fluorine (^19^F; *I* = 1/2) gives valuable hints regarding the structure and stereochemistry of compounds. One of the most peculiar of the NMR behaviors of fluorine is “through-space spin–spin coupling (TS-coupling).”^2^ TS-couplings are observed between two atoms when either has lone-pair electrons and both are constrained at a distance smaller than the sum of their van der Waals radii. Fluorine has lone-pair electrons and has been the object of numerous studies in terms of determining conformations with long-range hydrogen–fluorine (^1^H–^19^F) and carbon–fluorine (^13^C–^19^F) TS-couplings.^3^ For example, the ^1^H–^19^F TS-coupling was detected in alkylfluorobenzenes where hydrogen and fluorine were separated by five bonds.^4^^13^C–^19^F TS-coupling over five bonds was also observed in 1,4,8-trimethyl-5-fluorophenanthrene.^5^ Whenever two nuclei, such as ^19^F/^19^F, ^19^F/^1^H, and ^19^F/^13^C, are in van der Waals contact through space, regardless of how many bonds separate them, they can exchange spin information.^6^

**Figure 1:**
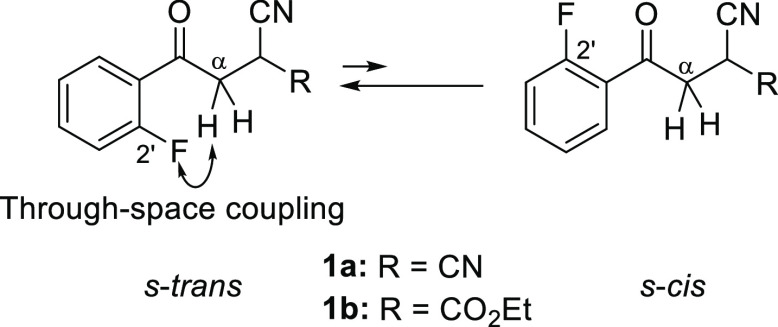
Through-space spin–spin couplings observed in **1a** and **1b**.

In the course of our studies of bioactive compounds, we synthesized acetophenone derivatives (**1a** and **1b**) (Figure 1). In the ^1^H NMR and ^13^C NMR spectra of **1a** and **1b**, we recognized significant magnitudes of TS-couplings between H^α^–F and C^α^–F. These TS-couplings may mean that two atoms (H^α^ and F/C^α^ and F) are constrained at a distance smaller than the sum of their van der Waals radii. Therefore, we deduced that compounds **1a** and **1b** prefer *s*-*trans* conformations to *cis* conformations (Figure 1), suggesting that the fluorine atoms control the conformation of the compound. Various 2′-substituted acetophenones were synthesized, and conformational studies were performed.^7^ Among the studies, Schaefer examined TS-coupling of 2-fluoro and 2,6-fluoroacetophenones based on calculations,^3d^ but that work has received relatively little attention, although the results are extremely significant.

In this work, we report the TS-couplings observed in the NMR spectra of several 2′-fluoro-substituted acetophenone derivatives. Additional DFT calculations and X-ray structural analyses supported the preference of the *s*-*trans* conformers of these derivatives. The conformational properties of these fluorinated compounds can give clues to the design of new drugs containing fluorine atoms.

## Results and Discussion

**Scheme 1:**
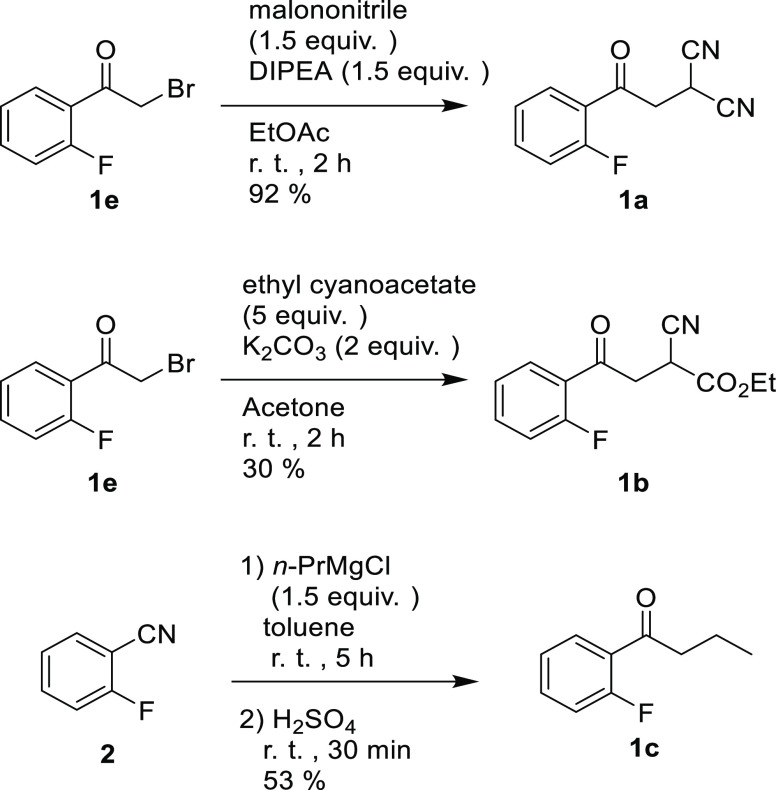
Synthesis of Propiophenone Derivatives **1a**, **1b**, and **1c**

Propiophenone derivatives **1a** and **1b** were prepared from 2-bromo-2′-fluoroacetophenone (**1e**)^8^ by treatment with malononitrile and ethyl cyanoacetate, respectively. 2′-Fluorobutyrophenone (**1c**) was prepared by reacting 2′-fluorobenzonitrile (**2**) with propylmagnesium chloride (Scheme 1). Compounds **3**,^9^**4**,^10^**5**,^11^ and **1d**–**p** were commercially available.

**Figure 2:**
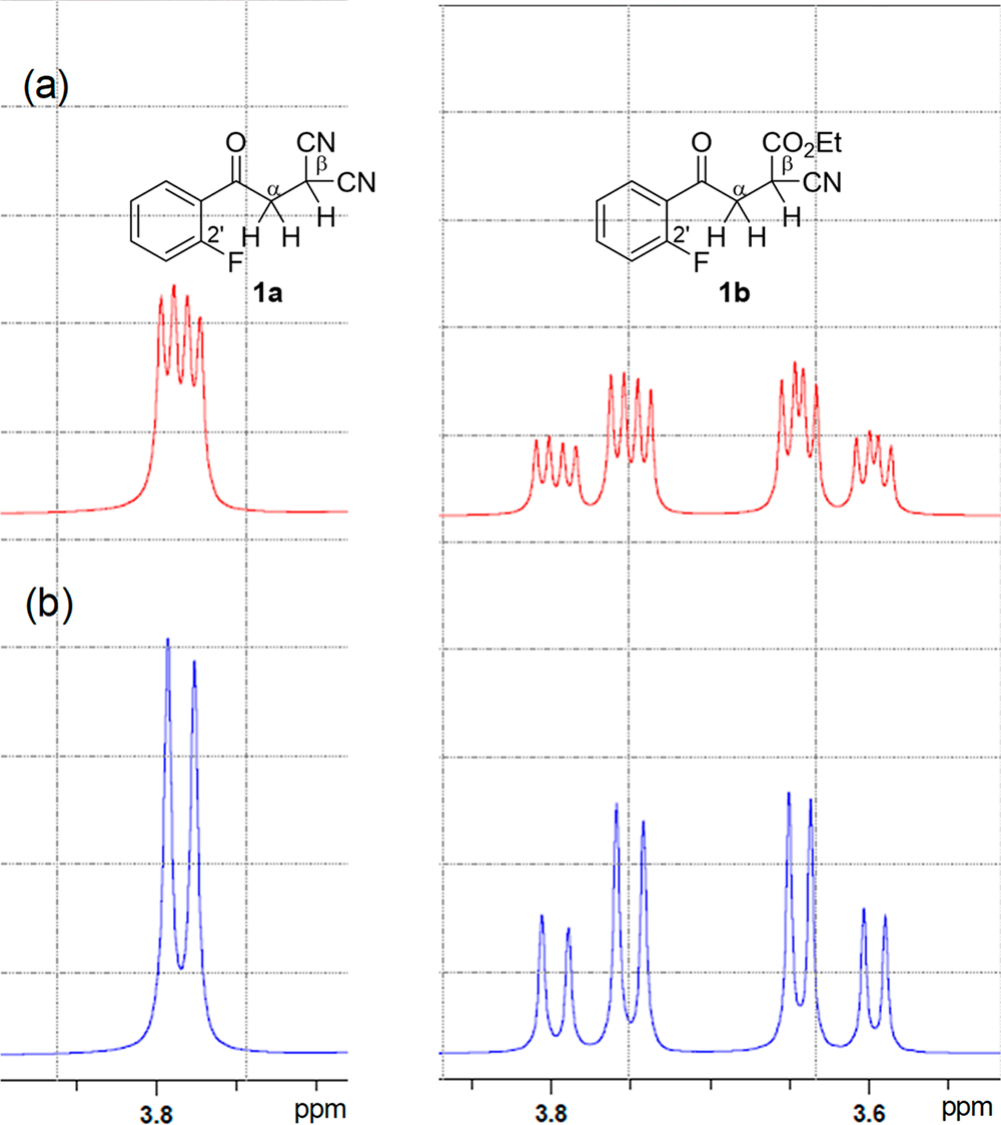
400 MHz NMR spectra in CDCl_3_: (a) H^α^ of **1a** (left) and H^α^ and H^α^′^^ of **1b** (right); (b) spectra with ^19^F decoupling of H^α^ of **1a** (left) and H^α^ and H^α^′^^ of **1b** (right).

Figure 2a,b shows the ^1^H NMR signals of H^α^ of **1a** and **1b**, respectively. A splitting pattern of the chemically equivalent methylene protons H^α^ of **1a** (Figure 2a, left) was observed as a doublet of doublets (dd), which was assumed to be the result of the coupling between H^α^ and H^β^(*J* = 6.9 Hz) and the additional coupling between H^α^ and F (TS-coupling: *J* = 3.2 Hz). Similarly, the AB part of the ABX signal of the diastereotopic methylene protons (H^α^ and H^α^′^^) (*J*_αα′_ = 18.8 Hz, *J*_αβ_ = 6.8 Hz, *J*_α′β_ = 6.4 Hz) of **1b** [Figure 2a, right] is further subjected to coupling with F (TS-coupling: *J*_αF_ = 3.3 Hz, *J*_α′F_ = 3.3 Hz).

To confirm that the splitting of the H^α^ of **1a** and **1b** is actually caused by F atoms, ^19^F-decoupled ^1^H NMR experiments were carried out. As shown in Figure 2b, irradiation of ^19^F resulted in simplification of their signal patterns, and these experiments determined the H^α^–F coupling constants of **1a** and **1b** to be 3.2 and 3.3 Hz. These protons are 5 bonds apart from the fluorine. In general, the through-bond coupling constant (^5^*J*_FH_) is less than 1 Hz, and the observed values of over 3.2 Hz infer that ^1^H–^19^F TS-couplings are working in **1a** and **1b**. In the proton-decoupled ^13^C NMR spectrum of **1a** and **1b**, the signals of the C^α^s were observed as doublets (^4^*J*_CF_ = 10.5 and 10.1 Hz), which were assumed to be caused by the TS-coupling between C^α^ and F (see the Supporting Information).

**Figure 3:**
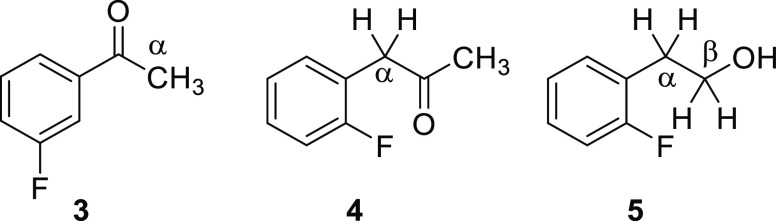
3′-Fluoroacetophenone (**3**), 2′-fluorophenylacetone (**4**), and 2-(2′-fluorophenyl)ethanol (**5**).

To confirm that such a TS-coupling is characteristic of 2′-fluoroacetophenones, the ^1^H NMR spectra of 3′-fluoroacetophenone (**3**), 2′-fluorophenylacetone (**4**), and 2′-fluorophenylethanol (**5**) (Figure 3) were studied. The methyl protons of **3** and **4** appear as sharp singlets without coupling with ^19^F because the methyl groups are distant from the fluorine. The β-methylene protons of **5**, although they are 5 bonds apart from the *ortho*-fluorine, show a mere triplet, possibly because of the flexible CH_2_–CH_2_ bond, which can position the β-CH_2_ spatially far from the fluorine. It should be noted that the α-CH_2_ of **4** and **5**, which are 4 bonds apart from F, do not show coupling with ^19^F; that is, through bond coupling, ^4^*J*_FH_ is negligible in these compounds. These properties are in contrast with those shown by **1a**–**c**, supporting the deduction that the *o*-fluoro-substituted benzoyl structure provides the *s*-*trans* conformation as a key factor for TS-couplings.

In order to confirm the generality, ^1^H/^13^C NMR spectra of other acetophenone derivatives (**1d**–**p**) were measured. As expected, relatively large H^α^–F and C^α^–F TS-couplings were observed (^5^*J*_HF_, 3.20–5.03 Hz; ^4^*J*_CF_, 6.70–11.56 Hz) (Table 1), which is in accordance with the assumption that the acetophenone derivatives **1a**–**p** prefer *s*-*trans* forms exclusively in solution.

**Table 1:**
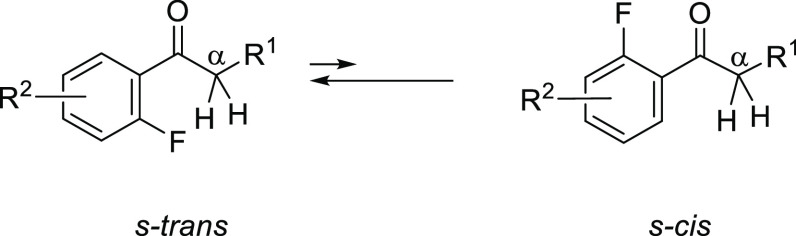
Through-Space Coupling Constants of Compounds **1a**–**p**

compound	R^1^	R^2^	*J* (H^α^, F) (Hz)	*J* (C^α^, F) (Hz)
**1a**	–CH(CN)_2_	–H	3.21	10.54
**1b**	–CH(CN, CO_2_Et)	–H	3.28	10.11
**1c**	–CH_2_CH_3_	–H	3.20	6.70
**1d**(12)	–H	–H	5.03	7.71
**1e**(8)	–Br	–H	3.20	9.63
**1f**(13)	–CH_3_	–H	3.20	7.71
**1g**(14)	–CO_2_CH_2_CH_3_	–H	3.66	8.67
**1h**(15)	–H	4′-Br	5.03	6.74
**1i**(16)	–H	5′-Br	5.03	7.71
**1j**(17)	–H	4′-OH	5.03	7.71
**1k**(18)	–H	4′-F	5.03	7.71
**1l**(19)	–H	4′,5′-F	5.03	7.71
**1m**(20)	–H	5′-NO_2_	5.03	6.74
**1n**(21)	–H	4′-OCH_3_	5.03	7.71
**1o**(22)	–CH_3_	4′-F	3.20	7.71
**1p**(23)	–Cl	4′-F	3.20	11.56

It is worth mentioning that TS-coupling in compounds **1a**–**p** is sensitive to the nature of the substituents at C^α^. While the acetophenones **1d** and **1h**–**n** with various substituents on their benzene rings have the same coupling constants (^5^*J*_HF_ = 5.03 Hz), those of **1a**–**g** (except for **1d**) and **1o**–**p**, in which C^α^ is variously substituted, are smaller in magnitude (^5^*J*_HF_: 3.20–3.66 Hz). These differences may be interpreted by the preceding report that the magnitude of TS-coupling depends not only on the distance between the nuclei but also on the orientation of the orbitals involved in the transmission pathway.^2b^ The substituents at C^α^ can affect the orbitals of H^α^ and C^α^ on determining the orientation and the transmission of the nuclei spin information through space.

**Figure 4:**
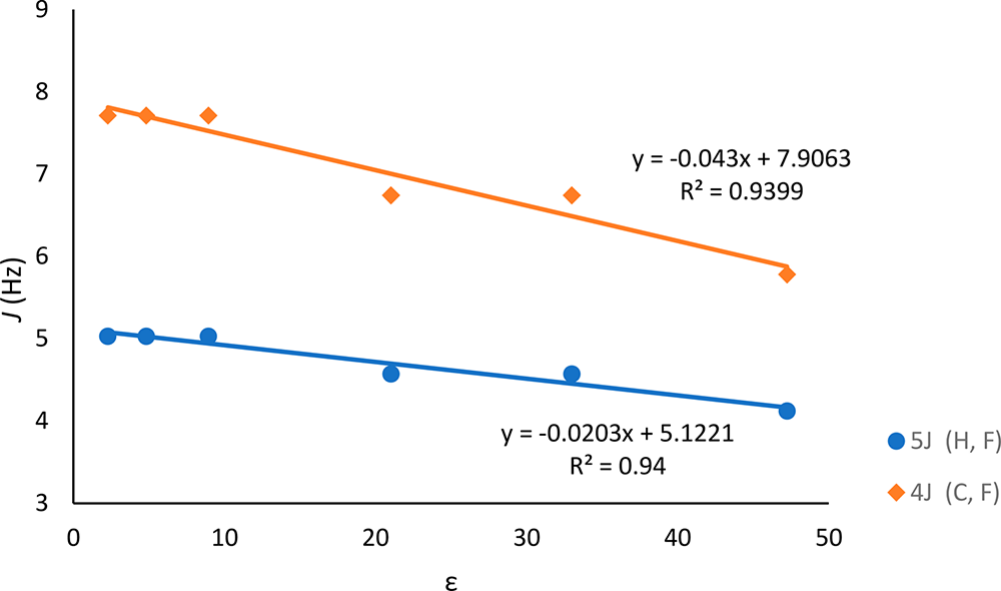
Plots of the coupling constants ^5^*J* (H, F) and ^4^*J* (C, F) observed in **1d** and the dielectric constant of the solvent.

Next, the solvent effect on the magnitude of TS-coupling was examined. In the ^1^H and ^13^C NMR spectra of 2′-fluoroacetophenone (**1d**), H^α^–F and C^α^–F TS-couplings were determined for the solutions in various solvents (Table 2). It is obvious from the large values of ^5^*J*_HF_ and ^4^*J*_CF_ that the *s*-*trans* conformer is fairly commonly preferred in any of these solutions. Furthermore, variation from low (benzene-*d*_6_, ε = 2.28) to high (DMSO-*d*_6_, ε = 47.2) dielectric constant solvents produced changes in the magnitudes of the coupling constants ^5^*J* (H^α^, F) and ^4^*J* (C^α^, F), which correlate linearly to the dielectric constant of the solvents (Figure 4).

**Table 2:** Solvent Effect on the Coupling Constant (Hz) of **1d**

solvent	ε^a^	^5^*J* (H^α^, F) (Hz)	^4^*J* (C^α^, F) (Hz)
DMSO-*d*_6_	47.2	4.12	5.78
CH_3_OH-*d*_4_	33.0	4.57	6.74
acetone-*d*_6_	21.0	4.57	6.74
CH_2_Cl_2_-*d*_2_	8.93	5.03	7.71
CHCl_3_-*d*_1_	4.81	5.03	7.71
benzene-*d*_6_	2.28	5.03	7.71

a ε = Dielectric constant.^24^

As mentioned above, the preference for the *s*-*trans* conformer of acetophenone derivatives **1a**–**p** was clarified. In order to obtain information on the stability of the *s*-*trans* conformation compared with the *s-cis* conformation, **1a**–**p** were analyzed by DFT calculations. First, the conformational ensembles of **1a**–**p** were generated from 2D chemical structures as the initial structures for the DFT calculations. These conformations generated were optimized with the RDKit using the universal force field (UFF) and clustered using a tolerance of 0.2 Å root-mean-square derivation. For each conformer, Hartree–Fock (HF) calculations were carried out to obtain optimized geometries and energies at the RHF/6-31G(d) and B3LYP/6-31G(d) levels. Due to insufficient formation of conformations in compounds **1d** and **1h**–**n**, we calculated the energy surfaces defined by a dihedral angle (∠O=C–C1′-C2′) to obtain stable conformers at the B3LYP/STO-3G level.

For the most stable structure in each *cis*/*trans* isomer, the geometries were further optimized at a more accurate level, i.e., RB3LYP/6-31G(d) on the SCRF/IEFPCM model in CHCl_3_ and RmPW1PW91/6-311G(d,p) on the SCRF/IEFPCM model in CHCl_3_. Zero-point energy (ZPE) correction was made on the basis of the frequency calculation with RmPW1PW91/6-311G(d,p) on the SCRF/IEFPCM model in CHCl_3_. As expected, the DFT calculation for **1a**–**p** confirmed that *trans* conformers are more stable than *cis* conformers. Further, using the energy differences (Δ*G*) between *cis*/*trans* conformers calculated by DFT, the ratios (*cis*/*trans*) based on the Boltzmann distribution were also calculated (Table 3). It was revealed that compounds **1a**–**p** exist predominantly as *trans* conformers.

**Table 3:**
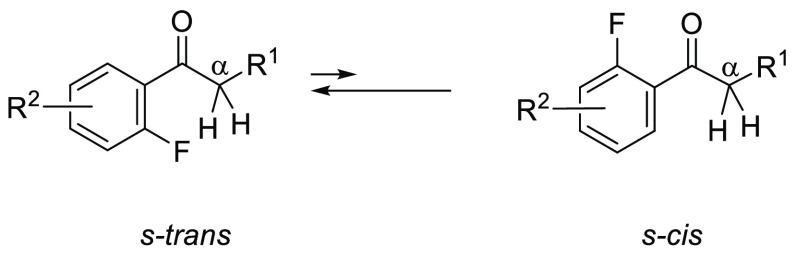
Difference in Energy and Ratio (*cis*/*trans*) of Compounds **1a**–**p** Calculated at mPW1PW91/6-311G(d,p), IEFPCM: CHCl_3._

compound	R^1^	R^2^	Δ*G*_*trans*/*cis*_ (kcal/mol)	*trans*/*cis*
**1a**	–CH(CN)_2_	–H	2.57	99:1
**1b**	–CH(CN, CO_2_Et)	–H	2.91	99:1
**1c**	–CH_2_CH_3_	–H	2.28	98:2
**1d**	–H	–H	3.56	>99:1
**1e**	–Br	–H	2.09	97:3
**1f**	–CH_3_	–H	4.13	>99:1
**1g**	–CO_2_CH_2_CH_3_	–H	2.67	99:1
**1h**	–H	4′-Br	3.51	>99:1
**1i**	–H	5′-Br	3.60	>99:1
**1j**	–H	4′-OH	2.40	98:2
**1k**	–H	4′-F	1.99	97:3
**1l**	–H	4′,5′-F	2.48	99:1
**1m**	–H	5′-NO_2_	3.02	99:1
**1n**	–H	4′-OCH_3_	1.75	95:5
**1o**	–CH_3_	4′-F	2.95	99:1
**1p**	–Cl	4′-F	3.23	>99:1

When these *s*-*trans* conformations of compounds **1a**–**p** were optimized at the mPW1PW91/6-311G(d,p) level, the H^α^–F and C^α^–F internuclear distances of **1a**–**p** were estimated (Table 4). In all cases, H^α^–F internuclear distances are smaller than the sum of van der Waals radii of fluorine and hydrogen (∼2.67 × 10^–10^ m), and C^α^–F distances are also smaller than that of fluorine and carbon (∼3.23 × 10^–10^ m).^25^

**Table 4:** H^α^–Fand C^α^–F Internuclear Distances of the *s-trans* Conformations of Compounds **1a**–**p** Calculated at mPW1PW91/6-311G(d,p), IEFPCM: CHCl_3_

			internuclear distance (10^–10^ m)	
compound	R^1^	R^2^	H^α^–F	C^α^–F
**1a**	–CH(CN)_2_	–H	2.40	2.71
**1b**	–CH(CN, CO_2_Et)	–H	2.43	2.73
**1c**	–CH_2_CH_3_	–H	2.43	2.76
**1d**	–H	–H	2.48	2.74
**1e**	–Br	–H	2.25	2.81
**1f**	–CH3	–H	2.43	2.76
**1g**	–CO_2_CH_2_CH_3_	–H	2.38	2.75
**1h**	–H	4′-Br	2.48	2.75
**1i**	–H	5′-Br	2.48	2.75
**1j**	–H	4′-OH	2.48	2.75
**1k**	–H	4′-F	2.48	2.75
**1l**	–H	4′,5′-F	2.49	2.76
**1m**	–H	5′-NO_2_	2.50	2.76
**1n**	–H	4′-OCH_3_	2.49	2.75
**1o**	–CH_3_	4′-F	2.43	2.77
**1p**	–Cl	4′-F	2.30	2.81

**Figure 5:**
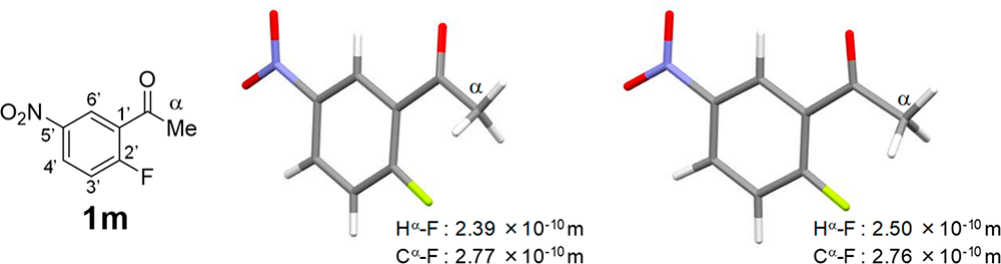
X-ray crystal structure (left) and the calculated one optimized by calculation at mPW1PW91/6-311G(d,p), IEFPCM: CHCl_3_ (right) of **1m**.

**Figure 6:**
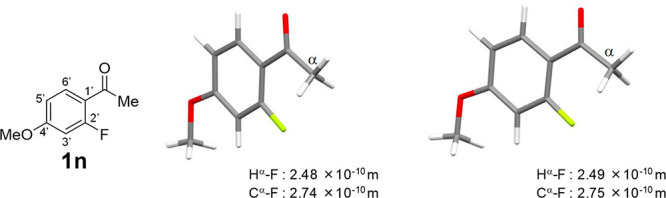
X-ray crystal structure (left) and the calculated one optimized by calculation at mPW1PW91/6-311G(d,p), IEFPCM: CHCl_3_ (right) of **1n**.

Since acetophenone derivatives **1m** and **1n** were obtained as single crystals, the solid states were examined by X-ray crystallography. In each crystal, only the *s-trans* conformer was present (Figures 5 and 6, left). The H^α^–F and C^α^–F internuclear distances of compounds **1m** and **1n** were measured (**1m**: H^α^–F = 2.39 × 10^–10^ m, C^α^–F = 2.77 × 10^–10^ m; **1n**: H^α^–F = 2.48 × 10^–10^ m, C^α^–F = 2.74 × 10^–10^ m), which were smaller than the sum of van der Waals radii of fluorine and hydrogen and that of fluorine and carbon. In Figures 5 and 6 (right), *s-trans* conformers of **1m** and **1n** as calculated by the DFT method reflecting the contribution of CHCl_3_ are shown for comparison. The structures and the H^α^–F and C^α^–F internuclear distances obtained by calculation are very similar to those of the solid state. Additionally, it was found that the benzene ring and carbonyl group are almost coplanar. The dihedral angle C2′–C1′-C=O of the solid state of compound **1m** is 169.9° and that of **1n** is 179.8°.

**Figure 7:**
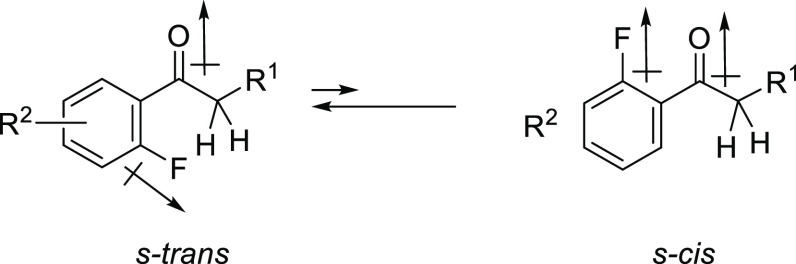
Conformational property of 2′-fluoroacetophenone derivatives.

All of these findings make it clear that 2′-fluoroacetophenone derivatives form *s*-*trans* conformations exclusively, and as a result, H^α^–F and C^α^–F TS-couplings are observed in their NMR spectra. A high polarization of C^δ+^–F^δ−^ and the presence of three lone pairs on fluorine might suggest that the fluorine of the C–F bond could act as a hydrogen bond acceptor. However, it is known that fluorine in organic molecules forms relatively weak hydrogen bonds. The H^α^–F internuclear distances of compounds **1m** and **1n** in the crystal state were 2.39 × 10^–10^ m and 2.48 × 10^–10^ m, respectively (Figures 5 and 6). Such relatively long distances, meaning a weaker interaction compared with a typical hydrogen bond (e.g., ROH···O=C ∼ 1.9 × 10^–10^ m),^1b^ give less conclusive proof of the conformational preference. The understanding of this phenomenon requires a discussion of the ionic nature of the C–F bond, which causes a large dipole moment (μ). The dipole of the C–F bond plays a significant part in determining the conformational behavior of fluorinated organic molecules. For example, α-fluorocarbonyl compounds prefer a conformation where the C–F bond lies *anti*-periplanar to the carbonyl group, in which carbonyl and C–F dipoles oppose each other to minimize the dipole of the entire molecule.^26^ Based on this point of view, the *s-cis* conformation where the C–F bond lies *syn*-periplanar to the carbonyl group should maximize the dipole of the entire molecule, which makes the *s-cis* conformation unstable. On the other hand, *s*-*trans* conformers, in which the benzene ring and carbonyl group are almost coplanar, minimize the repulsive dipoles of the C–F bond and carbonyl group. As a result, acetophenone derivatives **1a**–**p** might prefer *s*-*trans* conformers to *cis* conformers (Figure 7).

## Conclusion

H^α^–F and C^α^–F TS-couplings were observed in the NMR spectra of 2′-fluoro-substituted acetophenone derivatives **1a**–**p**, and the over whelming *s*-*trans* conformational preference was elucidated. The magnitudes of the coupling constants ^5^*J* (H^α^, F) and ^4^*J* (C^α^, F) correlate with the nature of the substituents at C^α^ and the value of the dielectric constant of solvents. Additionally, X-ray structural analysis suggested that the benzene ring and carbonyl group are almost coplanar in the *s*-*trans* conformation, which makes the H^α^–F and C^α^–F internuclear distances smaller than the sum of their van der Waals radii. Such conformations were reproduced with DFT calculations. Considering the ionic nature of the C–F bond, which causes a large dipole moment (μ), it was assumed that the *s*-*trans* conformation, in which the C–F dipole detaches from the carbonyl group repulsively, minimizes the dipole of the entire molecule. The dipole of the C–F bond must play a significant part in determining the conformational behavior of 2′-fluoro-substituted acetophenones. The 2′-fluoro-substituted acetophenones with the preferable *s*-*trans* conformations are expected to be utilized as new basic scaffolds for the design of bioactive compounds in medicinal chemistry in the future.

## Experimental Section

### General Information

Materials were obtained from commercial suppliers. Although all of the fluoro compounds in this work are known and their NMR data have been presented, the more detailed NMR properties, which we newly determined, were defined in order to demonstrate TS-coupling. NMR spectra were recorded on a spectrometer at 400 or 600 MHz for ^1^H NMR and 100 or 150 MHz for ^13^C NMR. Chemical shifts are given in parts per million (ppm) downfield from tetramethylsilane as an internal standard, and coupling constants (*J*) are reported in hertz (Hz). Splitting patterns are abbreviated as follows: singlet (s), doublet (d), triplet (t), quartet (q), multiplet (m), and broad (br). IR spectra were recorded on an FT-IR spectrometer equipped with ATR (Diamond). The high-resolution mass spectra (HRMS) were recorded on a TOF-MS instrument with an ionization mode of ESI and APCI. Melting points were recorded on a melting point apparatus and are uncorrected. Analytical thin-layer chromatography was performed on precoated, glass-backed silica gel plates. Column chromatography was performed using silica gel (45–60 μm). Extracted solutions were dried over anhydrous MgSO_4_ or Na_2_SO_4_. Solvents were evaporated under reduced pressure. Since compounds **3**, **4**, **5**, and **1d**–**p** were commercially available, characterization data of ^1^H NMR and ^13^C NMR were described.

#### 2-[2-(2-Fluorophenyl)-2-oxoethyl]propanedinitrile (**1a**)

To 2-bromo-2′-fluoroacetophenone (1.81 mL, 13.1 mmol) in ethyl acetate (EtOAc) (17 mL) were added malononitrile (1.24 mL, 19.7 mmol) and diisopropylethylamine (3.42 mL, 19.7 mmol) at 0 °C under an argon stream, and the mixture was stirred at room temperature for 3 h. Then aq. NH_4_Cl was added, the mixture was extracted with EtOAc, and the extract was washed with brine, dried over Na_2_SO_4_, filtered, and concentrated under reduced pressure. The concentrate was dried in vacuo and purified by silica gel column chromatography (hexane/dichloromethane = 1:1) to afford **1a**, 2.44 g, yield 92%, as colorless crystals.

^1^H NMR (400 MHz, CDCl_3_, ppm): δ 8.02 (ddd, *J* = 7.2, 7.2, 2.0 Hz, 1H), 7.639–7.63 (m, 1H), 7.32 (dd, *J* = 7.2, 7.2 Hz, 1H), 7.22 (dd, *J* = 11.6, 8.4 Hz, 1H), 4.38 (t, *J* = 6.9 Hz, 1H), 3.76 (dd, *J* = 6.9, 3.2 Hz, 2H). ^13^C{^1^H} NMR (100 MHz, CDCl_3_, ppm): δ 189.5, 162.6 (C–F, ^1^*J*_C–F_ = 255.9 Hz), 136.7 (C–F, ^3^*J*_C–F_ = 9.6 Hz), 131.0, 125.1 (C–F, ^3^*J*_C–F_ = 2.9 Hz), 122.6 (C–F, ^2^*J*_C–F_ = 12.5 Hz), 117.0 (C–F, ^2^*J*_C–F_ = 24.0 Hz). 112.3, 43.7 (C–F, ^4^*J*_C–F_ = 10.5 Hz), 17.7 (C–F, ^5^*J*_C–F_ = 3.8 Hz). IR-ATR: 2260, 1675 cm^–1^. HRMS (APCI-TOF) *m*/*z*: [(M – H)^−^] calcd for C_11_H_6_N_2_OF^–^, 201.0470; found, 201.0451.

#### Ethyl 2-Cyano-4-(2-fluorophenyl)-4-oxobutanoate (**1b**)

To ethyl cyanoacetate (615 μL, 5.77 mmol) in tetrahydrofuran (THF) (1 mL) was added K_2_CO_3_ powder (1.2 g, 8.66 mmol), and the mixture was stirred at 40–45 °C (oil bath) for 30 min. To this stirred suspension was slowly added 2-bromo-2′-fluoroacetophenone (1 mL, 6.35 mmol) in THF (5.1 mL) dropwise over a period of 20 min at room temperature, and then the mixture was stirred at room temperature for 14 h, filtered, and concentrated under reduced pressure. The residue was extracted with EtOAc, and the extract was washed with brine, dried over Na_2_SO_4_, filtered, and concentrated in vacuo. The resulting residue was purified by silica gel column chromatography (hexane/EtOAc = 4:1) to yield compound **1b**, 0.52 g, 36%, as a yellow oil.

^1^H NMR (400 MHz, CDCl_3_, ppm): δ 7.95 (ddd, *J* = 7.6, 7.6, 1.6 Hz, 1H), 7.62–7.57 (m, 1H), 7.27 (ddd, *J* = 7.6, 7.6, 0.8 Hz, 1H), 7.19 (ddd, *J* = 11.6, 8.4, 0.8 Hz, 1H), 4.31 (q, *J* = 7.2 Hz, 2H), 4.11 (t, *J* = 6.4 Hz, 1H), 3.76 (ddd, *J* = 18.8, 6.4, 3.3 Hz, 1H), 3.60 (ddd, *J* = 18.8, 6.8, 3.3 Hz, 1H), 1.35 (t, *J* = 7.2 Hz, 3H). ^13^C{^1^H} NMR (100 MHz, CDCl_3_, ppm): δ 192.2, 165.3, 162.5 (CF, ^1^*J*_C–F_ = 255.8 Hz), 135.9 (C–F, ^3^*J*_C–F_ = 10.1 Hz), 130.9, 124.8 (C–F, ^3^*J*_C–F_ = 2.9 Hz), 123.6 (C–F, ^2^*J*_C–F_ = 11.6 Hz), 116.9 (C–F, ^2^*J*_C–F_ = 24.6 Hz), 116.2, 63.2, 42.5 (C–F, ^4^*J*_C–F_ = 10.1 Hz), 32.0 (C–F, ^5^*J*_C–F_ = 2.9 Hz), 13.9. IR-ATR: 2254, 1744, 1686 cm^–1^. HRMS (ESI-TOF) *m*/*z*: [(M + Na)^+^] calcd for C_13_H_12_NO_3_FNa, 272.0693; found, 272.0698.

#### 1-(2-Fluorophenyl)-1-butanone (**1c**)

2-Fluorobenzenitrile (1 mL, 9.4 mmol) was added to a solution of propylmagnesium chloride (9.4 mL, 18.8 mmol) in toluene (8 mL) under ice cooling, and the mixture was stirred at room temperature for 4.5 h. Sulfuric acid (2 mL) was carefully poured into the mixture under ice cooling, and the mixture was stirred at room temperature for 30 min. The mixture was then extracted with EtOAc, and the extract was washed with brine, dried over Na_2_SO_4_, filtered, and concentrated in vacuo. The resulting residue was purified by silica gel column chromatography (hexane/EtOAc = 20:1) to yield compound **1c**, 0.84 g, 53%, as a colorless oil.

^1^H NMR (400 MHz, CDCl_3_, ppm): δ 7.84 (ddd, *J* = 9.6, 7.6, 1.6 Hz, 1H), 7.53–7.47 (m, 1H), 7.22 (ddd, *J* = 7.6, 7.6, 0.8 Hz, 1H), 7.13 (ddd, *J* = 11.2, 8.0, 0.8 Hz, 1H), 2.93 (dt, *J* = 7.2, 3.2 Hz, 2H), 1.75 (sext, *J* = 7.2 Hz, 2H), 0.98 (t, *J* = 7.2 Hz, 3H). ^13^C{^1^H} NMR (100 MHz, CDCl_3_, ppm): δ 198.9, 161.8 (C–F, ^1^*J*_C–F_ = 254.3 Hz), 134.2 (C–F, ^3^*J*_C–F_ = 8.7 Hz), 130.6, 125.9 (C–F, ^2^*J*_C–F_ = 13.5 Hz), 124.4, 116.6 (C–F, ^2^*J*_C–F_ = 24.1 Hz), 45.5 (C–F, ^4^*J*_C–F_ = 6.7 Hz), 17.4, 13.8. IR-ATR: 1686 cm^–1^. HRMS (APCI-TOF) *m*/*z*: [(M + H)^+^] calcd for C_10_H_12_OF, 167.0867; found, 167.0873.

#### 1-(2-Fluorophenyl)ethanone (**1d**)

Colorless oil. ^1^H NMR (400 MHz, CDCl_3_, ppm): δ 7.88 (ddd, *J* = 8.0, 8.0, 2.0 Hz, 1H), 7.55–7.50 (m, 1H), 7.23 (ddd, *J* = 8.0, 8.0, 0.8 Hz, 1H), 7.14 (ddd, *J* = 11.6, 7.2, 0.8 Hz, 1H), 2.65 (d, *J* = 5.0 Hz, 3H). ^13^C{^1^H} NMR (100 MHz, CDCl_3_, ppm): δ 196.0, 162.2 (C–F, ^1^*J*_C–F_ = 254.3 Hz), 134.7 (C–F, ^3^*J*_C–F_ = 8.7 Hz), 130.6, 125.7 (C–F, ^2^*J*_C–F_ = 12.5 Hz), 124.4 (C–F, ^3^*J*_C–F_ = 2.9 Hz), 116.6 (C–F, ^2^*J*_C–F_ = 23.1 Hz), 31.5 (C–F, ^4^*J*_C–F_ = 7.7 Hz).

#### 2-Bromo-1-(2-fluorophenyl)ethanone (**1e**)

White crystalline solid. ^1^H NMR (400 MHz, CDCl_3_, ppm): δ 7.95 (ddd, *J* = 7.6, 7.6, 1.6 Hz, 1H), 7.61–7.56 (m, 1H), 7.28 (dd, *J* = 11.2, 11.2 Hz, 1H), 7.19 (dd, *J* = 11.2, 11.2 Hz, 1H), 4.53 (d, *J* = 3.2 Hz, 2H). ^13^C{^1^H} NMR (100 MHz, CDCl_3_, ppm): δ 189.1, 161.7 (C–F, ^1^*J*_C–F_ = 254.3 Hz), 135.6 (C–F, ^3^*J*_C–F_ = 9.6 Hz), 131.5, 124.9 (C–F, ^3^*J*_C–F_ = 2.9 Hz), 122.8 (C–F, ^2^*J*_C–F_ = 13.5 Hz), 116.7 (C–F, ^2^*J*_C–F_ = 24.1 Hz), 36.0 (C–F, ^4^*J*_C–F_ = 9.6 Hz).

#### 1-(2-Fluorophenyl)propanone (**1f**)

Colorless oil. ^1^H NMR (400 MHz, CDCl_3_, ppm): δ 7.87 (ddd, *J* = 7.8, 7.8, 1.8 Hz, 1H), 7.53–7.48 (m, 1H), 7.22 (ddd, *J* = 7.8, 7.8, 0.9 Hz, 1H), 7.13 (ddd, *J* = 11.4, 8.7, 0.9 Hz, 1H), 3.01 (dq, *J* = 7.2, 3.2 Hz, 2H), 1.21 (t, *J* = 7.2 Hz, 3H). ^13^C{^1^H} NMR (100 MHz, CDCl_3_, ppm): δ 199.3 (C–F, ^3^*J*_C–F_ = 3.9 Hz), 161.9 (C–F, ^1^*J*_C–F_ = 253.4 Hz), 134.3 (C–F, ^3^*J*_C–F_ = 8.7 Hz), 130.6 (C–F, ^4^*J*_C–F_ = 2.9 Hz), 125.7 (C–F, ^2^*J*_C–F_ = 13.5 Hz), 124.4 (C–F, ^3^*J*_C–F_ = 3.9 Hz), 116.6 (C–F, ^2^*J*_C–F_ = 23.1 Hz), 36.8 (C–F, ^4^*J*_—F_ = 7.7 Hz), 8.0.

#### Ethyl 3-(2-Fluorophenyl)-3-oxopropanoate (**1g**) (2:1 Mixture of Keto and Enol Tautomers)

Colorless oil. ^1^H NMR (400 MHz, CDCl_3_, ppm): δ 7.94 (ddd, *J* = 7.6, 7.6, 2.0 Hz, 1H), 7.87 (ddd, *J* = 8.0, 8.0, 2.0 Hz, 0.5 Hz), 7.57–7.53 (m, 1H), 7.42–7.40 (m, 0.5H), 7.29–7.20 (m, 2H), 7.17–7.09 (m, 1H), 5.84 (s, 0.5H), 4.27 (q, *J* = 7.2 Hz, 1H), 4.21 (q, *J* = 7.2 Hz, 2H), 3.99 (d, *J* = 3.7 Hz, 2H), 1.34 (t, *J* = 7.2 Hz, 1.5H), 1.25 (t, *J* = 7.2 Hz, 3H). ^13^C{^1^H} NMR (100 MHz, CDCl_3_, ppm): δ 190.3 (C–F, ^3^*J*_C–F_ = 3.9 Hz), 173.3, 167.4, 162.2 (C–F, ^1^*J*_C–F_ = 254.3 Hz), 135.4 (C–F, ^3^*J*_C–F_ = 8.7 Hz), 132.3 (C–F, ^3^*J*_C–F_ = 9.6 Hz), 130.9 (C–F, ^4^*J*_C–F_ = 1.9 Hz), 129.2, 124.7 (C–F, ^3^*J*_C–F_ = 2.9 Hz), 124.6 (C–F, ^2^*J*_C–F_ = 12.5 Hz), 124.3 (C–F, ^2^*J*_C–F_ = 13.5 Hz), 124.3 (C–F, ^3^*J*_C–F_ = 3.9 Hz), 116.5 (C–F, ^2^*J*_C–F_ = 24.1 Hz), 116.4 (C–F, ^2^*J*_C–F_ = 24.1 Hz), 92.6 (C–F, ^4^*J*_C–F_ = 13.5 Hz), 61.3, 60.5, 49.9 (C–F, ^4^*J*_C–F_ = 8.7 Hz), 14.2, 14.0.

#### 1-(4-Bromo-2-fluorophenyl)ethanone (**1h**)

White crystalline solid. ^1^H NMR (400 MHz, CDCl_3_, ppm): δ 7.77 (dd, *J* = 8.4, 8.4 Hz, 1H), 7.40–7.35 (m, 2H), 2.63 (d, *J* = 5.0 Hz, 3H). ^13^C{^1^H} NMR (100 MHz, CDCl_3_): δ 194.7 (C–F, ^3^*J*_C–F_ = 2.9 Hz), 161.8 (C–F, ^1^*J*_C–F_ = 259.1 Hz), 131.7 (C–F, ^4^*J*_C–F_ = 2.9 Hz), 128.2 (C–F, ^3^*J*_C–F_ = 10.6 Hz), 128.0 (C–F, ^3^*J*_C–F_ = 3.9 Hz), 124.5 (C–F, ^2^*J*_C–F_ = 13.5 Hz), 120.7 (C–F, ^2^*J*_C–F_ = 27.9 Hz), 31.4 (C–F, ^4^*J*_C–F_ = 6.7 Hz).

#### 1-(5-Bromo-2-fluorophenyl)ethanone (**1i**)

Pale yellow crystalline solid. ^1^H NMR (400 MHz, CDCl_3_, ppm): δ 7.99 (dd, *J* = 6.4, 2.8 Hz, 1H), 7.63–7.59 (m, 1H), 7.05 (dd, *J* = 10.0, 10.0 Hz, 1H), 2.64 (d, *J* = 5.0 Hz, 3H). ^13^C{^1^H} NMR (100 MHz, CDCl_3_, ppm): δ 194.4, 161.2 (C–F, ^1^*J*_C–F_ = 255.2 Hz), 137.3 (C–F, ^3^*J*_C–F_ = 8.7 Hz), 133.3 (C–F, ^3^*J*_C–F_ = 2.9 Hz), 127.1 (C–F, ^2^*J*_C–F_ = 14.5 Hz), 118.6 (C–F, ^2^*J*_C–F_ = 26.0 Hz), 117.3, 31.3 (C–F, ^4^*J*_C–F_ = 7.7 Hz).

#### 1-(2-Fluoro-4-hydroxyphenyl)ethanone (**1j**)

Pale pink crystalline solid. ^1^H NMR (400 MHz, CDCl_3_, ppm): δ 7.85 (dd, *J* = 8.8, 8.8 Hz, 1H), 6.68 (dd, *J* = 8.8, 2.4 Hz, 1H), 6.39 (dd, *J* = 12.4, 2.4 Hz, 1H), 5.78 (d, *J* = 0.9 Hz, 1H), 2.60 (d, *J* = 5.0 Hz, 3H). ^13^C{^1^H} NMR (100 MHz, CDCl_3_, ppm): δ 196.1, 164.2 (C–F, ^1^*J*_C–F_ = 256.2 Hz), 162.3 (C–F, ^3^*J*_C–F_ = 12.5 Hz), 132.5 (C–F, ^3^*J*_C–F_ = 3.9 Hz), 118.2 (C–F, ^2^*J*_C–F_ = 12.5 Hz), 112.2, 103.6 (C–F, ^2^*J*_C–F_ = 27.0 Hz), 31.1 (C–F, ^4^*J*_C–F_ = 7.7 Hz).

#### 1-(2,4-Difluorophenyl)ethanone (**1k**)

Colorless oil. ^1^H NMR (400 MHz, CDCl_3_, ppm): δ 7.94 (ddd, *J* = 8.4, 8.4, 6.8 Hz, 1H), 6.98–6.93 (m, 1H), 6.88 (ddd, *J* = 10.8, 8.8, 2.8 Hz, 1H), 2.63 (d, *J* = 5.0 Hz, 3H). ^13^C{^1^H} NMR (100 MHz, CDCl_3_, ppm): δ 194.3 (C–F, ^3^*J*_C–F_ = 3.9 Hz), 165.9 (C–F, ^1^*J*_C–F_ = 257.2, ^3^*J*_C–F_ = 12.5 Hz), 163.0 (C–F, ^1^*J*_C–F_ = 258.2 Hz, ^3^*J*_C–F_ = 12.5 Hz), 132.6 (C–F, ^2^*J*_C–F_ = 10.6 Hz, ^4^*J*_C–F_ = 3.9 Hz), 122.2 (C–F, ^3^*J*_C–F_ = 13.5 Hz), 112.1 (C–F, ^2^*J*_C–F_ = 22.2 Hz, ^4^*J*_C–F_ = 3.9 Hz), 104.7 (C–F, ^2^*J*_C–F_ = 27.9 Hz, ^2^*J*_C–F_ = 27.0 Hz), 31.3 (C–F, ^4^*J*_C–F_ = 7.7 Hz).

#### 1-(2,4,5-Trifluorophenyl)ethanone (**1l**)

Colorless oil. ^1^H NMR (400 MHz, CDCl_3_, ppm): δ 7.79–7.72 (m, 1H), 7.02 (ddd, *J* = 10.0, 10.0, 6.0 Hz, 1H), 2.63 (d, *J* = 5.0 Hz, 3H). ^13^C{^1^H} NMR (100 MHz, CDCl_3_, ppm): δ 193.1 (C–F, ^3^*J*_C–F_ = 3.9 Hz), 157.9 (C–F, ^1^*J*_C–F_ = 252.4 Hz, ^3^*J*_C–F_ = 9.6 Hz, ^4^*J*_C–F_ = 1.9 Hz), 153.3 (C–F, ^1^*J*_C–F_ = 260.1 Hz, ^2^*J*_C–F_ = 15.4 Hz, ^3^*J*_C–F_ = 12.5 Hz), 147.1 (C–F, ^1^*J*_C–F_ = 247.6 Hz, ^2^*J*_C–F_ = 12.5 Hz, ^4^*J*_C–F_ = 3.9 Hz), 122.0 (C–F, ^2^*J*_C–F_ = 11.6 Hz, ^3^*J*_C–F_ = 3.9 Hz), 118.4 (C–F, ^2^*J*_C–F_ = 20.2 Hz, ^3^*J*_C–F_ = 3.8 Hz, ^3^*J*_C–F_ = 3.8 Hz), 106.7 (C–F, ^2^*J*_C–F_ = 30.8 Hz, ^2^*J*_C–F_ = 30.8 Hz), 31.3 (C–F, ^4^*J*_C–F_ = 7.7 Hz).

#### 1-(2-Fluoro-5-nitrophenyl)ethanone (**1m**)

Pale yellow crystalline solid. ^1^H NMR (400 MHz, CDCl_3_, ppm): δ 8.78 (dd, *J* = 6.0, 2.8 Hz, 1H), 8.43–8.39 (m, 1H), 7.34 (dd, *J* = 9.6, 9.2 Hz, 1H), 2.70 (d, *J* = 5.0 Hz, 3H). ^13^C{^1^H} NMR (100 MHz, CDCl_3_, ppm): δ 193.3, 165.0 (C–F, ^1^*J*_C–F_ = 264.9 Hz), 144.5, 129.4 (C–F, ^3^*J*_C–F_ = 10.6 Hz), 126.9 (C–F, ^3^*J*_C–F_ = 3.9 Hz), 126.4 (C–F, ^2^*J*_C–F_ = 16.4 Hz), 118.3 (C–F, ^2^*J*_C–F_ = 26.0 Hz), 31.2 (C–F, ^4^*J*_C–F_ = 6.7 Hz).

#### 1-(2-Fluoro-4-methoxyphenyl)ethanone (**1n**)

White crystalline solid. ^1^H NMR (400 MHz, CDCl_3_, ppm): δ 7.89 (dd, *J* = 8.8, 8.8 Hz, 1H), 6.70 (dd, *J* = 8.8, 2.0 Hz, 1H), 6.62 (dd, 13.2, 2.0 Hz, 1H), 3.86 (s, 3H), 2.60 (d, *J* = 5.0 Hz, 3H). ^13^C{^1^H} NMR (100 MHz, CDCl_3_, ppm): δ 194.5, 164.9 (C–F, ^3^*J*_C–F_ = 11.6 Hz), 163.8 (C–F, ^1^*J*_C–F_ = 255.3 Hz), 132.1 (C–F, ^3^*J*_C–F_ = 3.9 Hz), 118.6 (C–F, ^2^*J*_C–F_ = 13.5 Hz), 110.6, 101.6 (C–F, ^2^*J*_C–F_ = 27.9 Hz), 55.8, 31.2 (C–F, ^4^*J*_C–F_ = 7.7 Hz).

#### 1-(2,4-Difluorophenyl)propanone (**1o**)

Colorless oil. ^1^H NMR (400 MHz, CDCl_3_, ppm): δ 7.96–7.90 (m, 1H), 6.94 (dddd, *J* = 8.6, 7.6, 2.4, 0.8 Hz, 1H), 6.85 (ddd, *J* = 11.2, 8.8, 2.4 Hz, 1H), 2.97 (dq, *J* = 6.8, 3.2 Hz, 2H), 1.19 (dt, *J* = 6.8, 1.2 Hz, 3H). ^13^C{^1^H} NMR (100 MHz, CDCl_3_, ppm): δ 197.6 (C–F, ^3^*J*_C–F_ = 4.8 Hz), 165.7 (C–F, ^1^*J*_C–F_ = 256.2 Hz, ^3^*J*_C–F_ = 12.5 Hz), 162.8 (C–F, ^1^*J*_C–F_ = 257.2, ^3^*J*_C–F_ = 12.5 Hz), 132.6 (C–F, ^3^*J*_C–F_ = 10.6 Hz, ^3^*J*_C–F_ = 10.6 Hz), 122.1 (C–F, ^2^*J*_C–F_ = 13.5 Hz), 112.1 (C–F, ^2^*J*_C–F_ = 21.2 Hz, ^4^*J*_C–F_ = 2.9 Hz), 104.7 (C–F, ^2^*J*_C–F_ = 26.0 Hz, ^2^*J*_C–F_ = 26.0 Hz), 36.7 (C–F, ^4^*J*_C–F_ = 7.7 Hz), 7.92.

#### 2-Chloro-1-(2,4-difluorophenyl)ethanone (**1p**)

Pale brown crystalline solid. ^1^H NMR (400 MHz, CDCl_3_, ppm): δ 8.03 (ddd, *J* = 8.4, 8.4, 6.4 Hz, 1H), 7.05–7.00 (m, 1H), 6.92 (ddd, *J* = 11.2, 8.8, 2.4 Hz, 1H), 4.70 (d, *J* = 3.2 Hz, 2H). ^13^C{^1^H} NMR (100 MHz, CDCl_3_, ppm): δ 187.8, 166.5 (C–F, ^1^*J*_C–F_ = 259.2 Hz, ^3^*J*_C–F_ = 12.5 Hz), 162.6 (C–F, ^1^*J*_C–F_ = 257.2 Hz, ^3^*J*_C–F_ = 12.5 Hz), 133.3 (C–F, ^3^*J*_C–F_ = 10.6 Hz, ^3^*J*_C–F_ = 10.6 Hz), 119.4 (C–F, ^2^*J*_C–F_ = 14.5 Hz), 112.9 (C–F, ^2^*J*_C–F_ = 24.1 Hz, ^4^*J*_C–F_ = 2.9 Hz), 104.8 (C–F, ^2^*J*_C–F_ = 27.0 Hz, ^2^*J*_C–F_ = 26.0 Hz), 49.8 (C–F, ^4^*J*_C–F_ = 11.6 Hz).

#### 3′-Fluoroacetophenone (**3**)

Colorless oil. ^1^H NMR (400 MHz, CDCl_3_, ppm): δ 7.74 (ddd, *J* = 8.0, 1.6, 1.6 Hz, 1H), 7.64 (ddd, *J* = 9.6, 1.6, 1.6 Hz, 1H), 7.45 (ddd, *J* = 8.0, 8.0, 5.6 Hz, 1H), 7.29–7.23 (m, 1H), 2.60 (s, 3H). ^13^C{^1^H} NMR (100 MHz, CDCl_3_, ppm): δ 196.8 (C–F, ^3^*J*_C–F_ = 1.9 Hz), 162.8 (C–F, ^1^*J*_C–F_ = 248.5 Hz), 139.2 (C–F, ^3^*J*_—F_ = 5.8 Hz), 130.2 (C–F, ^3^*J*_C–F_ = 7.7 Hz), 124.1 (C–F, ^4^*J*_C–F_ = 2.9 Hz), 120.1 (C–F, ^2^*J*_C–F_ = 21.2 Hz), 115.0 (C–F, ^2^*J*_C–F_ = 22.2 Hz), 26.8.

#### 2′-Fluorophenylacetone (**4**)

Pale yellow oil. ^1^H NMR (400 MHz, CDCl_3_, ppm): δ 7.30–7.24 (m, 1H), 7.18 (ddd, *J* = 8.0, 8.0, 2.0 Hz, 1H), 7.13–7.05 (m, 2H), 3.74 (s, 2H), 2.20 (s, 3H). ^13^C{^1^H} NMR (100 MHz, CDCl_3_, ppm): δ 205.0, 161.0 (C–F, ^1^*J*_C–F_ = 245.6 Hz), 131.6 (C–F, ^3^*J*_C–F_ = 4.8 Hz), 129.0 (C–F, ^3^*J*_C–F_ = 8.7 Hz), 124.3 (C–F, ^4^*J*_C–F_ = 3.9 Hz), 121.6 (C–F, ^2^*J*_C–F_ = 16.4 Hz), 115.4 (C–F, ^2^*J*_C–F_ = 22.2 Hz), 43.9 (C–F, ^4^*J*_C–F_ = 1.93 Hz), 29.5.

#### 2-(2′-Fluorophenyl)ethanol (**5**)

Colorless oil. ^1^H NMR (400 MHz, CDCl_3_, ppm): δ 7.24–7.19 (m, 2H), 7.10–7.02 (m, 2H), 3.90 (t, *J* = 6.4 Hz, 2H), 2.92 (t, *J* = 6.4 Hz, 2H). ^13^C{^1^H} NMR (100 MHz, CDCl_3_, ppm): δ 161.3 (C–F, ^1^*J*_C–F_ = 244.7 Hz), 131.4 (C–F, ^3^*J*_C–F_ = 4.8 Hz), 128.2 (C–F, ^3^*J*_C–F_ = 7.7 Hz), 125.4 (C–F, ^2^*J*_C–F_ = 16.4 Hz), 124.1 (C–F, ^4^*J*_C–F_ = 3.9 Hz), 115.4 (C–F, ^2^*J*_C–F_ = 22.2 Hz), 62.6, 32.7.

## Supporting Information Available

^13^C NMR spectra of α-C of **1a** and **1b**, spectra of through-space coupling of **1a**–**p** and **3**–**5**, X-ray crystal data for **1m** and **1n**, DFT calculation study, and ^1^H and ^13^C{^1^H} NMR spectra of **1a**–**p** and **3**–**5** (PDF)

## Accession Codes

CCDC 2044642–2044643 contain the supplementary crystallographic data for this paper. These data can be obtained free of charge via www.ccdc.cam.ac.uk/data_request/cif, or by emailing data_request@ccdc.cam.ac.uk, or by contacting The Cambridge Crystallographic Data Centre, 12 Union Road, Cambridge CB2 1EZ, UK; fax: +44 1223 336033.
