# Supplementary material for: Conformational Preference of 2′-Fluoro-Substituted Acetophenone Derivatives Revealed by Through-Space 1H–19F and 13C–19F Spin–Spin Couplings
Source: J Org Chem. 2021 Mar 1;86(6):4638–45. doi: 10.1021/acs.joc.1c00051 (PMC8154564; doi:10.1021/acs.joc.1c00051)

Supporting Information

Conformational Preference of 2'-Fluoro-Substituted Acetophenone Derivatives Revealed by Through-Space ^{1}H–^{19}F and ^{13}C–^{19}F Spin–Spin Couplings

Chinatsu Otake,[1] Takuya Namba,[1] Hidetsugu Tabata,[2] Kosho Makino,[1] Kiriko Hirano,[3] Tetsuta Oshitari,[2] Hideaki Natsugari,[4] Takenori Kusumi,[5] and Hideyo Takahashi*[1]

[1]Faculty of Pharmaceutical Sciences, Tokyo University of Science, 2641Yamazaki, Noda-shi, Chiba 278-8510, Japan
[2]Faculty of Pharma Sciences, Teikyo University, 2-11-1 Kaga, Itabashi-ku, Tokyo 173-8605, Japan
[3]Bruker Japan K.K., 3-9 Moriya, Kanagawa-ku, Yokohama, Kanagawa 221-0022, Japan
[4]Graduate School of Pharmaceutical Science, The University of Tokyo, 7-3-1 Hongo, Bunkyo-ku, Tokyo 113-0033, Japan
[5]Department of Chemistry, Tokyo Institute of Technology, Meguro-ku, Tokyo 152-8551, Japan

hide-tak@rs.tus.ac.jp

Contents

1. **^{13}C NMR spectra of α-C of 1a and 1b**……………………………………………S2
2. **Spectra of through-space coupling of 1a-p, and 3-5**………………………….S3
3. **Crystal data of X-ray structure analysis of 1m and 1n**………………………S21
4. **DFT calculation study**…………………………………………………………….S23
5. **^{1}H and 13C {^{1}H} NMR spectra of 1a-p, and 3-5**………………………….S70

1. ^{13}C NMR spectra of α-C of 1a and 1b

^{13}C {^{1}H}NMR (100 MHz, $CDCl_3$, ppm) : α-C

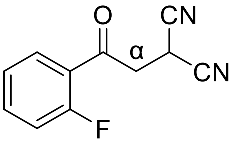

1a

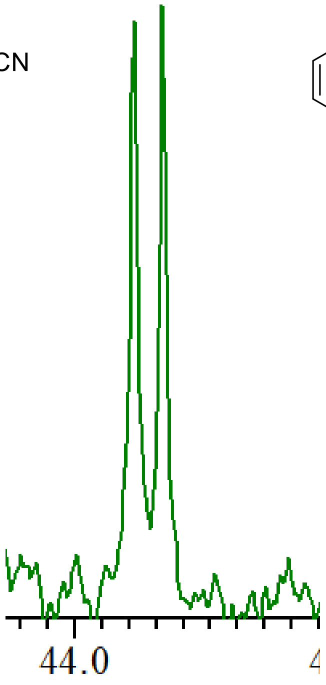

^{13}C{^{1}H} NMR (100 MHz, $CDCl_3$, ppm) : α-C

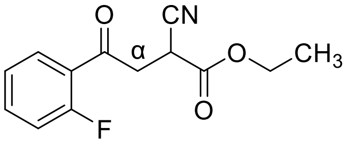

1b

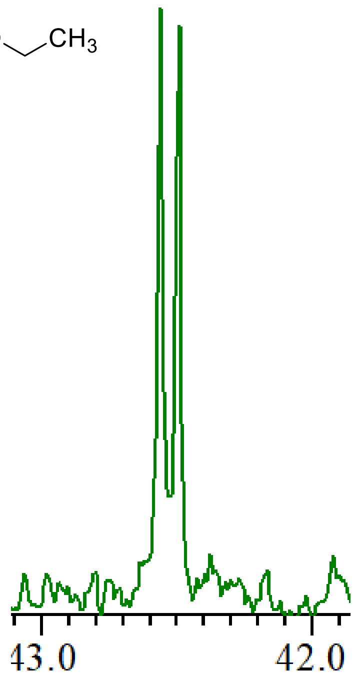

2. Spectra of through-space coupling of 1a-p, and 3-5.

2-[2-(2-Fluorophenyl)-2-oxoethyl]propanedinitrile (1a)

^{1}H NMR (400 MHz, $CDCl_3$, ppm) : α-H ^{13}C{^{1}H} NMR (100 MHz, $CDCl_3$, ppm) : α-C

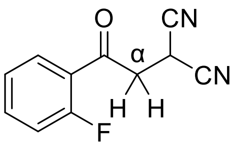

1a

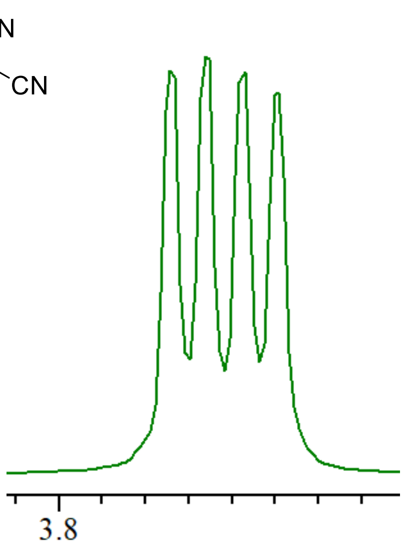

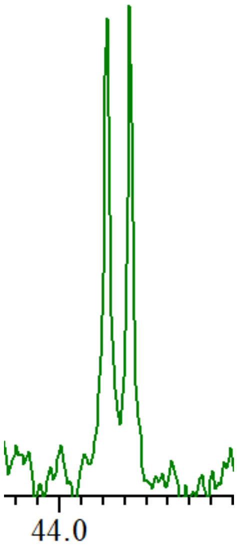

Ethyl 2-cyano-4-(2-fluorophenyl)-4-oxobutanoate (1b)

^{1}H NMR (400 MHz, $CDCl_3$, ppm) : α-H ^{13}C{^{1}H} NMR (100 MHz, $CDCl_3$, ppm) : α-C

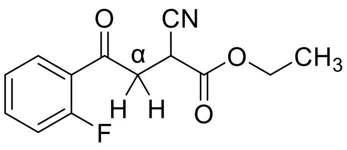

1b

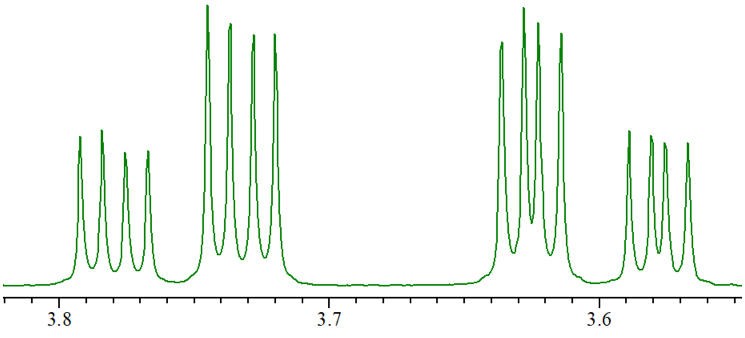

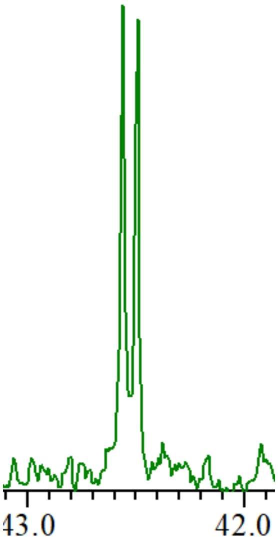

1-(2-Fluorophenyl)-1-butanone (1c)

^{1}H NMR (400 MHz, $CDCl_3$, ppm) : α-H

$^{13}C\{^1H\}$ NMR (100 MHz, $CDCl_3$, ppm) : α-C

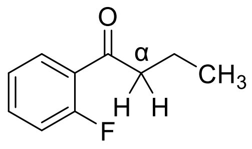

1c

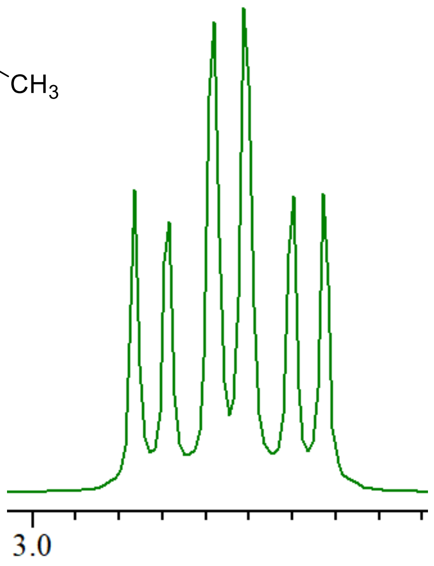

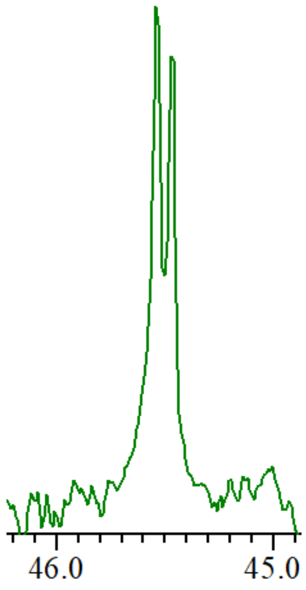

1-(2-Fluorophenyl)ethanone (1d)

^{1}H NMR (400 MHz, $CDCl_3$, ppm) : α-H ^{13}C{^{1}H} NMR (100 MHz, $CDCl_3$, ppm) : α-C

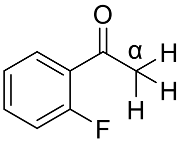

1d

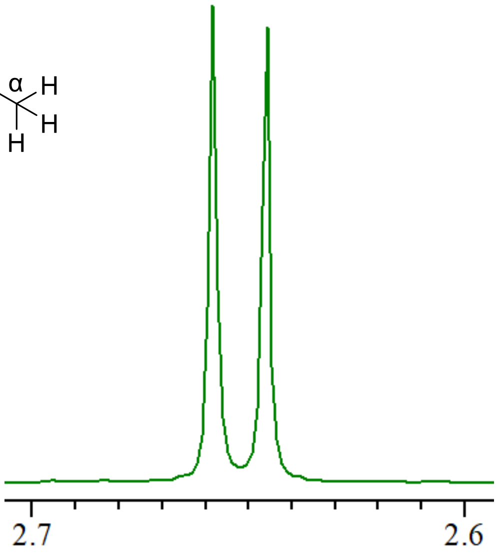

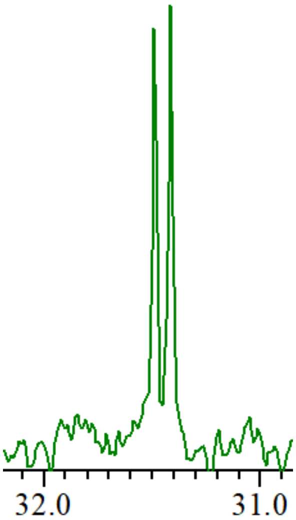

2-Bromo-1-(2-fluorophenyl)ethanone (1e)

^{1}H NMR (400 MHz, $CDCl_3$, ppm) : α-H

^{13}C{^{1}H} NMR (100 MHz, $CDCl_3$, ppm) : α-C

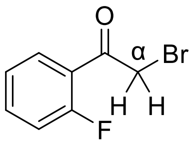

1e

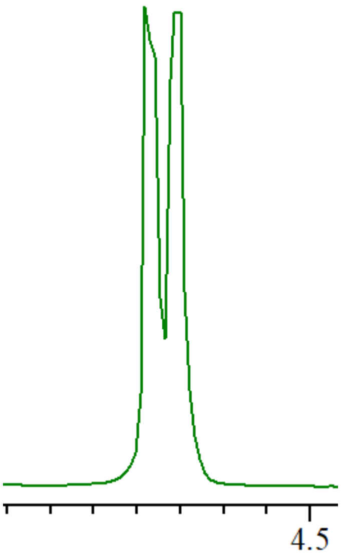

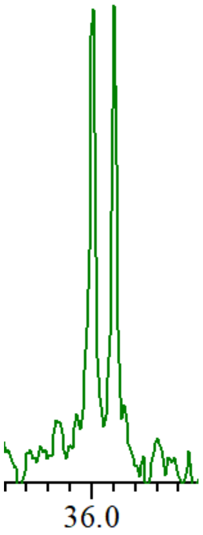

1-(2-Fluorophenyl) propanone (1f)

^{1}H NMR (400 MHz, $CDCl_3$, ppm) : α-H

$^{13}C\{^{1}H\}$ NMR (100 MHz, $CDCl_3$, ppm) : α-C

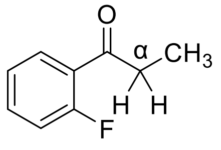

1f

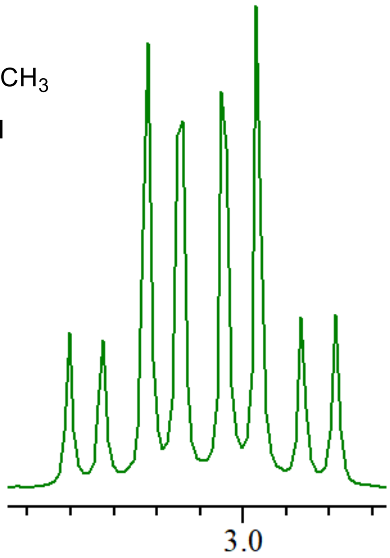

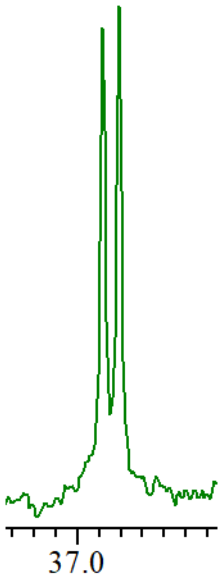

Ethyl 3-(2-fluorophenyl)-3-oxopropanoate (1g)

^{1}H NMR (400 MHz, $CDCl_3$, ppm) : α-H

^{13}C{^{1}H} NMR (100 MHz, $CDCl_3$, ppm) : α-C

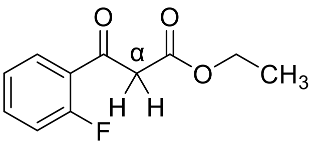

1g

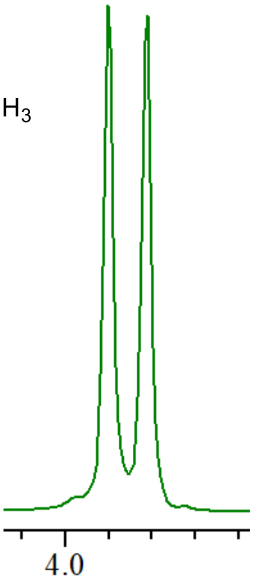

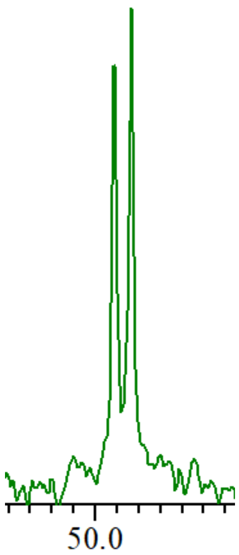

1-(4-Bromo-2-fluorophenyl)ethanone (1h)

^{1}H NMR (400 MHz, $CDCl_3$, ppm) : α-H

^{13}C{^{1}H} NMR (100 MHz, $CDCl_3$, ppm) : α-C

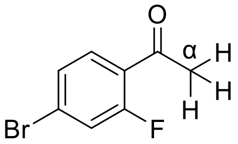

1h

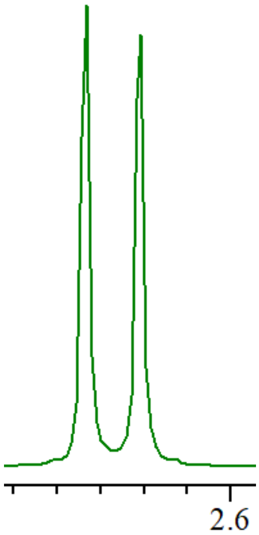

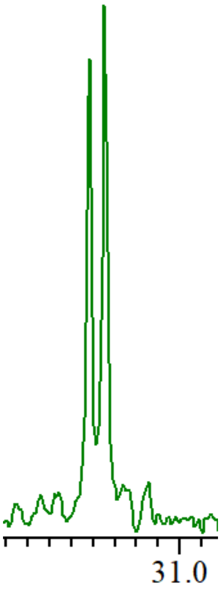

1-(5-Bromo-2-fluorophenyl)ethanone (1i)

^{1}H NMR (400 MHz, $CDCl_3$, ppm) : α-H

^{13}C{^{1}H} NMR (100 MHz, $CDCl_3$, ppm) : α-C

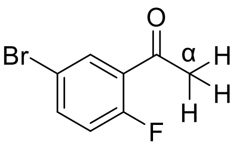

1i

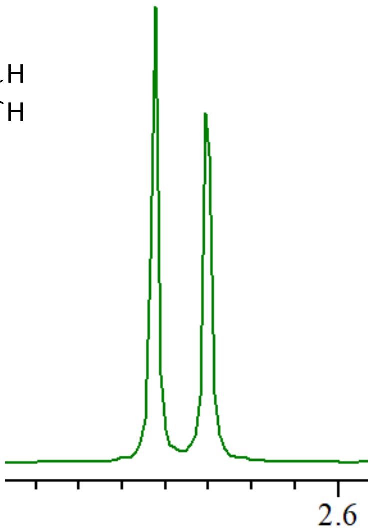

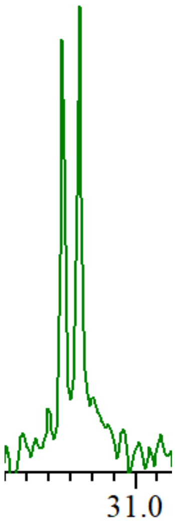

1-(2-Fluoro-4-hydroxyphenyl)ethanone (1j)

^{1}H NMR (400 MHz, $CDCl_3$, ppm) : α-H

$^{13}C\{^1H\}$ NMR (100 MHz, $CDCl_3$, ppm) : α-C

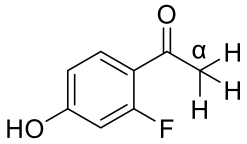

1j

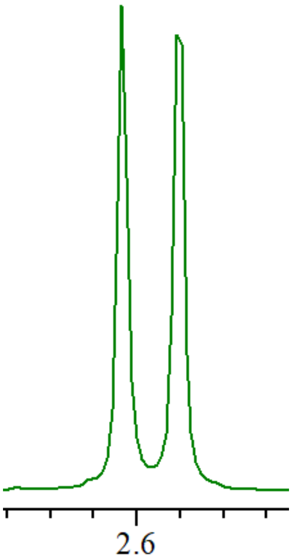

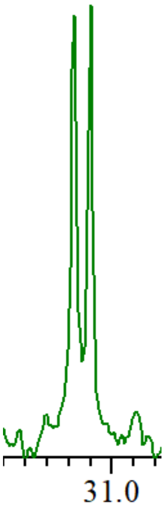

1-(2,4-Difluorophenyl)ethanone (1k)

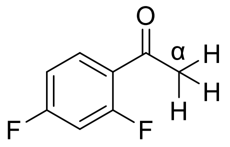

1k

^{1}H NMR (400 MHz, $CDCl_3$, ppm) : α-H

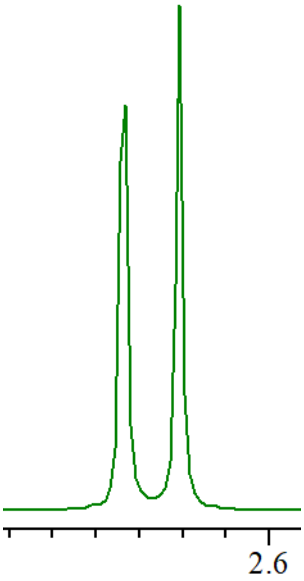

$^{13}C\{^{1}H\}$ NMR (100 MHz, $CDCl_3$, ppm) : α-C

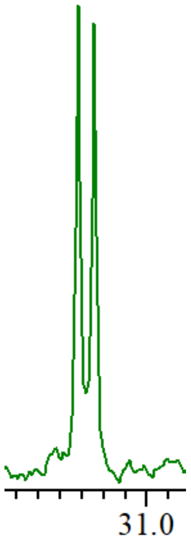

1-(2,4,5-Trifluorophenyl)ethanone (1l)

^{1}H NMR (400 MHz, $CDCl_3$, ppm) : α-H

^{13}C{^{1}H} NMR (100 MHz, $CDCl_3$, ppm) : α-C

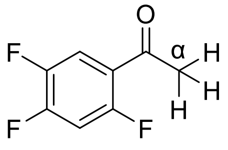

1l

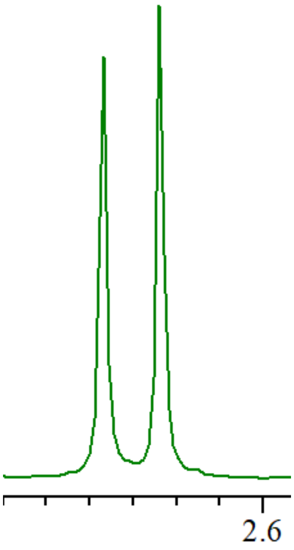

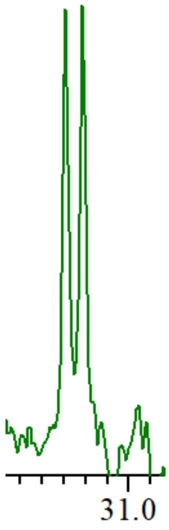

1-(2-Fluoro-5-nitrophenyl)ethanone (1m)

^{1}H NMR (400 MHz, $CDCl_3$, ppm) : α-H

^{13}C{^{1}H} NMR (100 MHz, $CDCl_3$, ppm) : α-C

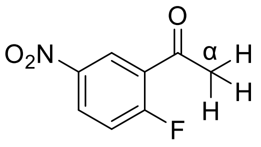

1m

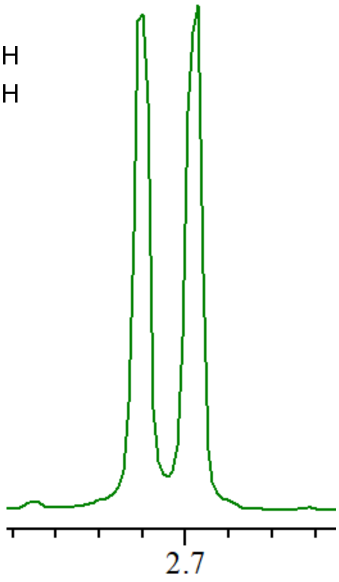

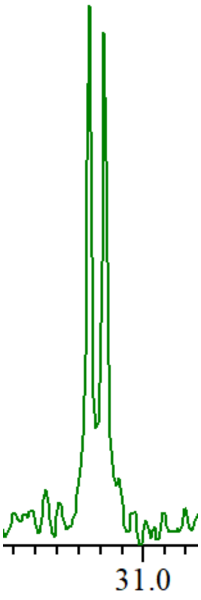

1-(2-Fluoro-4-methoxyphenyl)ethanone (1n)

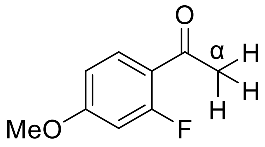

1n

^{1}H NMR (400 MHz, $CDCl_3$, ppm) : α-H

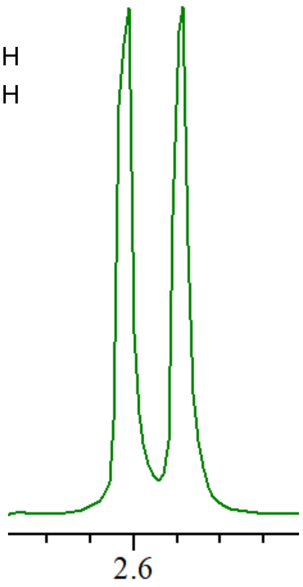

^{13}C{^{1}H} NMR (100 MHz, $CDCl_3$, ppm) : α-C

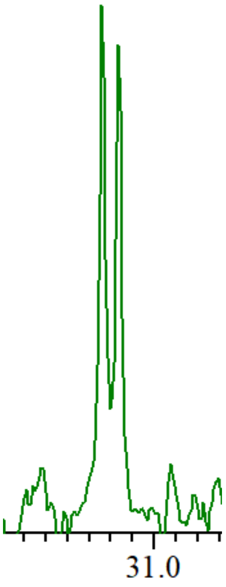

1-(2,4-Difluorophenyl)propanone (1o)

^{1}H NMR (400 MHz, $CDCl_3$, ppm) : α-H

^{13}C{^{1}H} NMR (100 MHz, $CDCl_3$, ppm) : α-C

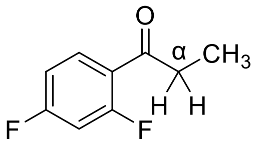

1o

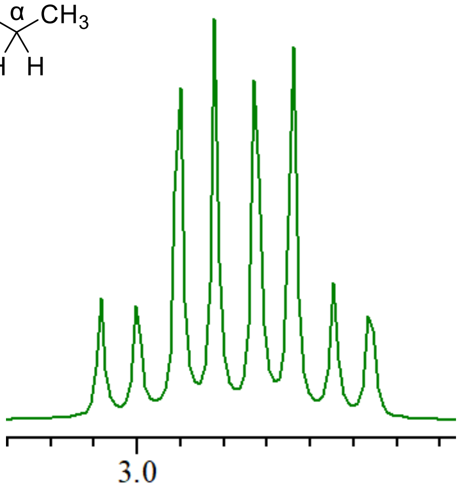

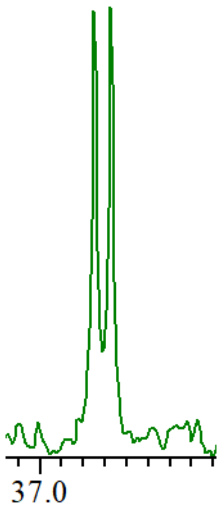

2-Chloro-1-(2,4-difluorophenyl)ethanone (1p)

^{1}H NMR (400 MHz, $CDCl_3$, ppm) : α-H

^{13}C{^{1}H} NMR (100 MHz, $CDCl_3$, ppm) : α-C

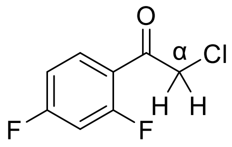

1q

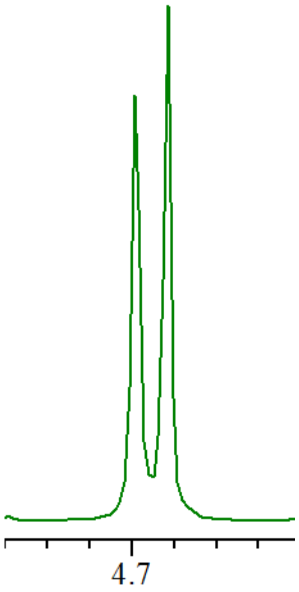

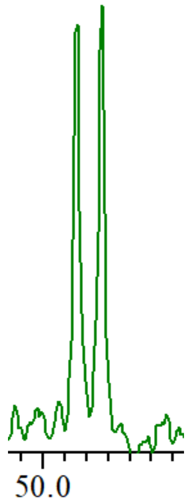

3’-Fluoroacetophenone (3)

^{1}H NMR (400 MHz, $CDCl_3$, ppm) : α-H

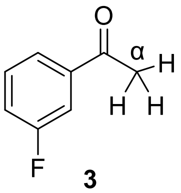

3

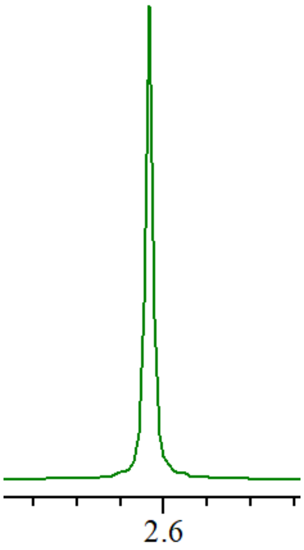

^{13}C{^{1}H} NMR (100 MHz, $CDCl_3$, ppm)

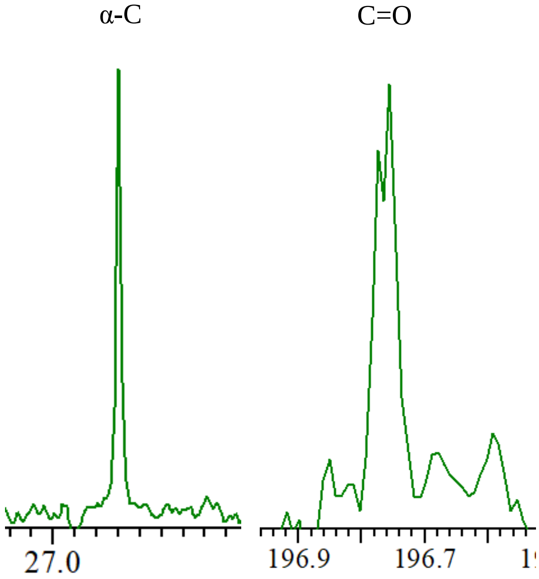

2’-fluorophenylacetone (4)

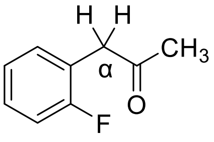

4

^{1}H NMR (400 MHz, $CDCl_3$, ppm) : α-H

^{13}C{^{1}H} NMR (100 MHz, $CDCl_3$, ppm)

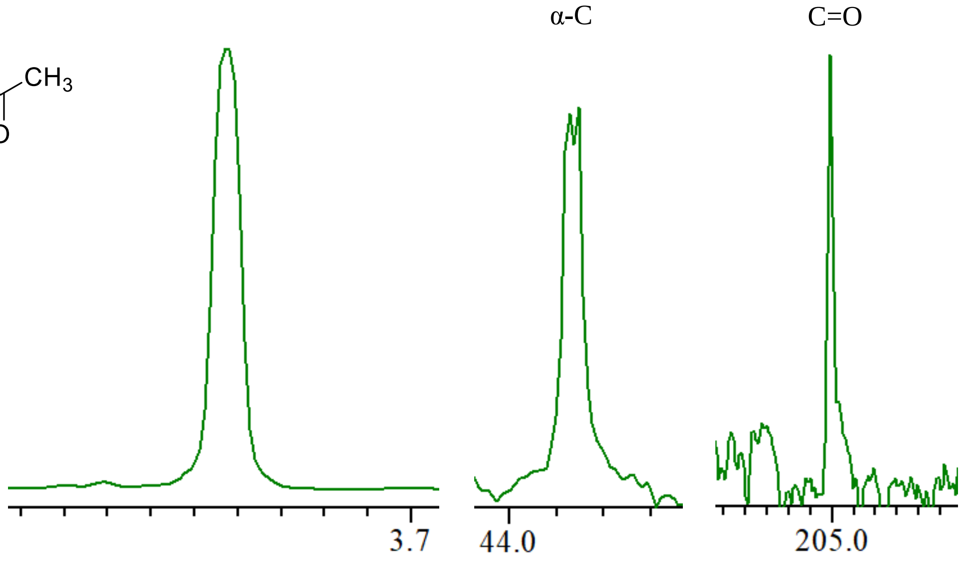

2-(2’-fluorophenyl)ethanol (5)

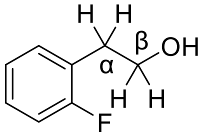

5

^{1}H NMR (400 MHz, $CDCl_3$, ppm)

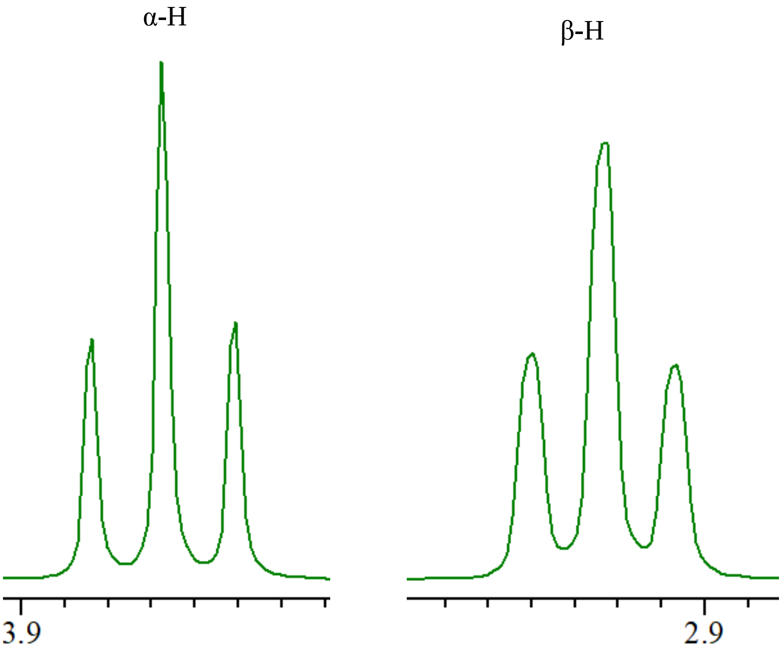

^{13}C{^{1}H} NMR (100 MHz, $CDCl_3$, ppm)

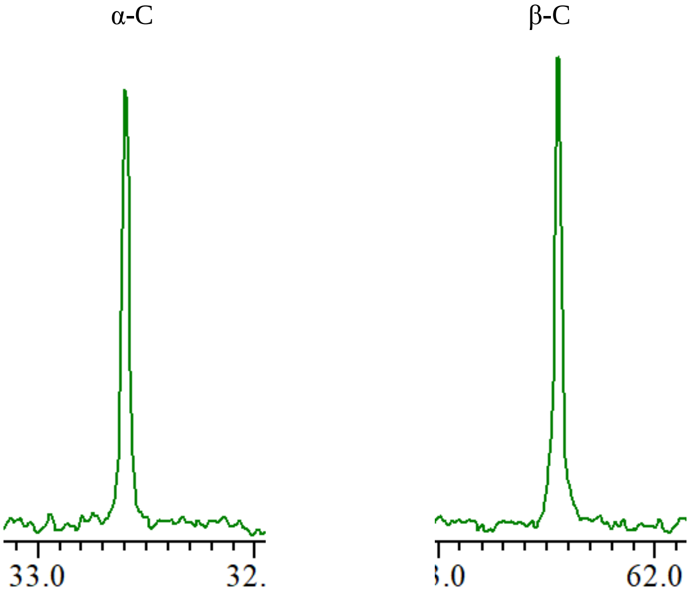

3. Crystal data of X-ray structure analysis of 1m and 1n

All measurements were made on a Rigaku Raxis Rapid imaging plate area detector with graphite monochromated Cu-Kα radiation. The data were collected at a temperature of –100 °C. The structure was solved by direct method SIR97 and expanded using Fourier techniques. The non-hydrogen atoms were refined anisotropically. All calculations were performed using the Crystal Structure (Crystal Structure 4.2.2) crystallographic software package except for refinement, which was performed using SHELXL97. Typical crystal data and ORTEP diagrams are as follows.

Crystals of **1m** and **1n** were obtained by dissolving the compounds in dichloromethane/hexane and allowing the solvent to slowly evaporate at room temperature.

Crystal data of 1m (CCDC 2044642)

$C_8H_6NO_3F$: mp 48–51 °C, *M*r = 183.14, CuKα (λ = 1.54187 Å), monoclinic, $P2_1/c$, colorless prism 0.300 × 0.200 × 0.150 mm, crystal dimensions a = 6.3839(2) Å, b =11.0189(3) Å, c = 11.3004(4) Å, α = 90°, β = 97.2472)°, γ = 90°, T = 173 K, Z = 4, V = 788.56(4) Å^3, D_{calc} = 1.542 g/cm^3, μ CuKα = 11.725 cm^{-1}, F_{000} = 376.00, GOF = 1.070, R_{int} = 0.0314, R_1 = 0.0339, wR_2 = 0.0743.

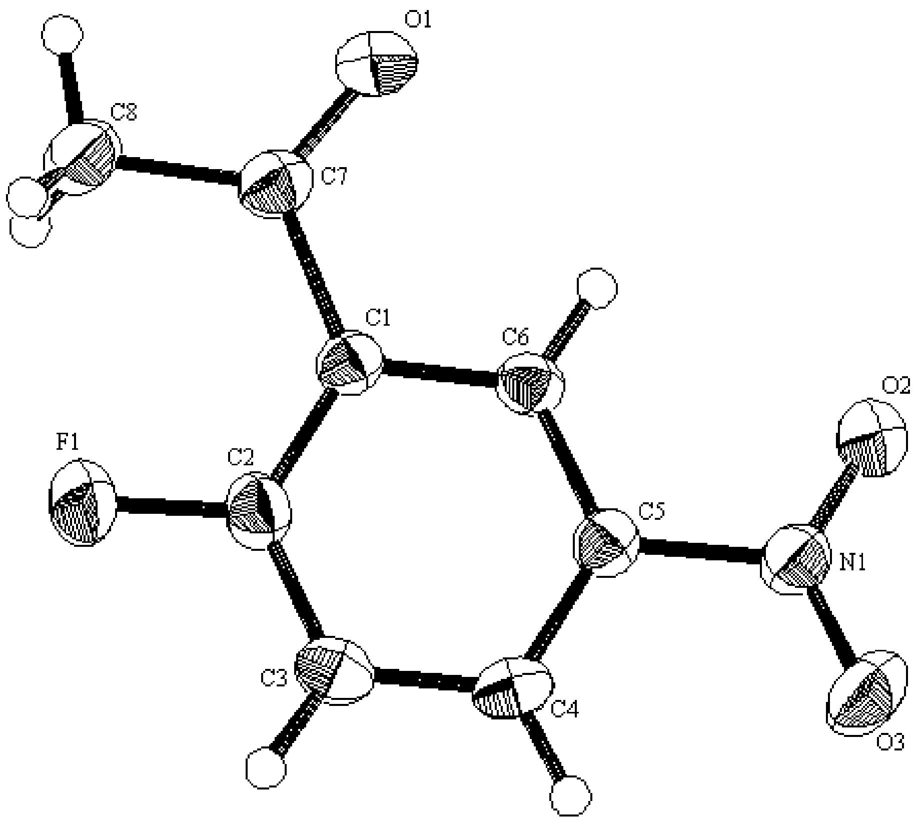

ORTEP view
Ellipsoid contour probability level = 50 %

Crystal data of 1n (CCDC 2044643)

$C_9H_9O_2F$: mp 49–50 °C, *M*r = 168.17, CuKα (λ = 1.54187 Å), monoclinic, *P*2_1/c, colorless prism 0.100 × 0.100 × 0.100mm, crystal dimensions *a* = 7.05142 Å, *b* = 10.72810 Å, *c* = 11.02417 Å, α = 90°, β = 106.02300°, γ = 90°, *T* = 173 K, *Z* = 4, *V* = 801.56159 Å^3, D_{calc} = 1.393 g/cm^3, μ CuKα = 9.605 cm^{-1}, F_{000} = 352.00, GOF = 1.246, R_{int} = 0.0502, R_1 = 0.0481, wR_2 = 0.1019.

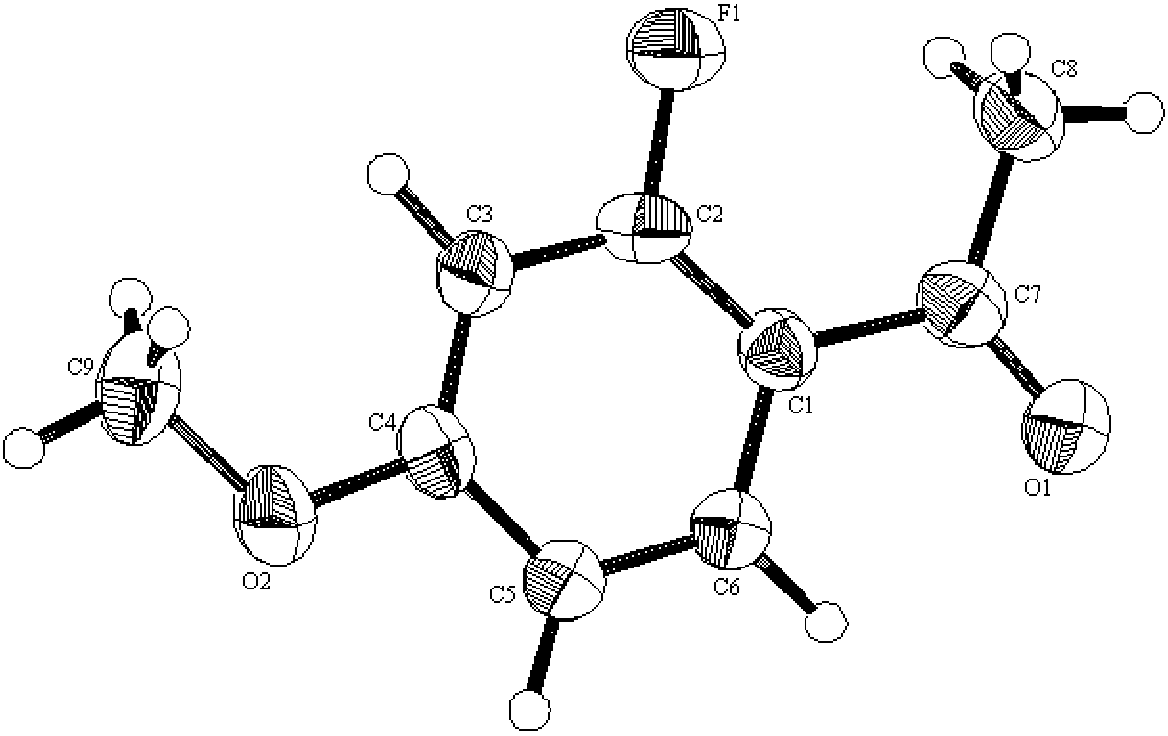

ORTEP view

Ellipsoid contour probability level = 50 %

4. DFT calculation study

DFT/HF calculations were performed with Gaussian 09 (**note S1**).

Note S1: Full reference

Gaussian 09, Revision C.01, M. J. Frisch, G. W. Trucks, H. B. Schlegel, G. E. Scuseria, M. A. Robb, J. R. Cheeseman, G. Scalmani, V. Barone, G. A. Petersson, H. Nakatsuji, X. Li, M. Caricato, A. Marenich, J. Bloino, B. G. Janesko, R. Gomperts, B. Mennucci, H. P. Hratchian, J. V. Ortiz, A. F. Izmaylov, J. L. Sonnenberg, D. Williams-Young, F. Ding, F. Lipparini, F. Egidi, J. Goings, B. Peng, A. Petrone, T. Henderson, D. Ranasinghe, V. G. Zakrzewski, J. Gao, N. Rega, G. Zheng, W. Liang, M. Hada, M. Ehara, K. Toyota, R. Fukuda, J. Hasegawa, M. Ishida, T. Nakajima, Y. Honda, O. Kitao, H. Nakai, T. Vreven, K. Throssell, J. A. Montgomery, Jr., J. E. Peralta, F. Ogliaro, M. Bearpark, J. J. Heyd, E. Brothers, K. N. Kudin, V. N. Staroverov, T. Keith, R. Kobayashi, J. Normand, K. Raghavachari, A. Rendell, J. C. Burant, S. S. Iyengar, J. Tomasi, M. Cossi, J. M. Millam, M. Klene, C. Adamo, R. Cammi, J. W. Ochterski, R. L. Martin, K. Morokuma, O. Farkas, J. B. Foresman, and D. J. Fox, Gaussian, Inc., Wallingford CT, 2016.

The results of the energies in the structurally optimized structures at the HF/6-31g(d) level and the energy calculations at the B3LYP/6-31G(d) level for all the generated conformations are shown in tables. The graphics of potential energies defined by the dihedral angle between Ph and CO in **1d**, **h-n** are also shown. Coordinates of the optimized structures of B3LYP/6-31G(d) for all *cis*/*trans* isomers of **1a-p** and mPW1PW91/6-311G(d, p) for all *trans* isomers of **1a-p** below. Optimized geometries were verified by frequency calculations at the mPW1PW91/6-311G(d, p) level as minima (zero imaginary frequencies). *Trans*/*cis* ratios were calculated by Sum of electronic and thermal Free Energies.

2-[2-(2-Fluorophenyl)-2-oxoethyl]propanedinitrile (1a)

Table S1 Energy of Conformers on HF/6-31G(d) and B3LYP//6-31G(d) level

conformer #	Energy (au) HF/6-31G(d)	B3LYP/6-31G(d)	Structure	conformer #	Energy (au) HF/6-31G(d)	B3LYP/6-31G(d)	Structure
1	-703.811	-707.898	trans	21	-703.805	-707.893	trans
2	-703.811	-707.898	trans	22	-703.805	-707.893	trans
3	-703.811	-707.898	trans	23	-703.805	-707.893	trans
4	-703.811	-707.898	trans	24	-703.805	-707.893	trans
5	-703.811	-707.898	trans	25	-703.808	-707.895	trans
6	-703.811	-707.898	trans	26	-703.808	-707.895	trans
7	-703.811	-707.898	trans	27	-703.808	-707.895	trans
8	-703.811	-707.898	trans	28	-703.808	-707.895	trans
9	-703.811	-707.898	trans	29	-703.808	-707.895	trans
10	-703.808	-707.902	trans	30	-703.808	-707.895	trans
11	-703.808	-707.902	trans	31	-703.804	-707.891	cis
12	-703.811	-707.905	trans	32	-703.804	-707.891	cis
13	-703.811	-707.905	trans	33	-703.804	-707.891	cis
14	-703.811	-707.905	trans	34	-703.804	-707.891	cis

15	-703.811	-707.905	trans	35	-703.804	-707.891	cis
16	-703.805	-707.899	trans	36	-703.804	-707.891	cis
17	-703.805	-707.899	trans	37	-703.8	-707.888	cis
18	-703.805	-707.899	trans	38	-703.8	-707.888	cis
19	-703.805	-707.893	trans	39	-703.8	-707.888	cis
20	-703.805	-707.893	trans	40	-703.8	-707.888	cis

Cis isomer

SCF Energy= -707.907109159 (Hartree)

Zero-point correction= 0.157117 (Hartree/Particle)

Thermal correction to Energy= 0.170259

Thermal correction to Enthalpy= 0.171203

Thermal correction to Gibbs Free Energy= 0.115281

Sum of electronic and zero-point Energies= -707.749993

Sum of electronic and thermal Energies= -707.736850

Sum of electronic and thermal Enthalpies= -707.735906

Sum of electronic and thermal Free Energies= -707.791829

Center Number	Atomic Number	Atomic Type	Coordinates (Angstroms) X	Y	Z
1	6	0	2.487833	-1.197808	1.256397
2	6	0	3.546407	-0.743942	0.476783
3	6	0	3.317832	0.153876	-0.554947
4	6	0	2.030167	0.594968	-0.798483
5	6	0	0.937401	0.161002	-0.036474
6	6	0	1.204958	-0.749467	0.996063
7	6	0	-0.438349	0.648162	-0.298532
8	6	0	-1.565918	0.052336	0.536892
9	8	0	-0.697920	1.496175	-1.123250
10	9	0	1.859192	1.454731	-1.800772
11	6	0	-2.924291	0.602068	0.066296
12	6	0	-3.311821	0.061204	-1.237898
13	7	0	-3.613306	-0.378289	-2.256238
14	6	0	-3.969482	0.326910	1.051191
15	7	0	-4.773542	0.117470	1.845758
16	1	0	2.662463	-1.898206	2.062462
17	1	0	4.554372	-1.089348	0.669997
18	1	0	4.120957	0.522438	-1.179553
19	1	0	0.393549	-1.113053	1.612218
20	1	0	-1.561015	-1.037641	0.486888
21	1	0	-1.422119	0.328858	1.584377
22	1	0	-2.837468	1.687457	-0.050378

Trans isomer
SCF Energy= -707.911765814 (Hartree)
Zero-point correction= 0.157165 (Hartree/Particle)
Thermal correction to Energy= 0.170229
Thermal correction to Enthalpy= 0.171173
Thermal correction to Gibbs Free Energy= 0.115842
Sum of electronic and zero-point Energies= -707.754600
Sum of electronic and thermal Energies= -707.741537
Sum of electronic and thermal Enthalpies= -707.740593
Sum of electronic and thermal Free Energies= -707.795924

Center Number	Atomic Number	Atomic Type	Coordinates (Angstroms) X	Y	Z
1	6	0	2.888304	-1.132987	1.477199
2	6	0	3.628350	-0.443944	0.519024
3	6	0	2.985601	0.271495	-0.479850
4	6	0	1.603797	0.290473	-0.506201
5	6	0	0.826967	-0.383434	0.435885
6	6	0	1.507838	-1.100008	1.429506
7	6	0	-0.659630	-0.408770	0.473520
8	6	0	-1.456164	0.407387	-0.525654
9	8	0	-1.243574	-1.082275	1.296369
10	9	0	1.014582	0.987956	-1.491592
11	6	0	-2.963548	0.295355	-0.232646
12	6	0	-3.752482	0.791188	-1.359536
13	7	0	-4.356845	1.167271	-2.262284
14	6	0	-3.338186	1.007095	0.990732
15	7	0	-3.624768	1.577966	1.946300
16	1	0	3.390788	-1.691294	2.256276
17	1	0	4.710727	-0.462825	0.547351
18	1	0	3.533242	0.813815	-1.239243
19	1	0	0.909299	-1.626206	2.161779
20	1	0	-1.256416	0.031692	-1.531675
21	1	0	-1.135822	1.449768	-0.518266
22	1	0	-3.212259	-0.759717	-0.078395

Ethyl 2-cyano-4-(2-fluorophenyl)-4-oxobutanoate (1b)
Table S2

conformer #	Energy (au) HF/6-31G(d)	B3LYP/6-31G(d)	Structure	conformer #	Energy (au) HF/6-31G(d)	B3LYP/6-31G(d)	Structure
1	-877.771	-882.86	trans	45	-877.768	-882.858	trans
2	-877.77	-882.86	trans	46	-877.767	-882.858	trans
3	-877.77	-882.86	trans	47	-877.767	-882.858	trans
4	-877.77	-882.86	trans	48	-877.763	-882.854	trans
5	-877.77	-882.86	trans	49	-877.763	-882.853	trans
6	-877.77	-882.86	trans	50	-877.763	-882.853	trans
7	-877.768	-882.858	trans	51	-877.763	-882.853	trans
8	-877.768	-882.858	trans	52	-877.763	-882.854	trans
9	-877.768	-882.858	trans	53	-877.766	-882.856	trans
10	-877.766	-882.856	trans	54	-877.763	-882.854	trans
11	-877.766	-882.856	trans	55	-877.766	-882.856	trans
12	-877.767	-882.858	trans	56	-877.766	-882.856	trans
13	-877.767	-882.858	trans	57	-877.763	-882.854	trans
14	-877.767	-882.858	trans	58	-877.762	-882.853	trans
15	-877.767	-882.858	trans	59	-877.762	-882.853	trans
16	-877.767	-882.858	trans	60	-877.77	-882.86	trans
17	-877.767	-882.858	trans	61	-877.762	-882.853	trans
18	-877.766	-882.856	trans	62	-877.762	-882.853	trans
19	-877.769	-882.859	trans	63	-877.766	-882.856	trans
20	-877.769	-882.859	trans	64	-877.768	-882.858	trans
21	-877.769	-882.859	trans	65	-877.762	-882.853	trans
22	-877.766	-882.856	trans	66	-877.766	-882.856	trans
23	-877.767	-882.857	trans	67	-877.762	-882.853	trans
24	-877.767	-882.857	trans	68	-877.767	-882.857	trans
25	-877.766	-882.856	trans	69	-877.764	-882.854	cis
26	-877.766	-882.856	trans	70	-877.768	-882.859	trans
27	-877.768	-882.859	trans	71	-877.762	-882.852	trans
28	-877.768	-882.858	trans	72	-877.762	-882.852	trans
29	-877.768	-882.858	trans	73	-877.766	-882.856	trans
30	-877.768	-882.858	trans	74	-877.766	-882.856	trans
31	-877.767	-882.857	trans	75	-877.761	-882.852	trans
32	-877.766	-882.856	trans	76	-877.761	-882.852	trans

33	-877.766	-882.856	trans	77	-877.761	-882.852	trans
34	-877.766	-882.856	trans	78	-877.761	-882.852	trans
35	-877.766	-882.856	trans	79	-877.761	-882.852	trans
36	-877.763	-882.853	trans	80	-877.761	-882.852	cis
37	-877.763	-882.853	trans	81	-877.756	-882.847	trans
38	-877.763	-882.853	trans	82	-877.759	-882.849	cis
39	-877.763	-882.853	trans	83	-877.759	-882.849	cis
40	-877.762	-882.853	trans	84	-877.761	-882.851	cis
41	-877.762	-882.853	trans	85	-877.762	-882.852	cis
42	-877.762	-882.853	trans	86	-877.758	-882.849	cis
43	-877.762	-882.853	trans	87	-877.759	-882.849	cis
44	-877.762	-882.853	trans				

Cis isomer
SCF Energy= -882.878890002(Hartree)
Zero-point correction= 0.230170 (Hartree/Particle)
Thermal correction to Energy= 0.247279
Thermal correction to Enthalpy= 0.248223
Thermal correction to Gibbs Free Energy= 0.181920
Sum of electronic and zero-point Energies= -882.648720
Sum of electronic and thermal Energies= -882.631611
Sum of electronic and thermal Enthalpies= -882.630667
Sum of electronic and thermal Free Energies= -882.696970

Center Number	Atomic Number	Atomic Type	Coordinates (Angstroms) X	Y	Z
1	6	0	-4.390561	0.497724	0.717471
2	6	0	-5.073866	-0.623301	0.259105
3	6	0	-4.423407	-1.560558	-0.529111
4	6	0	-3.093016	-1.368967	-0.854647
5	6	0	-2.369493	-0.253918	-0.412977
6	6	0	-3.058731	0.671629	0.383355
7	6	0	-0.940898	-0.051844	-0.769884
8	6	0	-0.241525	1.174185	-0.201470
9	8	0	-0.331627	-0.808953	-1.493782
10	9	0	-2.513519	-2.299091	-1.612974
11	6	0	1.228329	1.180602	-0.607979
12	6	0	1.844778	2.476153	-0.356435
13	7	0	2.321887	3.503238	-0.149811
14	6	0	2.016444	0.092541	0.133971
15	8	0	1.609698	-0.475202	1.110126
16	8	0	3.184956	-0.107580	-0.453496
17	6	0	4.068542	-1.084252	0.149109
18	6	0	4.895385	-0.462817	1.249582
19	1	0	-4.893657	1.231564	1.333427
20	1	0	-6.115648	-0.771465	0.515010
21	1	0	-4.927545	-2.443636	-0.899489
22	1	0	-2.540928	1.547866	0.749388
23	1	0	-0.322636	1.180229	0.886982
24	1	0	-0.736235	2.075168	-0.572415
25	1	0	1.323175	0.965445	-1.675190
26	1	0	4.687272	-1.425202	-0.678436
27	1	0	3.464634	-1.913760	0.515425
28	1	0	5.459175	0.393065	0.875564
29	1	0	4.264588	-0.137884	2.077671
30	1	0	5.604230	-1.200779	1.630610

Trans isomer
SCF Energy= -882.883587245 (Hartree)
Zero-point correction= 0.230016 (Hartree/Particle)
Thermal correction to Energy= 0.247158
Thermal correction to Enthalpy= 0.248102
Thermal correction to Gibbs Free Energy= 0.181986
Sum of electronic and zero-point Energies= -882.653571
Sum of electronic and thermal Energies= -882.636430
Sum of electronic and thermal Enthalpies= -882.635485
Sum of electronic and thermal Free Energies= -882.701601

Center Number	Atomic Number	Atomic Type	Coordinates (Angstroms) X	Y	Z
1	6	0	-4.210890	0.240519	-1.885754
2	6	0	-5.064038	-0.362116	-0.964306
3	6	0	-4.605407	-0.684740	0.303644
4	6	0	-3.293217	-0.401186	0.635117
5	6	0	-2.407235	0.200309	-0.258201
6	6	0	-2.903983	0.514956	-1.530178
7	6	0	-0.982042	0.536279	0.022121
8	6	0	-0.366134	0.174234	1.356317
9	8	0	-0.315719	1.104386	-0.818687
10	9	0	-2.889639	-0.726332	1.874198
11	6	0	1.111669	0.551108	1.381392
12	6	0	1.654702	0.482751	2.731484
13	7	0	2.075604	0.423779	3.801301
14	6	0	1.934638	-0.359487	0.460619
15	8	0	1.546424	-1.415270	0.042480
16	8	0	3.117319	0.179030	0.214645
17	6	0	4.012793	-0.589632	-0.622424
18	6	0	5.295238	0.189508	-0.747281
19	1	0	-4.567865	0.491921	-2.876091
20	1	0	-6.090127	-0.582442	-1.231561
21	1	0	-5.243602	-1.151843	1.042113
22	1	0	-2.220637	0.982254	-2.227407
23	1	0	-0.488410	-0.894146	1.539084
24	1	0	-0.908916	0.689693	2.151456
25	1	0	1.247348	1.573116	1.019278
26	1	0	4.165153	-1.564177	-0.156227
27	1	0	3.527587	-0.744411	-1.587251
28	1	0	5.754893	0.342621	0.230110
29	1	0	5.117441	1.162677	-1.207019
30	1	0	5.996950	-0.363359	-1.374172

1-(2-Fluorophenyl)-1-butanone (1c)

Table S3

conformer #	Energy (au) HF/6-31G(d)	B3LYP/6-31G(d)	Structure
1	-559.395	-562.753	trans
2	-559.394	-562.753	trans
3	-559.394	-562.753	trans
4	-559.393	-562.751	trans
5	-559.394	-562.753	trans
6	-559.394	-562.753	trans
7	-559.394	-562.753	trans
8	-559.394	-562.753	trans
9	-559.389	-562.747	cis
10	-559.388	-562.746	cis
11	-559.387	-562.745	cis
12	-559.387	-562.744	cis

Cis isomer
SCF Energy= -562.762941278 (Hartree)
Zero-point correction= 0.187508 (Hartree/Particle)
Thermal correction to Energy= 0.198852
Thermal correction to Enthalpy= 0.199796
Thermal correction to Gibbs Free Energy= 0.148036
Sum of electronic and zero-point Energies= -562.575433
Sum of electronic and thermal Energies= -562.564089
Sum of electronic and thermal Enthalpies= -562.563145
Sum of electronic and thermal Free Energies= -562.614905

Center Number	Atomic Number	Atomic Type	Coordinates (Angstroms) X	Y	Z
1	6	0	-2.969354	0.794785	-0.161960
2	6	0	-3.777640	-0.042182	0.598845
3	6	0	-3.213065	-1.089153	1.311424
4	6	0	-1.845092	-1.290802	1.257548
5	6	0	-0.995637	-0.471576	0.504025
6	6	0	-1.602315	0.575953	-0.202567
7	6	0	0.483407	-0.693512	0.448727
8	6	0	1.309578	0.254168	-0.398543
9	8	0	1.013058	-1.598029	1.057430
10	9	0	-1.359943	-2.314064	1.964130
11	6	0	2.796193	-0.064149	-0.381147
12	6	0	3.603518	0.900635	-1.238809
13	1	0	-3.402489	1.614263	-0.721037
14	1	0	-4.848139	0.117274	0.639732
15	1	0	-3.813474	-1.759108	1.913361
16	1	0	-0.986801	1.235657	-0.798961
17	1	0	1.135945	1.278120	-0.046983
18	1	0	0.927126	0.227388	-1.425793
19	1	0	3.155390	-0.040516	0.650918
20	1	0	2.946731	-1.090262	-0.726347
21	1	0	3.282270	0.869302	-2.283772
22	1	0	3.493361	1.931428	-0.890298
23	1	0	4.667073	0.654382	-1.212223

Trans isomer

SCF Energy= -562.767306447 (Hartree)

Zero-point correction= 0.187450 (Hartree/Particle)

Thermal correction to Energy= 0.198754

Thermal correction to Enthalpy= 0.199698

Thermal correction to Gibbs Free Energy= 0.148770

Sum of electronic and zero-point Energies= -562.579856

Sum of electronic and thermal Energies= -562.568552

Sum of electronic and thermal Enthalpies= -562.567608

Sum of electronic and thermal Free Energies= -562.618537

Center Number	Atomic Number	Atomic Type	Coordinates (Angstroms) X	Y	Z
1	6	0	-3.485021	0.920032	0.387368
2	6	0	-3.778590	-0.381053	0.787261
3	6	0	-2.770432	-1.330104	0.855081
4	6	0	-1.477772	-0.965671	0.521369
5	6	0	-1.141074	0.325067	0.116328
6	6	0	-2.184845	1.257536	0.059442
7	6	0	0.230051	0.807041	-0.263023
8	6	0	1.403889	-0.138642	-0.226434
9	8	0	0.372617	1.965614	-0.597355
10	9	0	-0.537808	-1.923455	0.604002
11	6	0	2.717636	0.504995	-0.638269
12	6	0	3.879821	-0.477579	-0.589722
13	1	0	-4.269233	1.664384	0.332986
14	1	0	-4.792558	-0.659546	1.047066
15	1	0	-2.964011	-2.349808	1.161805
16	1	0	-1.928928	2.261449	-0.254499
17	1	0	1.170023	-0.997055	-0.866029
18	1	0	1.472129	-0.558242	0.783480
19	1	0	2.920109	1.358369	0.014309
20	1	0	2.616052	0.916704	-1.645879
21	1	0	3.713202	-1.324807	-1.260891
22	1	0	4.020705	-0.878136	0.418113
23	1	0	4.813838	0.002907	-0.888609

1-(2-Fluorophenyl)ethanone (1d)

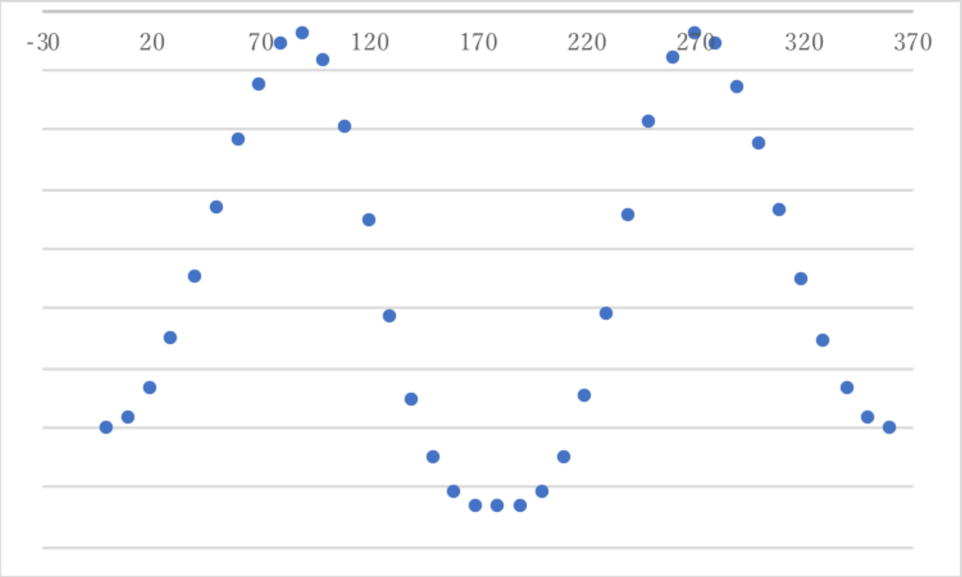

Figure S1

Table S4

conformer #	Angle	energy(au)	conformer #	Angle	energy(au)
1	0.1979	-477.839	20	190.1979	-477.84
2	10.1979	-477.839	21	200.1979	-477.84
3	20.1979	-477.838	22	210.1979	-477.84
4	30.1979	-477.838	23	220.1979	-477.839
5	40.1979	-477.836	24	230.1979	-477.837
6	50.1979	-477.835	25	240.1979	-477.835
7	60.1979	-477.834	26	250.1979	-477.834
8	70.1979	-477.833	27	260.1979	-477.833
9	80.1979	-477.833	28	270.1979	-477.832
10	90.1979	-477.832	29	280.1979	-477.833
11	100.1979	-477.833	30	290.1979	-477.833
12	110.1979	-477.834	31	300.1979	-477.834
13	120.1979	-477.836	32	310.1979	-477.835
14	130.1979	-477.837	33	320.1979	-477.837
15	140.1979	-477.839	34	330.1979	-477.838
16	150.1979	-477.84	35	340.1979	-477.838
17	160.1979	-477.84	36	350.1979	-477.839
18	170.1979	-477.84	37	360.1979	-477.839
19	180.1979	-477.84			

Cis isomer
SCF Energy= -484.133463761 (Hartree)
Zero-point correction= 0.130300 (Hartree/Particle)
Thermal correction to Energy= 0.138053
Thermal correction to Enthalpy= 0.138997
Thermal correction to Gibbs Free Energy= 0.097764
Sum of electronic and zero-point Energies= -484.003164
Sum of electronic and thermal Energies= -483.995411
Sum of electronic and thermal Enthalpies= -483.994467
Sum of electronic and thermal Free Energies= -484.035700

Center Number	Atomic Number	Atomic Type	Coordinates (Angstroms) X	Y	Z
1	6	0	1.752129	-1.586908	-0.001487
2	6	0	2.567608	-0.460942	-0.000511
3	6	0	2.001730	0.805222	0.000846
4	6	0	0.624385	0.935811	0.000952
5	6	0	-0.232043	-0.171811	-0.000219
6	6	0	0.375596	-1.434739	-0.001209
7	6	0	-1.719933	-0.031559	-0.000987
8	6	0	-2.546972	-1.295121	0.004232
9	8	0	-2.260505	1.053198	-0.005941
10	1	0	-2.335294	-1.902281	0.887313
11	1	0	-2.338423	-1.907145	-0.876245
12	1	0	-3.597096	-1.012557	0.005111
13	9	0	0.136239	2.177463	0.002779
14	1	0	2.187069	-2.578033	-0.002472
15	1	0	2.608921	1.701217	0.001852
16	1	0	-0.247933	-2.318752	-0.002028
17	1	0	3.645644	-0.564912	-0.000714

Trans isomer
SCF Energy= -484.137700075 (Hartree)
Zero-point correction= 0.130405 (Hartree/Particle)
Thermal correction to Energy= 0.138957
Thermal correction to Enthalpy= 0.139901
Thermal correction to Gibbs Free Energy= 0.096328
Sum of electronic and zero-point Energies= -484.007296
Sum of electronic and thermal Energies= -483.998743
Sum of electronic and thermal Enthalpies= -483.997799
Sum of electronic and thermal Free Energies= -484.041372

Center Number	Atomic Number	Atomic Type	Coordinates (Angstroms) X	Y	Z
1	6	0	-2.012541	-1.341446	-0.000001
2	6	0	-2.620671	-0.088524	0.000000
3	6	0	-1.845231	1.060706	-0.000025
4	6	0	-0.466578	0.942361	-0.000053
5	6	0	0.181122	-0.292114	-0.000057
6	6	0	-0.632976	-1.431712	-0.000028
7	6	0	1.665728	-0.504320	-0.000087
8	6	0	2.598839	0.673137	-0.000113
9	8	0	2.100676	-1.637696	0.000045
10	1	0	2.426311	1.301261	0.875710
11	1	0	2.426295	1.301246	-0.875944
12	1	0	3.621953	0.303694	-0.000115
13	9	0	0.243181	2.083706	-0.000072
14	1	0	-2.615635	-2.240399	0.000019
15	1	0	-2.286464	2.048877	-0.000022
16	1	0	-0.133688	-2.392331	-0.000027
17	1	0	-3.700402	-0.003387	0.000022

2-Bromo-1-(2-fluorophenyl)ethanone (1e)

Table S5

conformer #	Energy (au)		Structure
	HF/6-31G(d)	B3LYP/6-31G(d)	
1	-3050.63	-3055.23	trans
2	-3050.63	-3055.23	trans
3	-3050.63	-3055.23	trans
4	-3050.63	-3055.23	trans
5	-3050.63	-3055.23	trans
6	-3050.63	-3055.23	trans
7	-3050.62	-3055.22	cis
8	-3050.62	-3055.22	cis
9	-3050.62	-3055.22	cis

Cis isomer

SCF Energy= -3057.83450601 (Hartree)

Zero-point correction= 0.121521 (Hartree/Particle)

Thermal correction to Energy= 0.131184

Thermal correction to Enthalpy= 0.132128

Thermal correction to Gibbs Free Energy= 0.083861

Sum of electronic and zero-point Energies= -3057.712985

Sum of electronic and thermal Energies= -3057.703322

Sum of electronic and thermal Enthalpies= -3057.702378

Sum of electronic and thermal Free Energies= -3057.750645

Center Number	Atomic Number	Atomic Type	Coordinates (Angstroms) X	Y	Z
1	6	0	-1.682372	1.134577	-0.245633
2	6	0	-2.518775	0.156836	0.281657
3	6	0	-2.001394	-1.073873	0.657725
4	6	0	-0.651045	-1.320029	0.491725
5	6	0	0.222461	-0.362818	-0.036446
6	6	0	-0.330667	0.874249	-0.391908
7	6	0	1.662404	-0.662077	-0.251429
8	6	0	2.626205	0.492724	-0.469044
9	8	0	2.101212	-1.788630	-0.282461
10	9	0	-0.192857	-2.510329	0.881483
11	35	0	2.820114	1.557456	1.157910
12	1	0	-2.081864	2.096805	-0.538121
13	1	0	-3.576874	0.351429	0.405426
14	1	0	-2.625151	-1.850707	1.080223
15	1	0	0.307938	1.647774	-0.796688

16	1	0	2.315909	1.176520	-1.255108
17	1	0	3.604754	0.079993	-0.689312

Trans isomer

SCF Energy= -3057.83832654 (Hartree)

Zero-point correction= 0.121707 (Hartree/Particle)

Thermal correction to Energy= 0.131284

Thermal correction to Enthalpy= 0.132228

Thermal correction to Gibbs Free Energy= 0.084344

Sum of electronic and zero-point Energies= -3057.716619

Sum of electronic and thermal Energies= -3057.707042

Sum of electronic and thermal Enthalpies= -3057.706098

Sum of electronic and thermal Free Energies= -3057.753982

Center Number	Atomic Number	Atomic Type	Coordinates (Angstroms) X	Y	Z
1	6	0	-2.078745	0.863663	-0.727648
2	6	0	-2.554581	0.000699	0.256219
3	6	0	-1.666320	-0.719291	1.040383
4	6	0	-0.308309	-0.573643	0.825199
5	6	0	0.208198	0.275669	-0.152965
6	6	0	-0.716586	0.997588	-0.919041
7	6	0	1.653185	0.512752	-0.436811
8	6	0	2.722622	-0.399784	0.123509
9	8	0	1.994142	1.440103	-1.137898
10	9	0	0.515058	-1.280535	1.616364
11	35	0	2.647708	-2.127521	-0.789779
12	1	0	-2.770419	1.427506	-1.340073
13	1	0	-3.619589	-0.112074	0.416946
14	1	0	-2.004015	-1.391644	1.818037
15	1	0	-0.321872	1.665880	-1.673407
16	1	0	2.605372	-0.606791	1.181711
17	1	0	3.694051	0.027422	-0.100946

1-(2-Fluorophenyl)-1-propanone (1f)
Table S6

conformer #	Energy (au) HF/6-31G(d)	B3LYP/6-31G(d)	Structure
1	-520.361	-523.44	trans
2	-520.358	-523.438	trans
3	-520.358	-523.438	trans
4	-520.355	-523.433	cis
5	-520.355	-523.433	cis
6	-520.353	-523.431	cis

Cis isomer
SCF Energy= -523.448481152 (Hartree)
Zero-point correction= 0.158919 (Hartree/Particle)
Thermal correction to Energy= 0.168018
Thermal correction to Enthalpy= 0.168962
Thermal correction to Gibbs Free Energy= 0.124512
Sum of electronic and zero-point Energies= -523.289563
Sum of electronic and thermal Energies= -523.280463
Sum of electronic and thermal Enthalpies= -523.279519
Sum of electronic and thermal Free Energies= -523.323969

Center Number	Atomic Number	Atomic Type	Coordinates (Angstroms) X	Y	Z
1	6	0	-2.110009	0.392251	1.307070
2	6	0	-3.116217	0.599403	0.370418
3	6	0	-2.820701	0.558868	-0.983654
4	6	0	-1.521316	0.311557	-1.390446
5	6	0	-0.479003	0.097704	-0.480243
6	6	0	-0.815913	0.146148	0.879465
7	6	0	0.924792	-0.169381	-0.924063
8	6	0	1.975798	-0.388958	0.147685
9	8	0	1.224195	-0.211272	-2.097493
10	9	0	-1.297616	0.284418	-2.705884
11	6	0	3.356094	-0.650772	-0.426799
12	1	0	-2.333248	0.422436	2.365754
13	1	0	-4.132551	0.792844	0.690721
14	1	0	-3.580326	0.715807	-1.738527
15	1	0	-0.044120	-0.013027	1.620161
16	1	0	1.654938	-1.223844	0.779926
17	1	0	1.989372	0.488818	0.802715
18	1	0	4.078583	-0.802339	0.376821
19	1	0	3.692008	0.187825	-1.037925
20	1	0	3.355138	-1.538389	-1.060603

Trans isomer

SCF Energy= -523.452833465 (Hartree)

Zero-point correction= 0.158898 (Hartree/Particle)

Thermal correction to Energy= 0.168861

Thermal correction to Enthalpy= 0.169805

Thermal correction to Gibbs Free Energy= 0.122287

Sum of electronic and zero-point Energies= -523.293936

Sum of electronic and thermal Energies= -523.283972

Sum of electronic and thermal Enthalpies= -523.283028

Sum of electronic and thermal Free Energies= -523.330547

Center Number	Atomic Number	Atomic Type	Coordinates (Angstroms) X	Y	Z
1	6	0	-2.536530	0.294315	1.338481
2	6	0	-3.168428	0.667126	0.154915
3	6	0	-2.447254	0.727760	-1.027508
4	6	0	-1.099571	0.413994	-1.011608
5	6	0	-0.428894	0.036306	0.150784
6	6	0	-1.188797	-0.014090	1.326383
7	6	0	1.025971	-0.319972	0.259440
8	6	0	1.909525	-0.289081	-0.962978
9	8	0	1.474451	-0.631445	1.343650
10	9	0	-0.445253	0.488128	-2.184061
11	6	0	3.347035	-0.676693	-0.670118
12	1	0	-3.096197	0.245758	2.263929
13	1	0	-4.223648	0.911393	0.149804
14	1	0	-2.907126	1.013611	-1.964599
15	1	0	-0.673201	-0.306784	2.232179
16	1	0	1.464929	-0.946000	-1.717299
17	1	0	1.847420	0.713073	-1.398913
18	1	0	3.796071	-0.001999	0.060099
19	1	0	3.941912	-0.638422	-1.584205
20	1	0	3.407584	-1.687077	-0.263275

Ethyl 3-(2-fluorophenyl)-3-oxopropanoate (1g)
Table S7

conformer #	Energy (au) HF/6-31G(d)	B3LYP/6-31G(d)	Structure	conformer #	Energy (au) HF/6-31G(d)	B3LYP/6-31G(d)	Structure
1	-747.005	-751.311	trans	27	-747.004	-751.31	trans
2	-747.005	-751.311	trans	28	-747.003	-751.31	trans
3	-747.005	-751.311	trans	29	-747.003	-751.31	trans
4	-747.005	-751.311	trans	30	-747.003	-751.31	trans
5	-747.005	-751.311	trans	31	-747.003	-751.31	trans
6	-747.004	-751.311	trans	32	-747.003	-751.31	trans
7	-747.004	-751.311	trans	33	-747.003	-751.31	trans
8	-747.004	-751.311	trans	34	-747.003	-751.31	trans
9	-747.004	-751.311	trans	35	-747.003	-751.31	trans
10	-747.004	-751.311	trans	36	-747.003	-751.31	trans
11	-747.004	-751.311	trans	37	-747.003	-751.31	trans
12	-747.004	-751.311	trans	38	-747.003	-751.31	trans
13	-747.004	-751.311	trans	39	-747.003	-751.31	trans
14	-747.004	-751.311	trans	40	-747.003	-751.31	trans
15	-747.004	-751.311	trans	41	-747.003	-751.31	trans
16	-747.004	-751.311	trans	42	-747.003	-751.31	trans
17	-747.004	-751.311	trans	43	-747.003	-751.31	trans
18	-747.004	-751.311	trans	44	-747.003	-751.31	trans
19	-747.004	-751.31	trans	45	-747.003	-751.31	trans
20	-747.004	-751.31	trans	46	-747.003	-751.31	trans
21	-747.004	-751.31	trans	47	-746.997	-751.303	cis
22	-747.004	-751.31	trans	48	-746.997	-751.303	cis
23	-747.004	-751.31	trans	49	-746.997	-751.303	cis
24	-747.004	-751.31	trans	50	-746.998	-751.304	trans
25	-747.004	-751.31	trans	51	-746.998	-751.304	cis
26	-747.004	-751.31	trans				

Cis isomer

SCF Energy= -751.328498569 (Hartree)

Zero-point correction= 0.202597 (Hartree/Particle)

Thermal correction to Energy= 0.216754

Thermal correction to Enthalpy= 0.217698

Thermal correction to Gibbs Free Energy= 0.159070

Sum of electronic and zero-point Energies= -751.125902

Sum of electronic and thermal Energies= -751.111745

Sum of electronic and thermal Enthalpies= -751.110801

Sum of electronic and thermal Free Energies= -751.169428

Center Number	Atomic Number	Atomic Type	Coordinates (Angstroms) X	Y	Z
1	6	0	-3.858624	0.012043	-1.005165
2	6	0	-4.563759	-0.511846	0.072597
3	6	0	-3.916502	-0.755058	1.274579
4	6	0	-2.567474	-0.472396	1.390188
5	6	0	-1.821814	0.056272	0.328862
6	6	0	-2.508952	0.288877	-0.870564
7	6	0	-0.370799	0.361047	0.462589
8	6	0	0.329642	0.948196	-0.759063
9	8	0	0.248025	0.168572	1.483835
10	9	0	-1.993665	-0.725051	2.566259
11	6	0	1.784085	1.198993	-0.458680
12	8	0	2.502567	0.087905	-0.611232
13	6	0	3.900756	0.184135	-0.276477
14	8	0	2.239416	2.261293	-0.123723
15	6	0	4.515092	-1.174678	-0.492762
16	1	0	-4.358811	0.204008	-1.945425
17	1	0	-5.619972	-0.732483	-0.019594
18	1	0	-4.437650	-1.161946	2.131368
19	1	0	-1.974309	0.696683	-1.717828
20	1	0	-0.137060	1.896322	-1.030679
21	1	0	0.235102	0.260721	-1.602104
22	1	0	3.984349	0.511238	0.761294
23	1	0	4.358907	0.947205	-0.908457
24	1	0	4.415912	-1.487139	-1.533595
25	1	0	4.037883	-1.922370	0.142470
26	1	0	5.577456	-1.140743	-0.244595

Trans isomer
SCF Energy= -751.332775460 (Hartree)
Zero-point correction= 0.202599 (Hartree/Particle)
Thermal correction to Energy= 0.216712
Thermal correction to Enthalpy= 0.217656
Thermal correction to Gibbs Free Energy= 0.159100
Sum of electronic and zero-point Energies= -751.130176
Sum of electronic and thermal Energies= -751.116064
Sum of electronic and thermal Enthalpies= -751.115120
Sum of electronic and thermal Free Energies= -751.173676

Center Number	Atomic Number	Atomic Type	Coordinates (Angstroms) X	Y	Z
1	6	0	-4.150571	0.684905	-0.375352
2	6	0	-4.008900	-0.532273	-1.036900
3	6	0	-2.808181	-1.223079	-0.974689
4	6	0	-1.761663	-0.684430	-0.249485
5	6	0	-1.862346	0.529120	0.428428
6	6	0	-3.088680	1.200243	0.344040
7	6	0	-0.785301	1.169047	1.241373
8	6	0	0.566774	0.504343	1.391624
9	8	0	-0.997787	2.220705	1.804510
10	9	0	-0.615280	-1.388185	-0.215781
11	6	0	1.370290	0.558708	0.114485
12	8	0	2.465511	-0.190628	0.211663
13	6	0	3.341108	-0.192029	-0.935295
14	8	0	1.078284	1.213935	-0.852840
15	6	0	4.524340	-1.062964	-0.601502
16	1	0	-5.086710	1.226145	-0.423769
17	1	0	-4.833229	-0.946388	-1.604284
18	1	0	-2.665395	-2.169859	-1.478796
19	1	0	-3.171717	2.143950	0.867553
20	1	0	0.475099	-0.533335	1.712964
21	1	0	1.113695	1.052116	2.161096
22	1	0	3.635165	0.836778	-1.149492
23	1	0	2.783679	-0.569216	-1.794495
24	1	0	5.064214	-0.672895	0.262687
25	1	0	4.207815	-2.083977	-0.382894
26	1	0	5.209785	-1.090638	-1.450449

1-(4-Bromo-2-fluorophenyl)ethanone (1h)

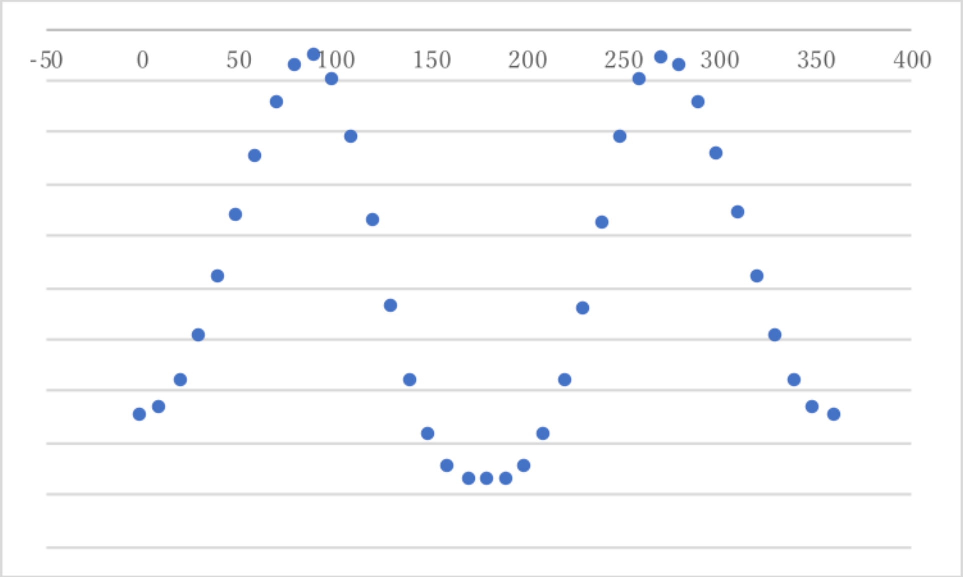

Figure S2

Table S8

conformer #	Angle	energy(au)	conformer #	Angle	energy(au)
1	-0.1214	-3023.2395	20	189.8786	-3023.24
2	9.8786	-3023.2393	21	199.8786	-3023.24
3	19.8786	-3023.2388	22	209.8786	-3023.24
4	29.8786	-3023.238	23	219.8786	-3023.24
5	39.8786	-3023.2368	24	229.8786	-3023.24
6	49.8786	-3023.2356	25	239.8786	-3023.24
7	59.8786	-3023.2345	26	249.8786	-3023.23
8	69.8786	-3023.2335	27	259.8786	-3023.23
9	79.8786	-3023.2328	28	269.8786	-3023.23
10	89.8786	-3023.2326	29	279.8786	-3023.23
11	99.8786	-3023.233	30	289.8786	-3023.23
12	109.8786	-3023.2341	31	299.8786	-3023.23
13	119.8786	-3023.2357	32	309.8786	-3023.24
14	129.8786	-3023.2374	33	319.8786	-3023.24
15	139.8786	-3023.2388	34	329.8786	-3023.24
16	149.8786	-3023.2399	35	339.8786	-3023.24
17	159.8786	-3023.2405	36	349.8786	-3023.24
18	169.8786	-3023.2407	37	359.8786	-3023.24
19	179.8786	-3023.2407			

Cis isomer
SCF Energy= -3057.83786245 (Hartree)
Zero-point correction= 0.119909 (Hartree/Particle)
Thermal correction to Energy= 0.129127
Thermal correction to Enthalpy= 0.130071
Thermal correction to Gibbs Free Energy= 0.084132
Sum of electronic and zero-point Energies= -3057.717954
Sum of electronic and thermal Energies= -3057.708735
Sum of electronic and thermal Enthalpies= -3057.707791
Sum of electronic and thermal Free Energies= -3057.753731

Center Number	Atomic Number	Atomic Type	Coordinates (Angstroms) X	Y	Z
1	6	0	-0.471748	-1.342566	-0.000569
2	6	0	-1.136054	-0.123067	-0.000055
3	6	0	-0.440107	1.074796	0.000359
4	6	0	0.943332	1.038176	0.000190
5	6	0	1.665064	-0.160990	-0.000301
6	6	0	0.912622	-1.341248	-0.000632
7	6	0	3.159898	-0.194501	-0.000579
8	6	0	3.832767	-1.545546	0.001759
9	8	0	3.818368	0.822240	-0.001873
10	1	0	4.908912	-1.388955	0.002812
11	1	0	3.551527	-2.125451	0.883927
12	1	0	3.553680	-2.127233	-0.879934
13	9	0	1.567028	2.213305	0.000721
14	35	0	-3.027283	-0.087699	0.000142
15	1	0	-1.022681	-2.272542	-0.000894
16	1	0	-0.942806	2.031867	0.000823
17	1	0	1.424920	-2.293767	-0.001034

Trans isomer
SCF Energy= -3057.84214367 (Hartree)
Zero-point correction= 0.120057 (Hartree/Particle)
Thermal correction to Energy= 0.130085
Thermal correction to Enthalpy= 0.131029
Thermal correction to Gibbs Free Energy= 0.082821
Sum of electronic and zero-point Energies= -3057.722086
Sum of electronic and thermal Energies= -3057.712059
Sum of electronic and thermal Enthalpies= -3057.711114
Sum of electronic and thermal Free Energies= -3057.759323---

Center Number	Atomic Number	Atomic Type	Coordinates (Angstroms) X	Y	Z
1	6	0	-0.542440	-1.371943	-0.000043
2	6	0	-1.148822	-0.119761	0.000013
3	6	0	-0.398757	1.044497	0.000108
4	6	0	0.980892	0.934963	0.000145
5	6	0	1.641819	-0.291668	0.000086
6	6	0	0.837725	-1.436762	-0.000004
7	6	0	3.128633	-0.491324	0.000115
8	6	0	4.049971	0.694320	0.000090
9	8	0	3.569262	-1.621868	0.000152
10	1	0	5.076511	0.334887	0.000014
11	1	0	3.871670	1.320490	-0.876056
12	1	0	3.871784	1.320413	0.876315
13	9	0	1.672544	2.082938	0.000232
14	35	0	-3.036488	0.005353	-0.000042
15	1	0	-1.139276	-2.273352	-0.000117
16	1	0	-0.855009	2.024305	0.000156
17	1	0	1.342751	-2.394360	-0.000045

1-(5-Bromo-2-fluorophenyl)ethanone (1i)

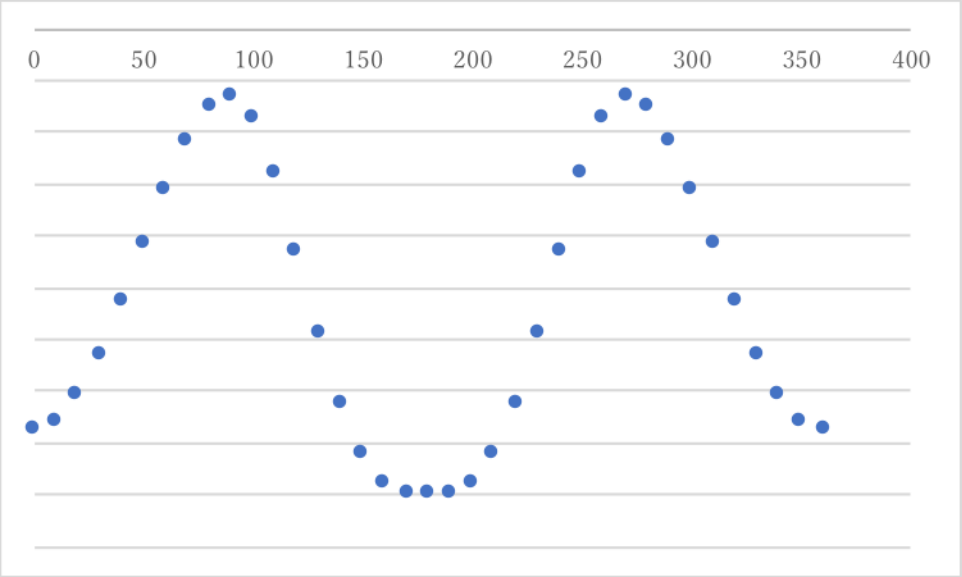

Figure S3

Table S9

conformer #	Angle	energy(au)	conformer #	Angle	energy(au)
1	-0.0152	-3023.24	20	189.9848	-3023.24
2	9.9848	-3023.24	21	199.9848	-3023.24
3	19.9848	-3023.24	22	209.9848	-3023.24
4	29.9848	-3023.24	23	219.9848	-3023.24
5	39.9848	-3023.24	24	229.9848	-3023.24
6	49.9848	-3023.24	25	239.9848	-3023.24
7	59.9848	-3023.23	26	249.9848	-3023.23
8	69.9848	-3023.23	27	259.9848	-3023.23
9	79.9848	-3023.23	28	269.9848	-3023.23
10	89.9848	-3023.23	29	279.9848	-3023.23
11	99.9848	-3023.23	30	289.9848	-3023.23
12	109.9848	-3023.23	31	299.9848	-3023.23
13	119.9848	-3023.24	32	309.9848	-3023.24
14	129.9848	-3023.24	33	319.9848	-3023.24
15	139.9848	-3023.24	34	329.9848	-3023.24
16	149.9848	-3023.24	35	339.9848	-3023.24
17	159.9848	-3023.24	36	349.9848	-3023.24
18	169.9848	-3023.24	37	359.9848	-3023.24
19	179.9848	-3023.24			

Cis isomer

SCF Energy= -3057.83712815 (Hartree)

Zero-point correction= 0.119905 (Hartree/Particle)

Thermal correction to Energy= 0.129145

Thermal correction to Enthalpy= 0.130089

Thermal correction to Gibbs Free Energy= 0.084163

Sum of electronic and zero-point Energies= -3057.717223

Sum of electronic and thermal Energies= -3057.707983

Sum of electronic and thermal Enthalpies= -3057.707039

Sum of electronic and thermal Free Energies= -3057.752965

Center Number	Atomic Number	Atomic Type	Coordinates (Angstroms) X	Y	Z
1	6	0	-1.012559	0.299832	-0.000036
2	6	0	-0.812762	1.674059	0.000026
3	6	0	0.483199	2.163666	0.000037
4	6	0	1.553518	1.286566	-0.000023
5	6	0	1.376416	-0.101316	-0.000080
6	6	0	0.058993	-0.574894	-0.000079
7	6	0	2.530782	-1.056491	-0.000149
8	6	0	2.225082	-2.533853	0.000265
9	8	0	3.675501	-0.662869	-0.000440
10	1	0	3.168407	-3.074710	0.000329
11	1	0	1.646546	-2.817960	-0.881862
12	1	0	1.646665	-2.817496	0.882618
13	9	0	2.772615	1.821547	0.000009
14	35	0	-2.780736	-0.388892	-0.000055
15	1	0	-1.652858	2.355273	0.000068
16	1	0	0.679137	3.227915	0.000094
17	1	0	-0.127637	-1.638957	-0.000122

Trans

SCF Energy= -3057.84149065 (Hartree)

Zero-point correction= 0.120078 (Hartree/Particle)

Thermal correction to Energy= 0.130103

Thermal correction to Enthalpy= 0.131048

Thermal correction to Gibbs Free Energy= 0.082786

Sum of electronic and zero-point Energies= -3057.721413

Sum of electronic and thermal Energies= -3057.711387

Sum of electronic and thermal Enthalpies= -3057.710443

Sum of electronic and thermal Free Energies= -3057.758705

Center Number	Atomic Number	Atomic Type	Coordinates (Angstroms) X	Y	Z
1	6	0	-1.043567	0.276236	0.000035
2	6	0	-0.821200	1.649859	0.000032
3	6	0	0.480893	2.120710	-0.000062
4	6	0	1.532616	1.220758	-0.000142
5	6	0	1.333445	-0.157841	-0.000147
6	6	0	0.010875	-0.614054	-0.000058
7	6	0	2.411332	-1.206331	-0.000261
8	6	0	3.856111	-0.799912	0.000180
9	8	0	2.091466	-2.375862	0.000001
10	1	0	4.468751	-1.698559	0.000528
11	1	0	4.083698	-0.189849	0.876331
12	1	0	4.084265	-0.190166	-0.876047
13	9	0	2.772710	1.730423	-0.000247
14	35	0	-2.824803	-0.378474	0.000162
15	1	0	-1.650756	2.343954	0.000101
16	1	0	0.694229	3.181475	-0.000076
17	1	0	-0.148165	-1.683928	-0.000070

1-(2-Fluoro-4-hydroxyphenyl)ethanone (1j)

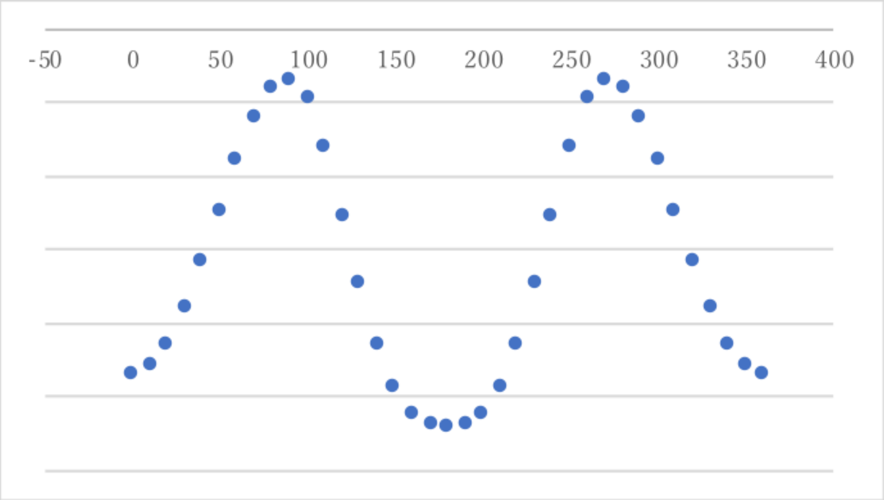

Figure S4

Table S10

conformer #	Angle	energy(au)	conformer #	Angle	energy(au)
1	-0.0019	-552.007	20	189.9981	-552.009
2	9.9981	-552.007	21	199.9981	-552.008
3	19.9981	-552.007	22	209.9981	-552.008
4	29.9981	-552.006	23	219.9981	-552.007
5	39.9981	-552.004	24	229.9981	-552.005
6	49.9981	-552.003	25	239.9981	-552.003
7	59.9981	-552.002	26	249.9981	-552.001
8	69.9981	-552	27	259.9981	-552
9	79.9981	-552	28	269.9981	-551.999
10	89.9981	-551.999	29	279.9981	-552
11	99.9981	-552	30	289.9981	-552
12	109.9981	-552.001	31	299.9981	-552.002
13	119.9981	-552.003	32	309.9981	-552.003
14	129.9981	-552.005	33	319.9981	-552.004
15	139.9981	-552.007	34	329.9981	-552.006
16	149.9981	-552.008	35	339.9981	-552.007
17	159.9981	-552.008	36	349.9981	-552.007
18	169.9981	-552.009	37	359.9981	-552.007
19	179.9981	-552.009			

Cis isomer

SCF Energy= -559.364088537 (Hartree)

Zero-point correction= 0.134710 (Hartree/Particle)

Thermal correction to Energy= 0.144491

Thermal correction to Enthalpy= 0.145436

Thermal correction to Gibbs Free Energy= 0.098908

Sum of electronic and zero-point Energies= -559.229378

Sum of electronic and thermal Energies= -559.219597

Sum of electronic and thermal Enthalpies= -559.218653

Sum of electronic and thermal Free Energies= -559.265180

Center Number	Atomic Number	Atomic Type	Coordinates (Angstroms) X	Y	Z
1	6	0	-1.470326	-1.409804	-0.000037
2	6	0	-2.195983	-0.217177	-0.000029
3	6	0	-1.522035	1.001393	-0.000003
4	6	0	-0.143889	1.011850	0.000014
5	6	0	0.627922	-0.159865	0.000011
6	6	0	-0.089908	-1.363344	-0.000015
7	6	0	2.113929	-0.145009	0.000029
8	6	0	2.831507	-1.476167	0.000083
9	8	0	2.750588	0.888635	-0.000106
10	1	0	3.901879	-1.283333	0.000109
11	1	0	2.572084	-2.066509	-0.881811
12	1	0	2.572025	-2.066481	0.881977
13	9	0	0.440833	2.209958	0.000045
14	8	0	-3.540285	-0.177524	-0.000041
15	1	0	-1.986277	-2.362879	-0.000058
16	1	0	-2.069891	1.933894	0.000006
17	1	0	0.452916	-2.298884	-0.000023
18	1	0	-3.893128	-1.071343	-0.000071

Trans

SCF Energy= -559.368463363 (Hartree)

Zero-point correction= 0.134756 (Hartree/Particle)

Thermal correction to Energy= 0.144453

Thermal correction to Enthalpy= 0.145397

Thermal correction to Gibbs Free Energy= 0.099459

Sum of electronic and zero-point Energies= -559.233708

Sum of electronic and thermal Energies= -559.224010

Sum of electronic and thermal Enthalpies= -559.223066

Sum of electronic and thermal Free Energies= -559.269005

Center Number	Atomic Number	Atomic Type	Coordinates (Angstroms) X	Y	Z
1	6	0	-1.608628	-1.338298	-0.000093
2	6	0	-2.218095	-0.079300	-0.000078
3	6	0	-1.433176	1.070426	-0.000132
4	6	0	-0.060672	0.942508	-0.000206
5	6	0	0.592825	-0.291718	-0.000212
6	6	0	-0.232906	-1.423669	-0.000145
7	6	0	2.067311	-0.509616	-0.000258
8	6	0	3.005330	0.666005	-0.000546
9	8	0	2.504236	-1.644759	-0.000028
10	1	0	4.027021	0.292460	-0.000749
11	1	0	2.836726	1.294810	0.875549
12	1	0	2.836329	1.294771	-0.876587
13	9	0	0.647508	2.083471	-0.000202
14	8	0	-3.552639	0.086936	-0.000026
15	1	0	-2.215949	-2.236576	-0.000056
16	1	0	-1.889164	2.050930	-0.000110
17	1	0	0.257997	-2.388598	-0.000140
18	1	0	-3.987954	-0.769673	-0.000032

1-(2,4-Difluorophenyl)ethanone (1k)

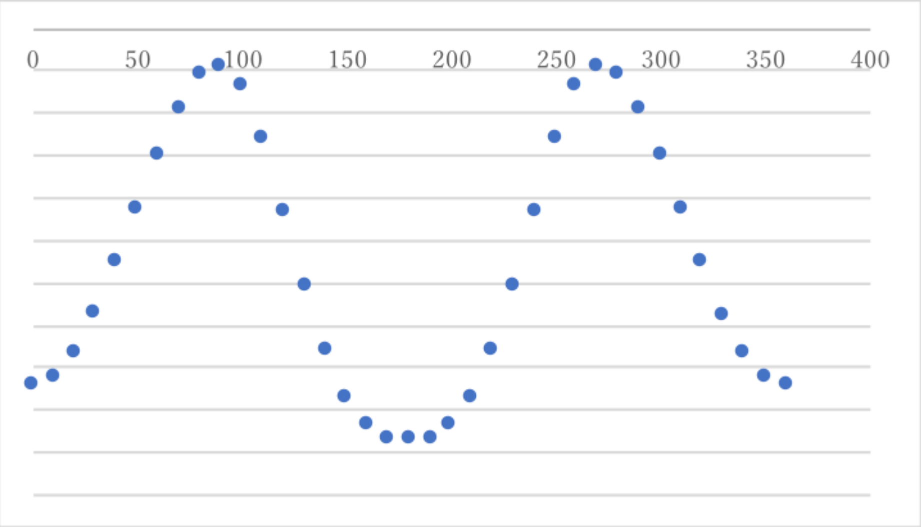

Figure S5

Table S11

conformer #	Angle	energy(au)	conformer #	Angle	energy(au)
1	0.0521	-575.624	20	190.0521	-575.626
2	10.0521	-575.624	21	200.0521	-575.625
3	20.0521	-575.624	22	210.0521	-575.625
4	30.0521	-575.623	23	220.0521	-575.624
5	40.0521	-575.622	24	230.0521	-575.622
6	50.0521	-575.62	25	240.0521	-575.62
7	60.0521	-575.619	26	250.0521	-575.619
8	70.0521	-575.618	27	260.0521	-575.617
9	80.0521	-575.617	28	270.0521	-575.617
10	90.0521	-575.617	29	280.0521	-575.617
11	100.0521	-575.617	30	290.0521	-575.618
12	110.0521	-575.619	31	300.0521	-575.619
13	120.0521	-575.62	32	310.0521	-575.62
14	130.0521	-575.622	33	320.0521	-575.622
15	140.0521	-575.624	34	330.0521	-575.623
16	150.0521	-575.625	35	340.0521	-575.624
17	160.0521	-575.625	36	350.0521	-575.624
18	170.0521	-575.626	37	360.0521	-575.624
19	180.0521	-575.626			

Cis isomer
SCF Energy= -583.374559187 (Hartree)
Zero-point correction= 0.121993 (Hartree/Particle)
Thermal correction to Energy= 0.131491
Thermal correction to Enthalpy= 0.132436
Thermal correction to Gibbs Free Energy= 0.085769
Sum of electronic and zero-point Energies= -583.252566
Sum of electronic and thermal Energies= -583.243068
Sum of electronic and thermal Enthalpies= -583.242124
Sum of electronic and thermal Free Energies= -583.288790

Center Number	Atomic Number	Atomic Type	Coordinates (Angstroms) X	Y	Z
1	6	0	-1.470967	-1.431039	0.000111
2	6	0	-2.172321	-0.238106	0.000037
3	6	0	-1.538768	0.989531	-0.000062
4	6	0	-0.156335	1.009611	-0.000066
5	6	0	0.614574	-0.160473	-0.000003
6	6	0	-0.088827	-1.372021	0.000074
7	6	0	2.107030	-0.134740	0.000021
8	6	0	2.833761	-1.458541	-0.000576
9	8	0	2.728461	0.905805	0.000471
10	1	0	2.577660	-2.050048	-0.882656
11	1	0	3.902575	-1.257984	-0.000788
12	1	0	2.578150	-2.050542	0.881317
13	9	0	0.419835	2.208760	-0.000198
14	9	0	-3.507827	-0.266983	0.000057
15	1	0	-2.001974	-2.372926	0.000190
16	1	0	-2.100900	1.913086	-0.000138
17	1	0	0.463873	-2.301321	0.000128

Trans isomer
SCF Energy= -583.378812160 (Hartree)
Zero-point correction= 0.122097 (Hartree/Particle)
Thermal correction to Energy= 0.131479
Thermal correction to Enthalpy= 0.132423
Thermal correction to Gibbs Free Energy= 0.086856
Sum of electronic and zero-point Energies= -583.256715
Sum of electronic and thermal Energies= -583.247333
Sum of electronic and thermal Enthalpies= -583.246389
Sum of electronic and thermal Free Energies= -583.291957

Center Number	Atomic Number	Atomic Type	Coordinates (Angstroms) X	Y	Z
1	6	0	-1.612893	-1.354994	0.000046
2	6	0	-2.196503	-0.097437	0.000071
3	6	0	-1.448986	1.063989	-0.000007
4	6	0	-0.071428	0.943398	-0.000120
5	6	0	0.579417	-0.290122	-0.000165
6	6	0	-0.234242	-1.430018	-0.000069
7	6	0	2.062095	-0.502237	-0.000296
8	6	0	2.994023	0.675963	-0.000311
9	8	0	2.496214	-1.635979	0.000029
10	1	0	2.821815	1.303684	0.875919
11	1	0	4.017331	0.307263	-0.000310
12	1	0	2.821802	1.303691	-0.876528
13	9	0	0.630657	2.084673	-0.000191
14	9	0	-3.528547	0.002490	0.000188
15	1	0	-2.234323	-2.240104	0.000113
16	1	0	-1.918986	2.037522	0.000024
17	1	0	0.265412	-2.390113	-0.000092

1-(2,4,5-Trifluorophenyl)ethanone (1l)

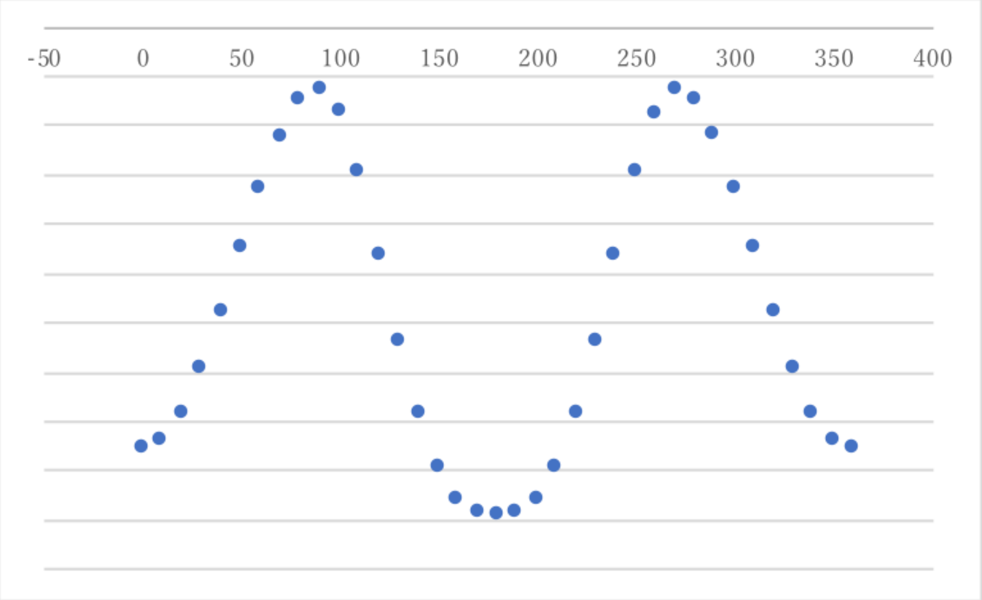

Figure S6

Table S12

conformer #	Angle	energy(au)	conformer #	Angle	energy(au)
1	-0.0521	-673.404	20	189.9479	-673.405
2	9.9479	-673.403	21	199.9479	-673.405
3	19.9479	-673.403	22	209.9479	-673.404
4	29.9479	-673.402	23	219.9479	-673.403
5	39.9479	-673.401	24	229.9479	-673.401
6	49.9479	-673.399	25	239.9479	-673.4
7	59.9479	-673.398	26	249.9479	-673.398
8	69.9479	-673.397	27	259.9479	-673.397
9	79.9479	-673.396	28	269.9479	-673.396
10	89.9479	-673.396	29	279.9479	-673.396
11	99.9479	-673.397	30	289.9479	-673.397
12	109.9479	-673.398	31	299.9479	-673.398
13	119.9479	-673.4	32	309.9479	-673.399
14	129.9479	-673.401	33	319.9479	-673.401
15	139.9479	-673.403	34	329.9479	-673.402
16	149.9479	-673.404	35	339.9479	-673.403
17	159.9479	-673.405	36	349.9479	-673.403
18	169.9479	-673.405	37	359.9479	-673.404
19	179.9479	-673.405			

Cis isomer
SCF Energy= -682.606414558 (Hartree)
Zero-point correction= 0.113883 (Hartree/Particle)
Thermal correction to Energy= 0.124216
Thermal correction to Enthalpy= 0.125160
Thermal correction to Gibbs Free Energy= 0.076941
Sum of electronic and zero-point Energies= -682.492532
Sum of electronic and thermal Energies= -682.482198
Sum of electronic and thermal Enthalpies= -682.481254
Sum of electronic and thermal Free Energies= -682.529474

Center Number	Atomic Number	Atomic Type	Coordinates (Angstroms) X	Y	Z
1	6	0	1.461239	0.914014	-0.000094
2	6	0	1.952107	-0.384773	-0.000006
3	6	0	1.086253	-1.457674	0.000128
4	6	0	-0.277237	-1.218109	0.000141
5	6	0	-0.809763	0.075507	0.000054
6	6	0	0.104876	1.136742	-0.000043
7	6	0	-2.282247	0.336606	-0.000012
8	6	0	-2.740075	1.774096	0.000781
9	8	0	-3.085859	-0.569105	-0.000743
10	1	0	-3.827314	1.784372	0.001055
11	1	0	-2.373987	2.304024	0.883239
12	1	0	-2.374499	2.304742	-0.881466
13	9	0	-1.068831	-2.286907	0.000317
14	9	0	3.266078	-0.583863	-0.000041
15	9	0	2.321390	1.935911	-0.000224
16	1	0	1.465514	-2.470294	0.000224
17	1	0	-0.235386	2.162802	-0.000111

Trans isomer
SCF Energy= -682.610970827 (Hartree)
Zero-point correction= 0.113933 (Hartree/Particle)
Thermal correction to Energy= 0.124180
Thermal correction to Enthalpy= 0.125124
Thermal correction to Gibbs Free Energy= 0.077538
Sum of electronic and zero-point Energies= -682.497037
Sum of electronic and thermal Energies= -682.486791
Sum of electronic and thermal Enthalpies= -682.485847
Sum of electronic and thermal Free Energies= -682.533433

Center Number	Atomic Number	Atomic Type	Coordinates (Angstroms) X	Y	Z
1	6	0	1.555199	0.846187	-0.000024
2	6	0	1.931773	-0.493351	0.000001
3	6	0	0.978988	-1.489110	0.000020
4	6	0	-0.357169	-1.125771	0.000012
5	6	0	-0.772598	0.203471	-0.000014
6	6	0	0.226141	1.184567	-0.000030
7	6	0	-2.192979	0.686455	-0.000025
8	6	0	-3.324267	-0.299990	-0.000025
9	8	0	-2.405321	1.880722	0.000022
10	1	0	-4.262470	0.250048	-0.000043
11	1	0	-3.270183	-0.948627	-0.876353
12	1	0	-3.270202	-0.948589	0.876334
13	9	0	-1.251190	-2.122612	0.000034
14	9	0	3.224028	-0.803773	0.000007
15	9	0	2.505978	1.783859	-0.000042
16	1	0	1.268954	-2.530679	0.000042
17	1	0	-0.072802	2.224683	-0.000048

1-(2-Fluoro-5-nitrophenyl)ethanone (**1m**)

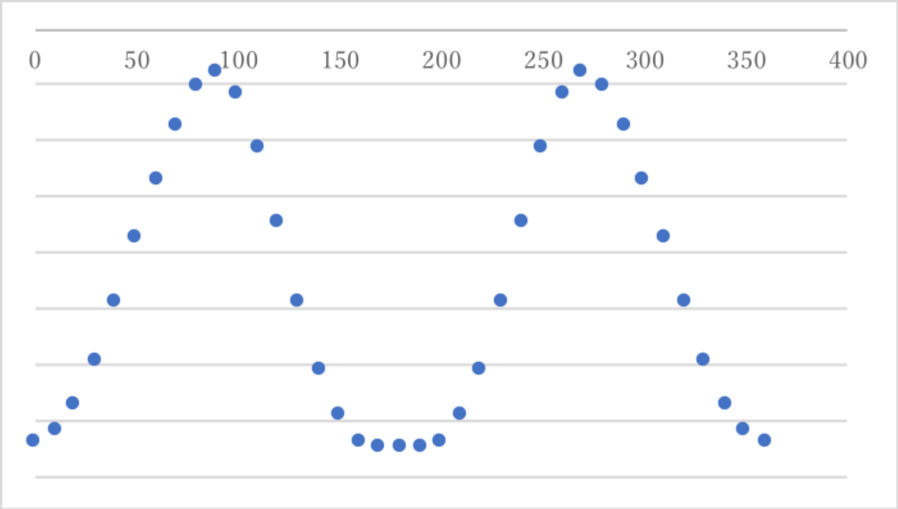

Figure S7

Table S13

conformer #	Angle	energy(au)	conformer #	Angle	energy(au)
1	0.0074	-679.515	20	190.0074	-679.515
2	10.0074	-679.515	21	200.0074	-679.515
3	20.0074	-679.515	22	210.0074	-679.515
4	30.0074	-679.514	23	220.0074	-679.514
5	40.0074	-679.513	24	230.0074	-679.513
6	50.0074	-679.512	25	240.0074	-679.511
7	60.0074	-679.511	26	250.0074	-679.51
8	70.0074	-679.51	27	260.0074	-679.509
9	80.0074	-679.509	28	270.0074	-679.509
10	90.0074	-679.509	29	280.0074	-679.509
11	100.0074	-679.509	30	290.0074	-679.51
12	110.0074	-679.51	31	300.0074	-679.511
13	120.0074	-679.511	32	310.0074	-679.512
14	130.0074	-679.513	33	320.0074	-679.513
15	140.0074	-679.514	34	330.0074	-679.514
16	150.0074	-679.515	35	340.0074	-679.515
17	160.0074	-679.515	36	350.0074	-679.515
18	170.0074	-679.515	37	360.0074	-679.515
19	180.0074	-679.515			

Cis isomer

SCF Energy= -688.638505183 (Hartree)

Zero-point correction= 0.132908 (Hartree/Particle)

Thermal correction to Energy= 0.143215

Thermal correction to Enthalpy= 0.144159

Thermal correction to Gibbs Free Energy= 0.096175

Sum of electronic and zero-point Energies= -688.505597

Sum of electronic and thermal Energies= -688.495290

Sum of electronic and thermal Enthalpies= -688.494346

Sum of electronic and thermal Free Energies= -688.542330

Center Number	Atomic Number	Atomic Type	Coordinates (Angstroms) X	Y	Z
1	6	0	1.388909	-0.136681	0.000003
2	6	0	1.319428	-1.522567	-0.000024
3	6	0	0.073417	-2.118417	-0.000042
4	6	0	-1.066305	-1.327954	-0.000029
5	6	0	-1.012884	0.073337	-0.000004
6	6	0	0.254564	0.653466	0.000010
7	6	0	-2.247718	0.926119	0.000015
8	6	0	-2.065107	2.422060	-0.000145
9	8	0	-3.351608	0.431966	0.000163
10	1	0	-1.511503	2.752041	-0.882295
11	1	0	-1.511469	2.752220	0.881917
12	1	0	-3.049287	2.884109	-0.000166
13	9	0	-2.230902	-1.959254	-0.000056
14	7	0	2.701401	0.510978	0.000021
15	1	0	-0.037586	-3.194337	-0.000066
16	1	0	0.375352	1.726599	0.000030
17	1	0	2.226325	-2.109878	-0.000032
18	8	0	2.734388	1.726085	0.000058
19	8	0	3.682584	-0.205494	0.000001

Trans isomer

SCF Energy= -688.642185492 (Hartree)

Zero-point correction= 0.133123 (Hartree/Particle)

Thermal correction to Energy= 0.144184

Thermal correction to Enthalpy= 0.145128

Thermal correction to Gibbs Free Energy= 0.095036

Sum of electronic and zero-point Energies= -688.509062

Sum of electronic and thermal Energies= -688.498001

Sum of electronic and thermal Enthalpies= -688.497057

Sum of electronic and thermal Free Energies= -688.547150

Center Number	Atomic Number	Atomic Type	Coordinates (Angstroms) X	Y	Z
1	6	0	-1.422326	0.129062	0.000007
2	6	0	-1.313458	1.514861	0.000017
3	6	0	-0.052599	2.076957	0.000018
4	6	0	1.060299	1.248701	0.000009
5	6	0	0.967875	-0.143452	-0.000002
6	6	0	-0.313928	-0.691901	-0.000003
7	6	0	2.121885	-1.111873	-0.000015
8	6	0	3.533313	-0.604859	0.000026
9	8	0	1.879823	-2.298608	-0.000024
10	1	0	3.718356	0.018801	0.876721
11	1	0	3.718403	0.018794	-0.876665
12	1	0	4.207386	-1.458250	0.000046
13	9	0	2.254871	1.843074	0.000010
14	7	0	-2.756702	-0.476631	0.000008
15	1	0	0.089599	3.149122	0.000026
16	1	0	-0.418629	-1.767864	-0.000011
17	1	0	-2.203690	2.127069	0.000024
18	8	0	-3.713589	0.272858	-0.000020
19	8	0	-2.829117	-1.688700	-0.000003

1-(2-Fluoro-4-methoxyphenyl)ethanone (1n)

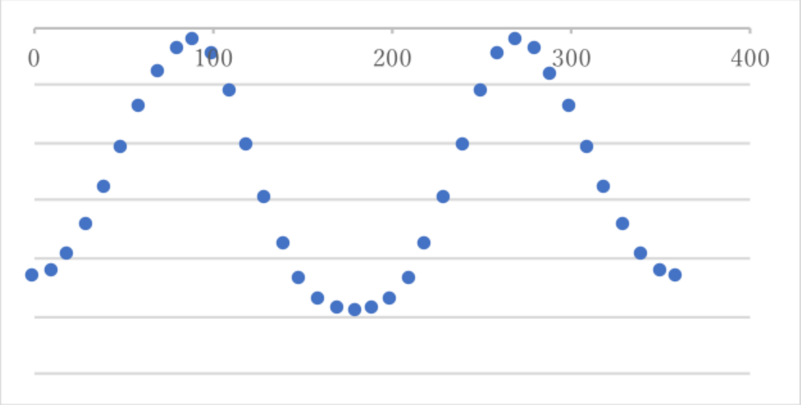

Figure S8

Table S14

conformer #	Angle	energy(au)	conformer #	Angle	energy(au)
1	0.0128	-590.859	20	190.0128	-590.86
2	10.0128	-590.858	21	200.0128	-590.859
3	20.0128	-590.858	22	210.0128	-590.859
4	30.0128	-590.857	23	220.0128	-590.858
5	40.0128	-590.856	24	230.0128	-590.856
6	50.0128	-590.854	25	240.0128	-590.854
7	60.0128	-590.853	26	250.0128	-590.852
8	70.0128	-590.852	27	260.0128	-590.851
9	80.0128	-590.851	28	270.0128	-590.851
10	90.0128	-590.851	29	280.0128	-590.851
11	100.0128	-590.851	30	290.0128	-590.852
12	110.0128	-590.852	31	300.0128	-590.853
13	120.0128	-590.854	32	310.0128	-590.854
14	130.0128	-590.856	33	320.0128	-590.856
15	140.0128	-590.858	34	330.0128	-590.857
16	150.0128	-590.859	35	340.0128	-590.858
17	160.0128	-590.859	36	350.0128	-590.858
18	170.0128	-590.86	37	360.0128	-590.859
19	180.0128	-590.86			

Cis isomer
SCF Energy= -598.661679982 (Hartree)
Zero-point correction= 0.162972 (Hartree/Particle)
Thermal correction to Energy= 0.174259
Thermal correction to Enthalpy= 0.175203
Thermal correction to Gibbs Free Energy= 0.124468
Sum of electronic and zero-point Energies= -598.498708
Sum of electronic and thermal Energies= -598.487421
Sum of electronic and thermal Enthalpies= -598.486477
Sum of electronic and thermal Free Energies= -598.537212

Center Number	Atomic Number	Atomic Type	Coordinates (Angstroms) X	Y	Z
1	6	0	-0.926282	-1.592909	0.000087
2	6	0	-1.762721	-0.469983	0.000025
3	6	0	-1.198897	0.804161	-0.000064
4	6	0	0.178423	0.932831	-0.000061
5	6	0	1.050692	-0.159526	0.000029
6	6	0	0.439644	-1.426053	0.000082
7	6	0	2.528646	-0.008889	0.000110
8	6	0	3.363560	-1.269346	-0.000150
9	8	0	3.069238	1.078525	0.000348
10	1	0	3.157685	-1.880355	-0.882021
11	1	0	3.157867	-1.880574	0.881614
12	1	0	4.412150	-0.980852	-0.000210
13	9	0	0.648487	2.181215	-0.000181
14	8	0	-3.082126	-0.710018	0.000048
15	1	0	-1.374419	-2.577527	0.000145
16	1	0	-1.793927	1.705854	-0.000140
17	1	0	1.063240	-2.310004	0.000140
18	6	0	-3.972570	0.395675	0.000040
19	1	0	-3.838380	1.009365	0.894382
20	1	0	-4.972481	-0.029878	0.000094
21	1	0	-3.838450	1.009299	-0.894358

Trans isomer
SCF Energy= -598.665909610 (Hartree)
Zero-point correction= 0.163164 (Hartree/Particle)
Thermal correction to Energy= 0.174280
Thermal correction to Enthalpy= 0.175224
Thermal correction to Gibbs Free Energy= 0.125915
Sum of electronic and zero-point Energies= -598.502746
Sum of electronic and thermal Energies= -598.491630
Sum of electronic and thermal Enthalpies= -598.490685
Sum of electronic and thermal Free Energies= -598.539995

Center Number	Atomic Number	Atomic Type	Coordinates (Angstroms) X	Y	Z
1	6	0	-1.031209	-1.595657	-0.000028
2	6	0	-1.792255	-0.417346	0.000014
3	6	0	-1.147548	0.817491	0.000035
4	6	0	0.235928	0.847697	0.000017
5	6	0	1.029731	-0.295218	-0.000026
6	6	0	0.340065	-1.520085	-0.000046
7	6	0	2.518752	-0.337937	-0.000048
8	6	0	3.314343	0.938906	-0.000047
9	8	0	3.085655	-1.414309	0.000006
10	1	0	3.075156	1.544269	0.876274
11	1	0	3.075103	1.544317	-0.876319
12	1	0	4.372417	0.685898	-0.000080
13	9	0	0.799278	2.067592	0.000039
14	8	0	-3.124445	-0.570538	0.000027
15	1	0	-1.546257	-2.547216	-0.000046
16	1	0	-1.681855	1.756363	0.000067
17	1	0	0.941375	-2.420225	-0.000077
18	6	0	-3.939941	0.591178	0.000111
19	1	0	-3.765137	1.194552	0.894554
20	1	0	-4.965916	0.233037	0.000145
21	1	0	-3.765222	1.194621	-0.894303

1-(2,4-Difluorophenyl)-1-propanone (1o)
Table S15

conformer #	Energy (au) HF/6-31G(d)	B3LYP/6-31G(d)	Structure
1	-619.212	-622.672	trans
2	-619.209	-622.67	trans
3	-619.209	-622.67	trans
4	-619.205	-622.666	cis
5	-619.205	-622.666	cis
6	-619.203	-622.663	cis

Cis isomer
SCF Energy= -622.689555817 (Hartree)
Zero-point correction= 0.150686 (Hartree/Particle)
Thermal correction to Energy= 0.161488
Thermal correction to Enthalpy= 0.162432
Thermal correction to Gibbs Free Energy= 0.113225
Sum of electronic and zero-point Energies= -622.538869
Sum of electronic and thermal Energies= -622.528068
Sum of electronic and thermal Enthalpies= -622.527124
Sum of electronic and thermal Free Energies= -622.576331

Center Number	Atomic Number	Atomic Type	Coordinates (Angstroms) X	Y	Z
1	6	0	2.249763	-1.251811	0.374622
2	6	0	3.124755	-0.195113	0.555212
3	6	0	2.713424	1.121568	0.485461
4	6	0	1.380279	1.374611	0.219522
5	6	0	0.441950	0.352662	0.024997
6	6	0	0.922253	-0.960481	0.115577
7	6	0	-0.994311	0.639736	-0.272140
8	6	0	-1.957796	-0.531046	-0.295336
9	8	0	-1.384637	1.766344	-0.488343
10	9	0	1.018218	2.654147	0.168346
11	9	0	4.411732	-0.449654	0.808225
12	6	0	-3.395367	-0.105779	-0.533574
13	1	0	2.608115	-2.270072	0.436834
14	1	0	3.407622	1.936982	0.634262
15	1	0	0.237099	-1.784723	-0.026021
16	1	0	-1.631046	-1.228681	-1.074472
17	1	0	-1.864120	-1.077761	0.648991
18	1	0	-3.498025	0.414899	-1.486195
19	1	0	-3.738736	0.571853	0.249242
20	1	0	-4.051074	-0.977879	-0.545208

Trans isomer
SCF Energy= -622.693948416 (Hartree)
Zero-point correction= 0.150580 (Hartree/Particle)
Thermal correction to Energy= 0.161381
Thermal correction to Enthalpy= 0.162325
Thermal correction to Gibbs Free Energy= 0.112921
Sum of electronic and zero-point Energies= -622.543368
Sum of electronic and thermal Energies= -622.532568
Sum of electronic and thermal Enthalpies= -622.531623
Sum of electronic and thermal Free Energies= -622.581028

Center Number	Atomic Number	Atomic Type	Coordinates (Angstroms) X	Y	Z
1	6	0	2.694848	-1.206340	-0.225532
2	6	0	3.162645	-0.158956	0.552488
3	6	0	2.331270	0.836386	1.027263
4	6	0	0.988962	0.764624	0.702249
5	6	0	0.452100	-0.260864	-0.075856
6	6	0	1.347543	-1.239241	-0.526314
7	6	0	-0.985792	-0.414494	-0.473919
8	6	0	-2.009422	0.599417	-0.026950
9	8	0	-1.306217	-1.364683	-1.158168
10	9	0	0.206249	1.744169	1.176843
11	9	0	4.462365	-0.103552	0.858034
12	6	0	-3.410716	0.288953	-0.519406
13	1	0	3.378566	-1.966599	-0.578075
14	1	0	2.710443	1.647438	1.633242
15	1	0	0.938164	-2.038277	-1.130742
16	1	0	-1.974463	0.655436	1.065730
17	1	0	-1.677640	1.586096	-0.365613
18	1	0	-3.749888	-0.682124	-0.156121
19	1	0	-4.110383	1.049875	-0.169747
20	1	0	-3.448535	0.262737	-1.609307

2-Chloro-1-(2,4-difluorophenyl)ethanone (1p)

Table S16

conformer #	Energy (au) HF/6-31G(d)	B3LYP/6-31G(d)	Structure
1	-1039.07	-1042.95	trans
2	-1039.07	-1042.95	trans
3	-1039.07	-1042.95	trans
4	-1039.07	-1042.95	trans
5	-1039.07	-1042.95	trans
6	-1039.06	-1042.94	cis
7	-1039.06	-1042.94	cis
8	-1039.06	-1042.94	cis

Cis isomer

SCF Energy= -1042.99709031 (Hartree)

Zero-point correction= 0.113816 (Hartree/Particle)

Thermal correction to Energy= 0.124169

Thermal correction to Enthalpy= 0.125113

Thermal correction to Gibbs Free Energy= 0.075936

Sum of electronic and zero-point Energies= -1042.883274

Sum of electronic and thermal Energies= -1042.872922

Sum of electronic and thermal Enthalpies= -1042.871977

Sum of electronic and thermal Free Energies= -1042.921155

Center Number	Atomic Number	Atomic Type	Coordinates (Angstroms) X	Y	Z
1	6	0	-1.671578	0.616190	0.970262
2	6	0	-2.469619	0.329803	-0.124317
3	6	0	-1.950062	-0.134718	-1.317736
4	6	0	-0.582073	-0.304467	-1.406349
5	6	0	0.280175	-0.034135	-0.336136
6	6	0	-0.308150	0.422619	0.850450
7	6	0	1.749948	-0.181208	-0.468381
8	6	0	2.599410	-0.207824	0.796916
9	8	0	2.311770	-0.255695	-1.536376
10	9	0	-0.104507	-0.765197	-2.558433
11	9	0	-3.789041	0.503277	-0.029316
12	17	0	2.224989	-1.633991	1.821330
13	1	0	-2.116945	0.976889	1.886959
14	1	0	-2.588781	-0.362048	-2.159781
15	1	0	0.315697	0.640898	1.706175
16	1	0	2.460652	0.680268	1.410983
17	1	0	3.638016	-0.290761	0.493749

Trans isomer
SCF Energy= -1043.00236603 (Hartree)
Zero-point correction= 0.113565 (Hartree/Particle)
Thermal correction to Energy= 0.123880
Thermal correction to Enthalpy= 0.124824
Thermal correction to Gibbs Free Energy= 0.076067
Sum of electronic and zero-point Energies= -1042.888801
Sum of electronic and thermal Energies= -1042.878486
Sum of electronic and thermal Enthalpies= -1042.877542
Sum of electronic and thermal Free Energies= -1042.926299

Center Number	Atomic Number	Atomic Type	Coordinates (Angstroms) X	Y	Z
1	6	0	-2.044731	0.839985	0.878064
2	6	0	-2.519812	0.248266	-0.282581
3	6	0	-1.689489	-0.401921	-1.175179
4	6	0	-0.341469	-0.450310	-0.877590
5	6	0	0.201932	0.123561	0.271436
6	6	0	-0.691143	0.768587	1.137325
7	6	0	1.642179	0.116875	0.667450
8	6	0	2.630953	-0.575484	-0.253439
9	8	0	1.993951	0.649996	1.691577
10	9	0	0.449818	-1.085230	-1.754760
11	9	0	-3.824907	0.304860	-0.553792
12	17	0	4.298472	-0.497281	0.354114
13	1	0	-2.729307	1.339185	1.550059
14	1	0	-2.074430	-0.858624	-2.076162
15	1	0	-0.278109	1.216749	2.031444
16	1	0	2.611248	-0.114342	-1.239482
17	1	0	2.364744	-1.624873	-0.368485

5. ^{1}H and ^{13}C {^{1}H} NMR spectra of 1a-p, and 3-5.

^{1}H NMR (400 MHz, $CDCl_3$, ppm)

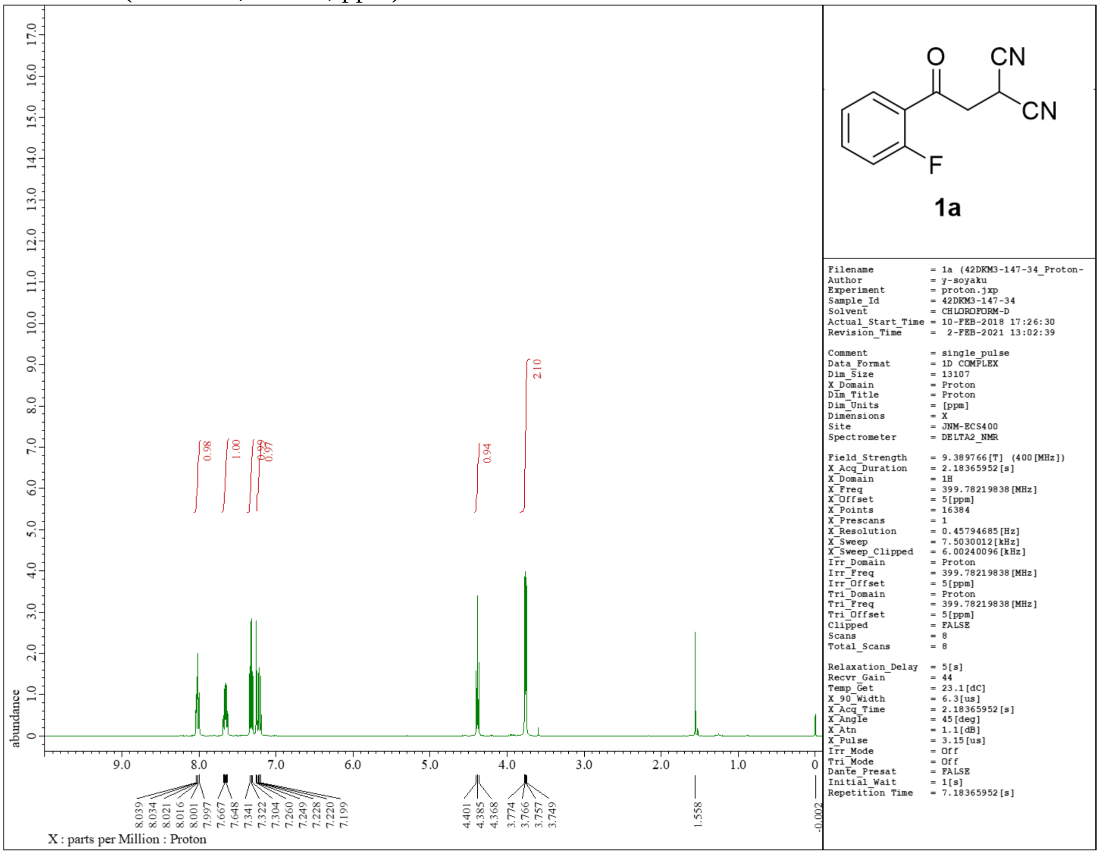

^{13}C{^{1}H} NMR (100 MHz, $CDCl_3$, ppm)

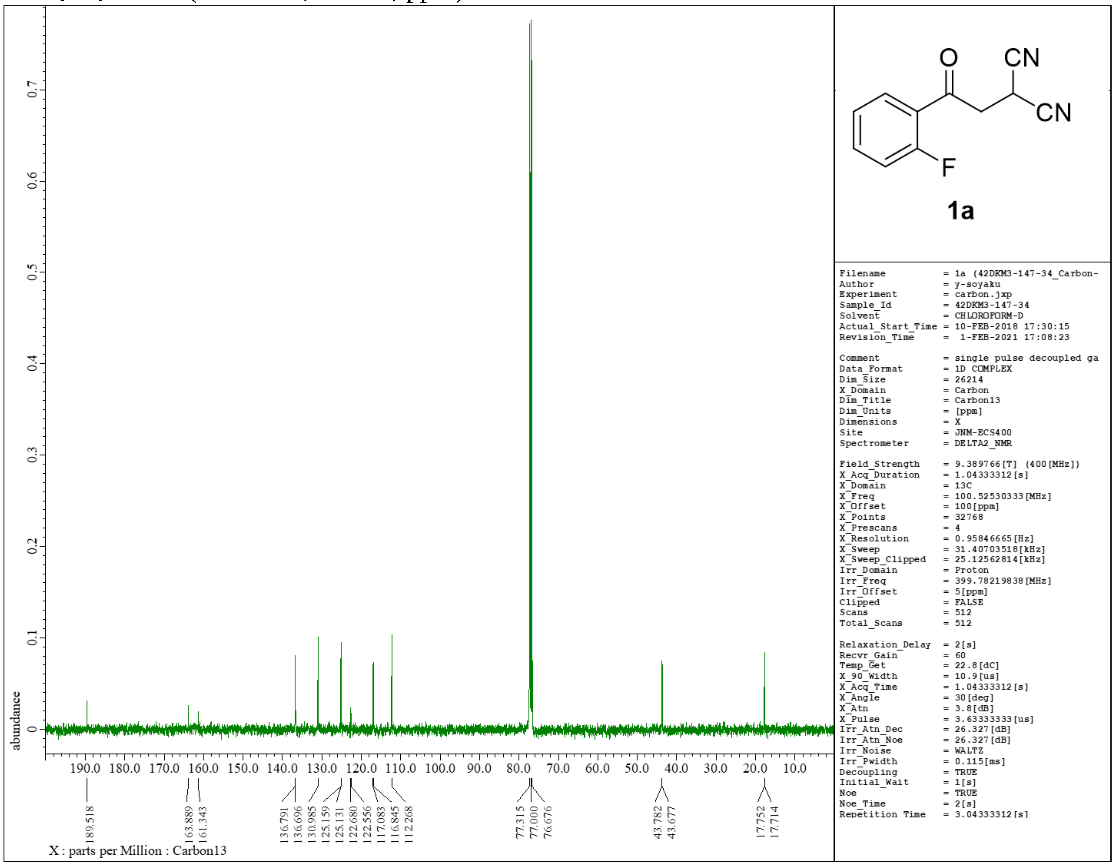

^{1}H NMR (400 MHz, $CDCl_3$, ppm)

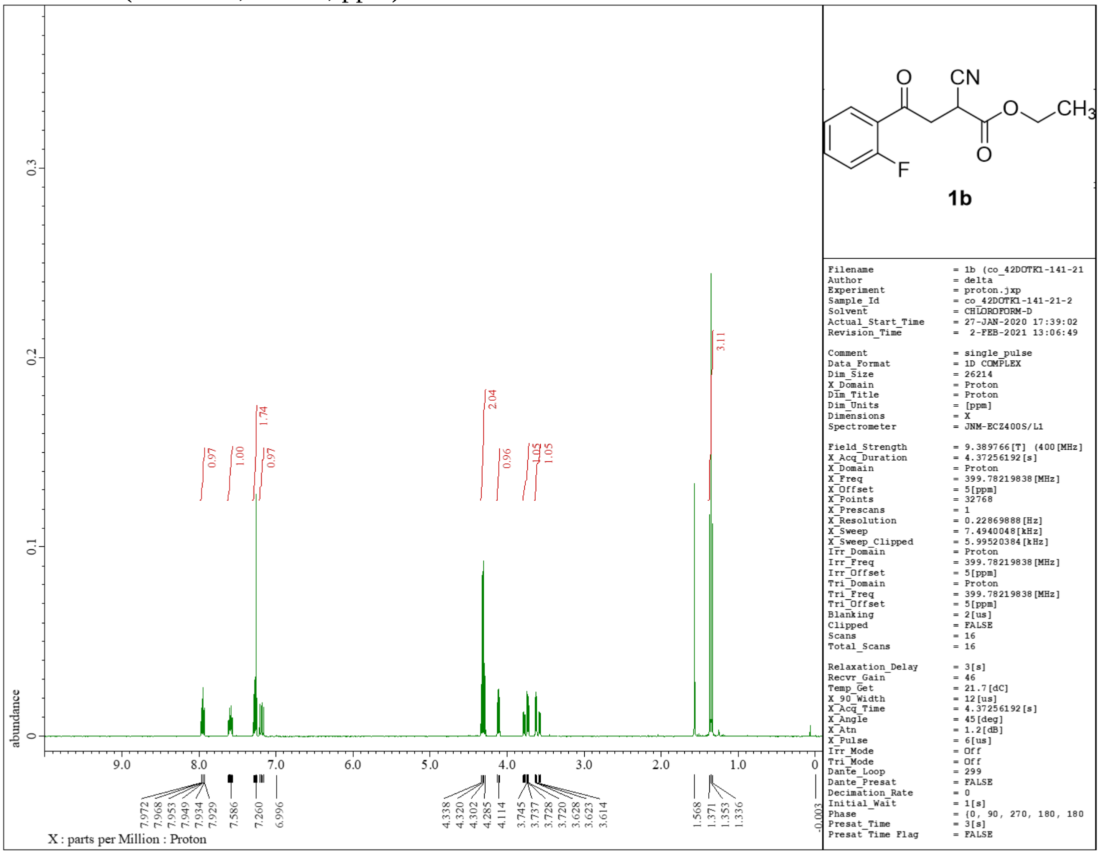

$^{13}C\{^{1}H\}$ NMR (100 MHz, $CDCl_3$, ppm)

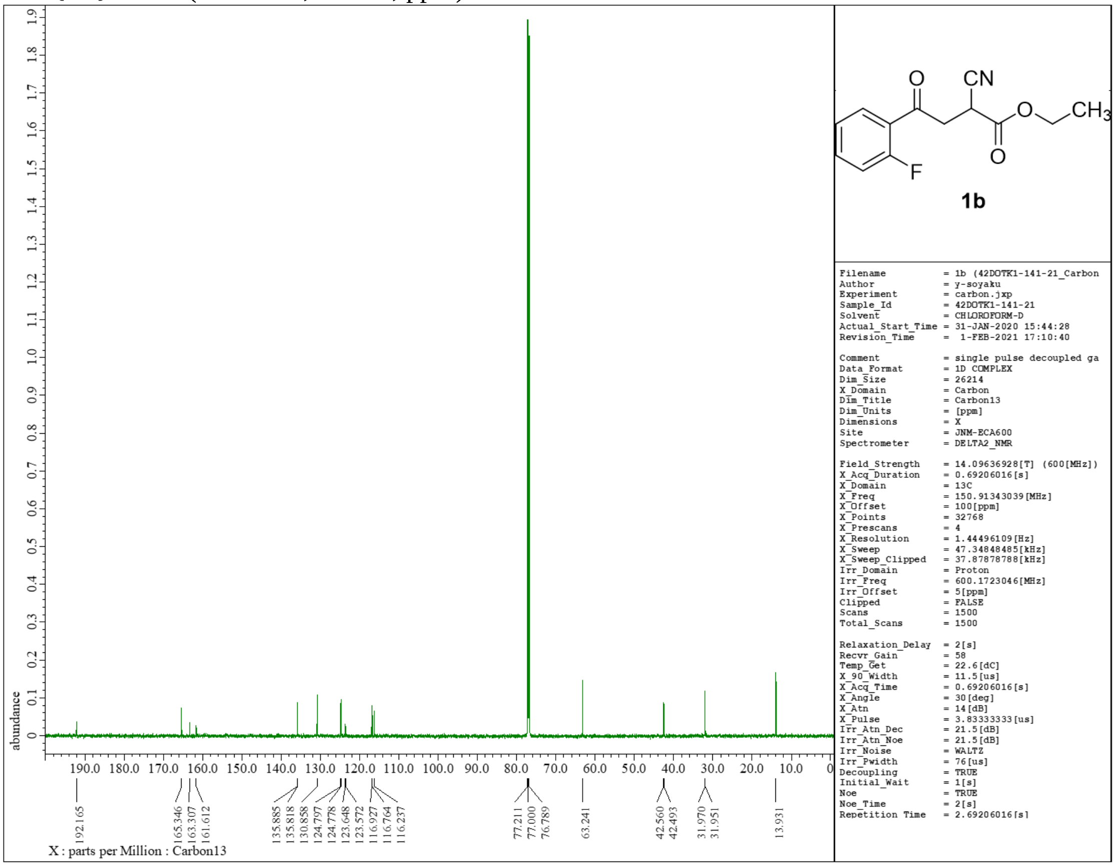

^{1}H NMR (400 MHz, $CDCl_3$, ppm)

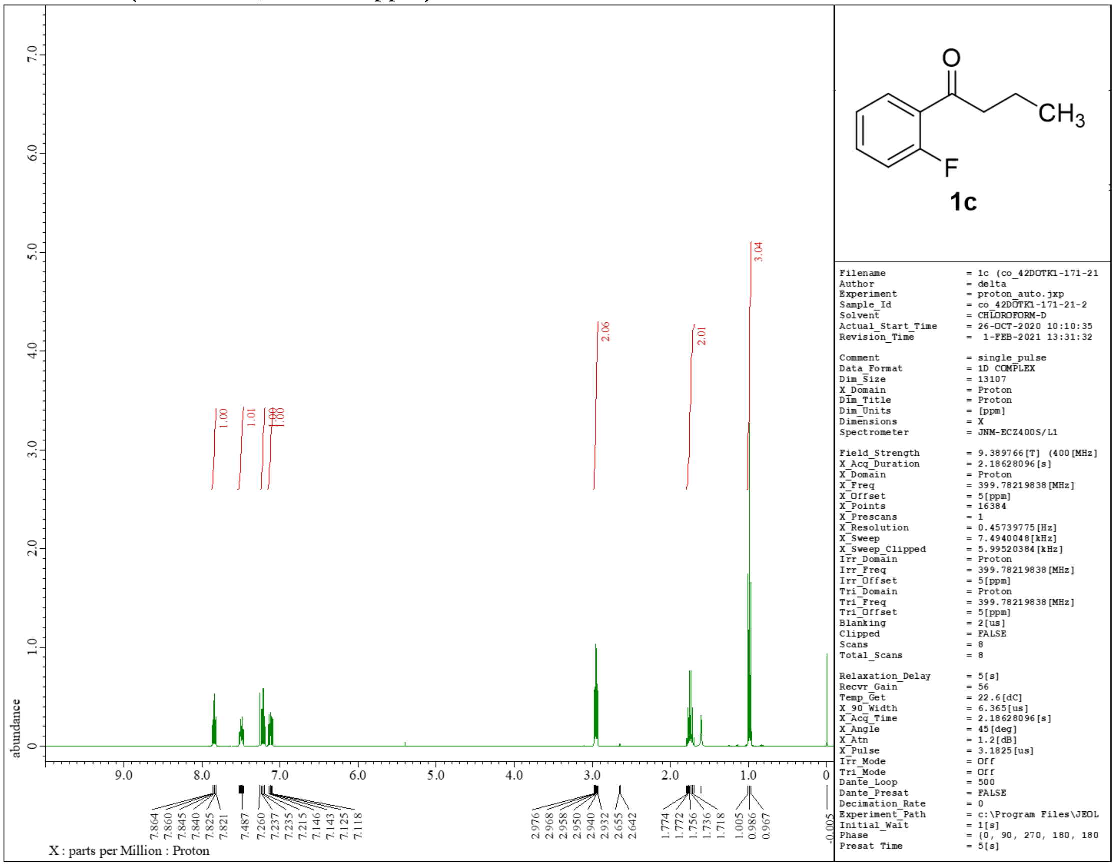

$^{13}C\{^{1}H\}$ NMR (100 MHz, $CDCl_3$, ppm)

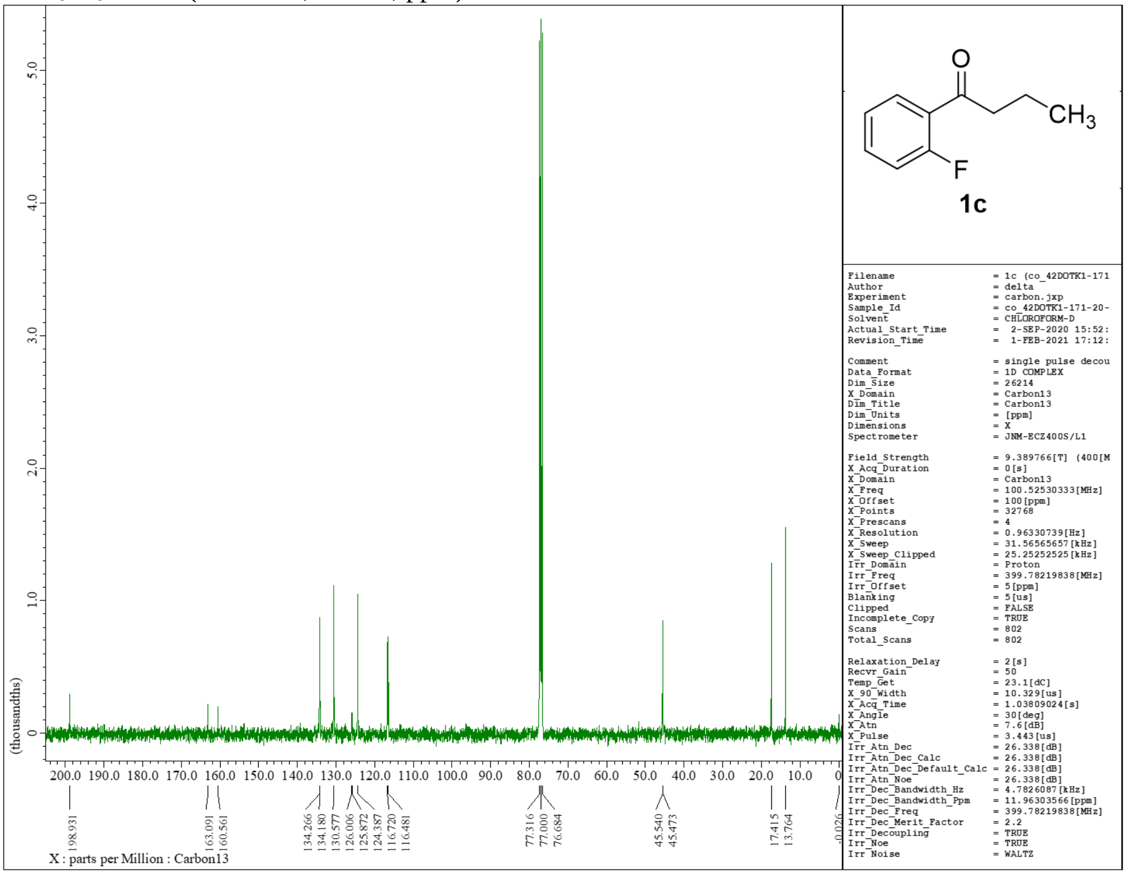

^{1}H NMR (400 MHz, $CDCl_3$, ppm)

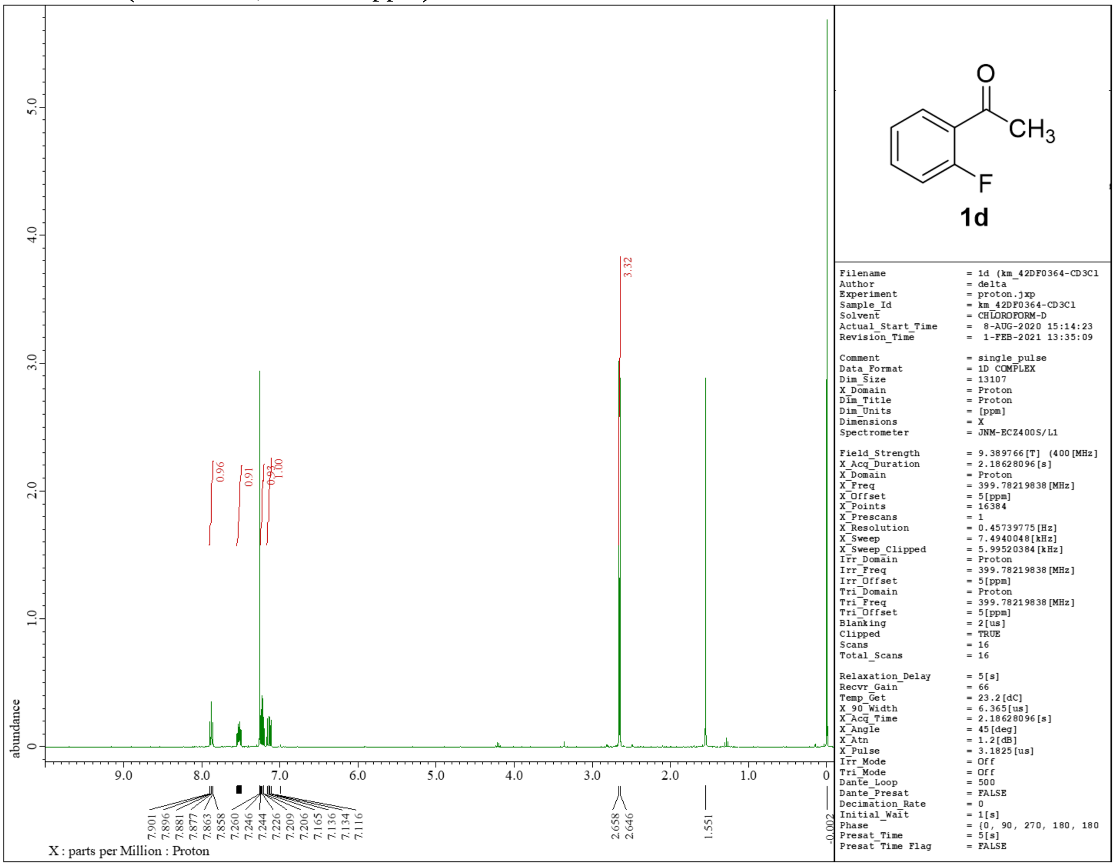

$^{13}C\{^{1}H\}$ NMR (100 MHz, $CDCl_3$, ppm)

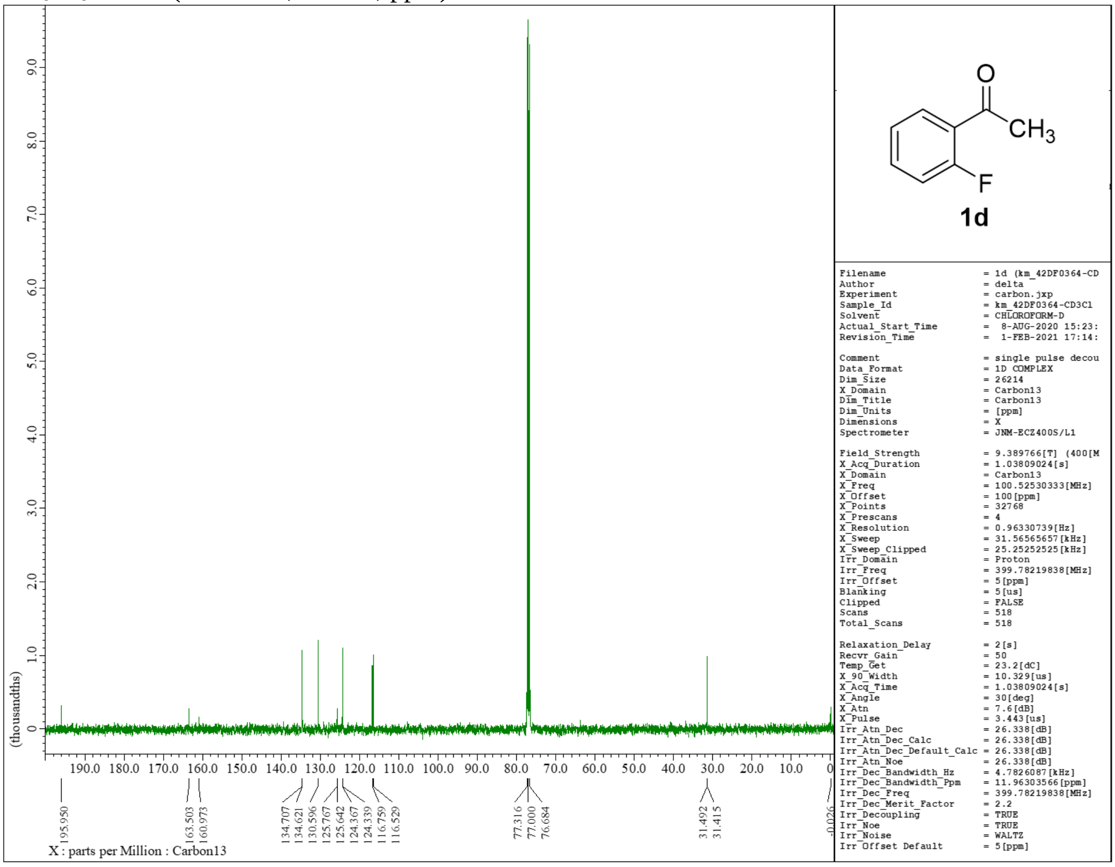

^{1}H NMR (400 MHz, $CDCl_3$, ppm)

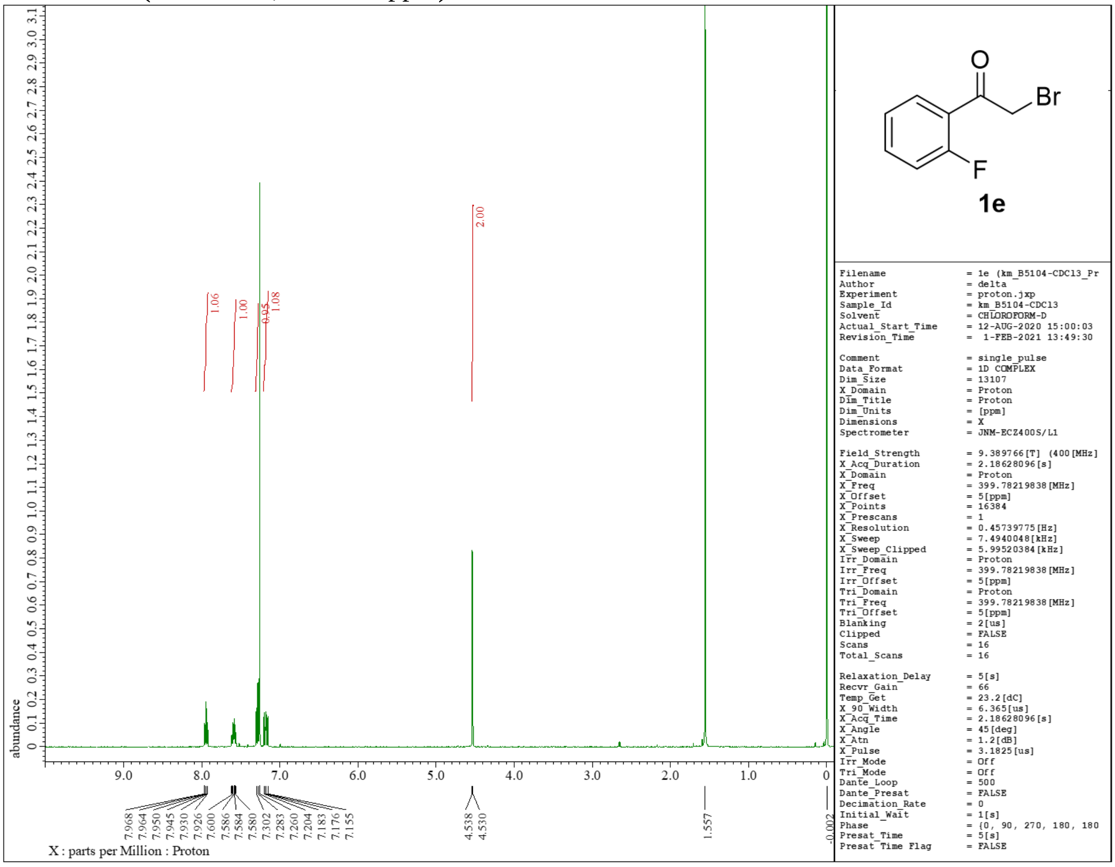

$^{13}C\{^{1}H\}$ NMR (100 MHz, $CDCl_3$, ppm)

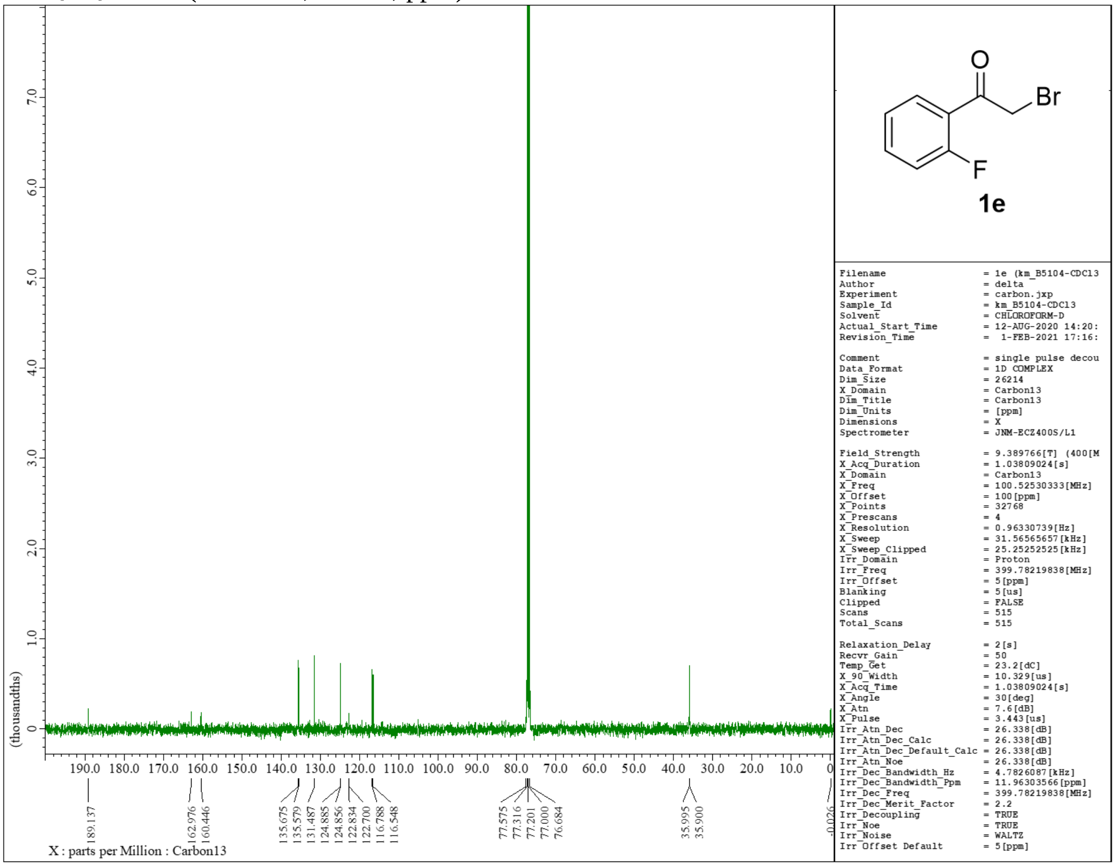

^{1}H NMR (400 MHz, $CDCl_3$, ppm)

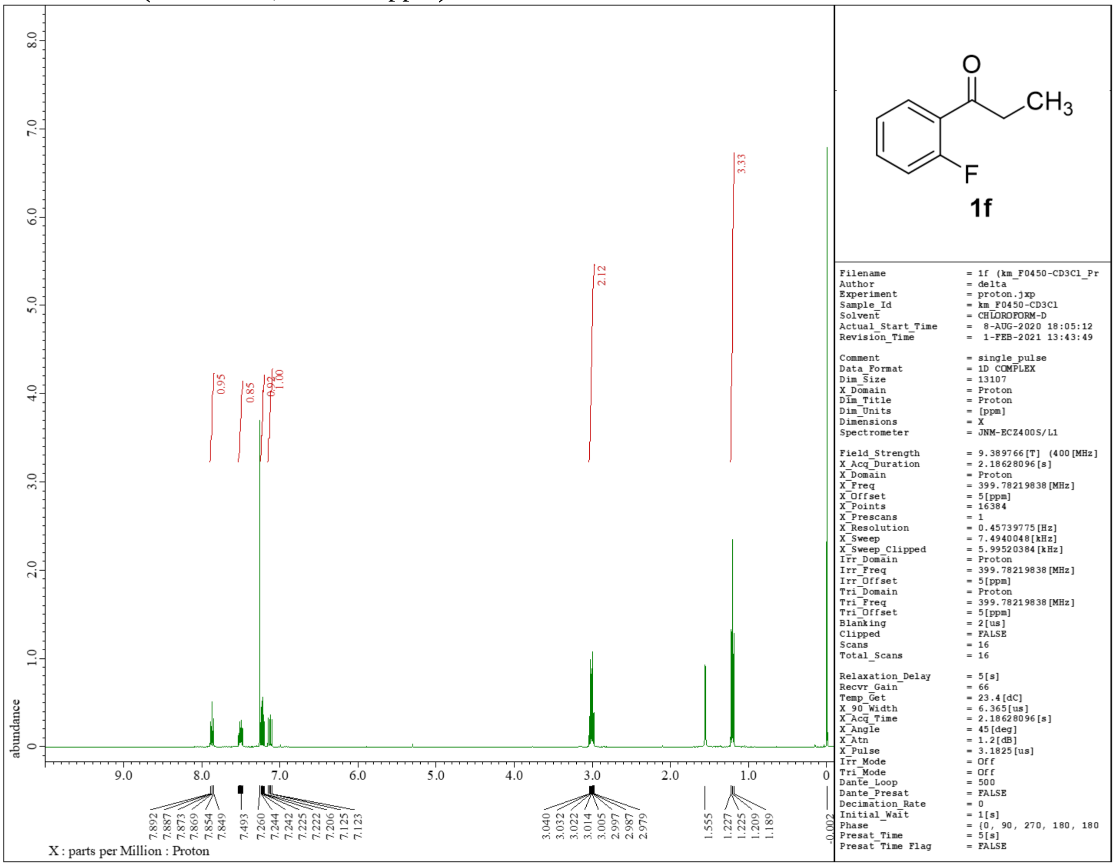

$^{13}C\{^{1}H\}$ NMR (100 MHz, $CDCl_3$, ppm)

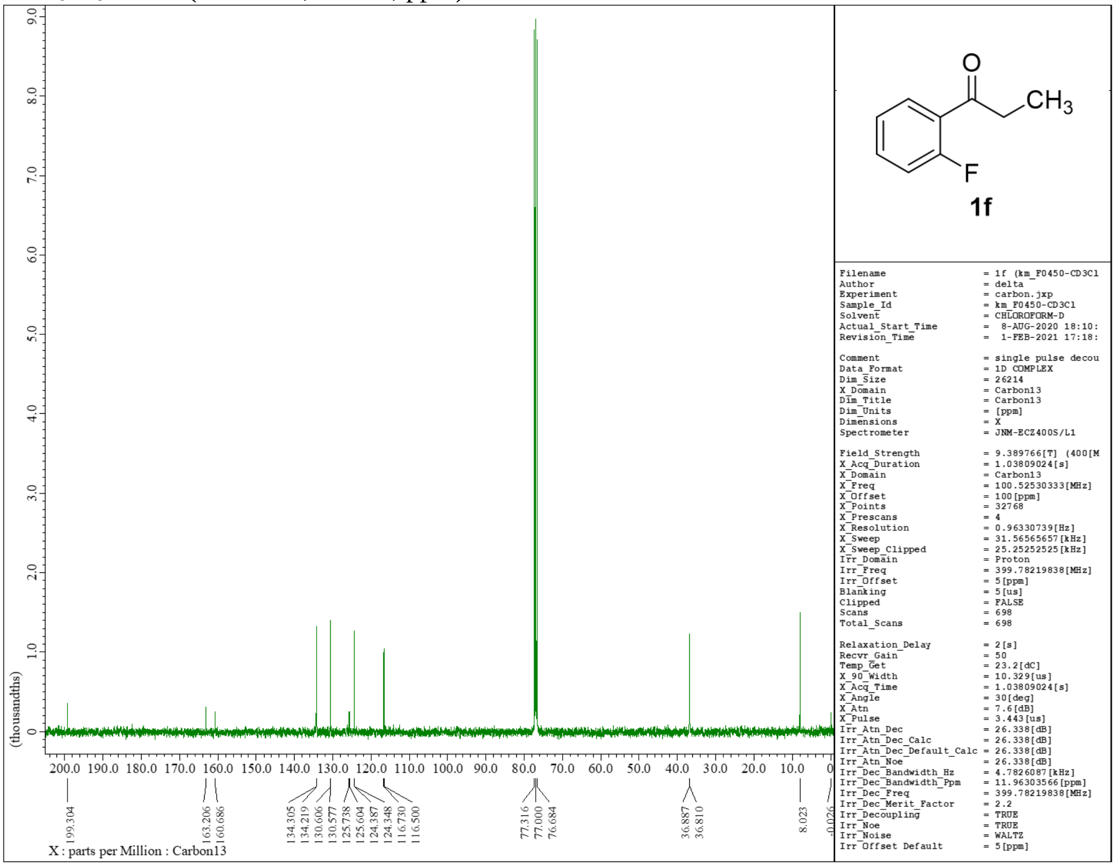

^{1}H NMR (400 MHz, $CDCl_3$, ppm)

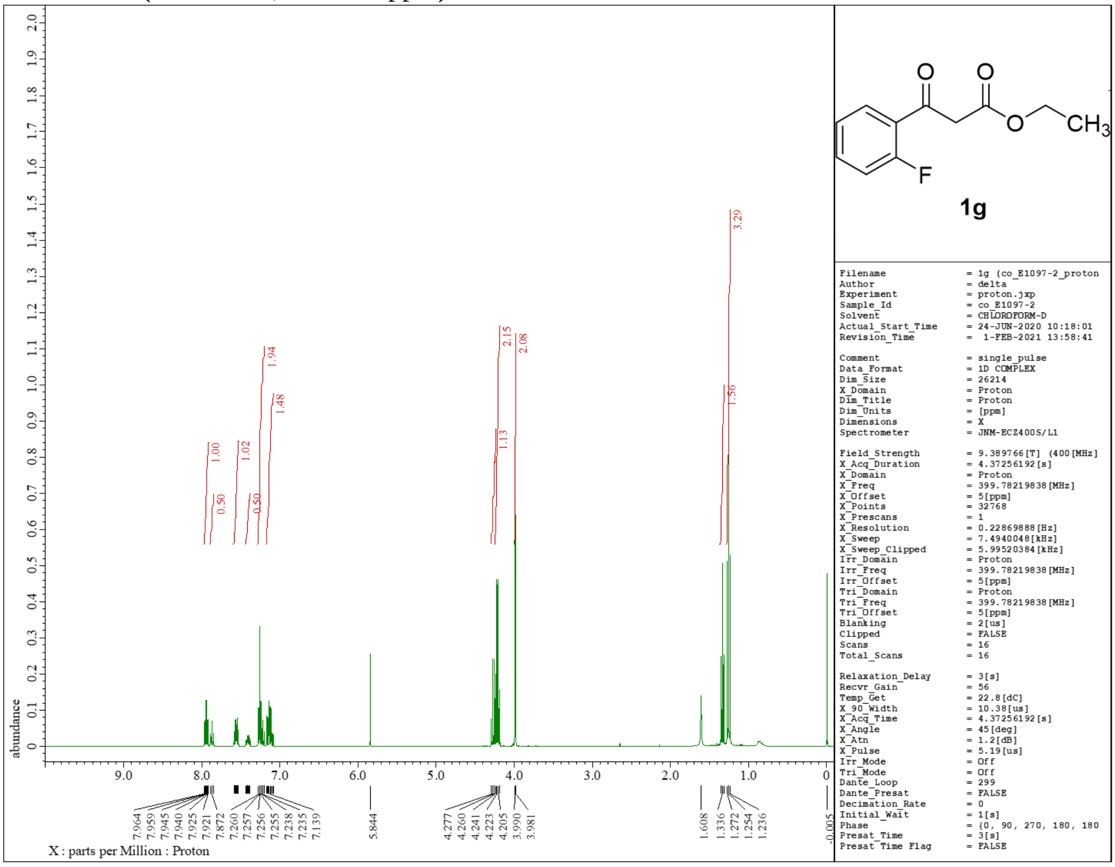

$^{13}C\{^{1}H\}$ NMR (100 MHz, $CDCl_3$, ppm)

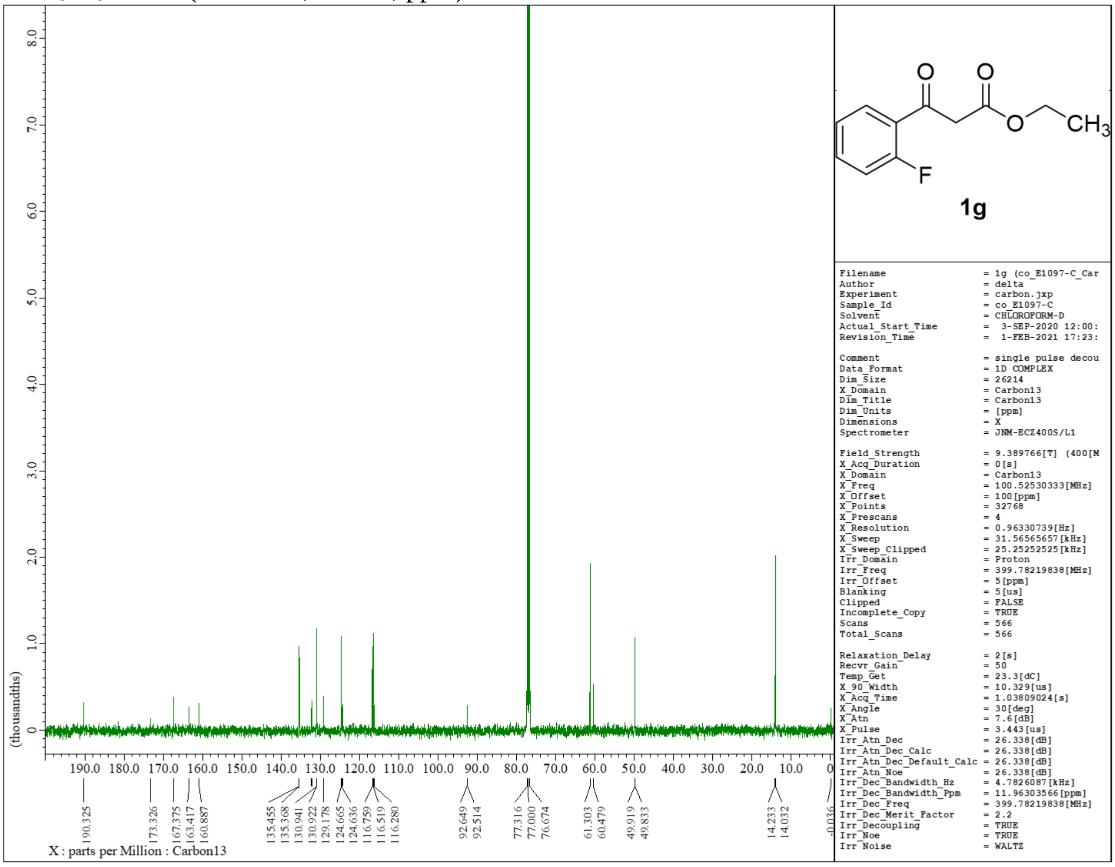

^{1}H NMR (400 MHz, $CDCl_3$, ppm)

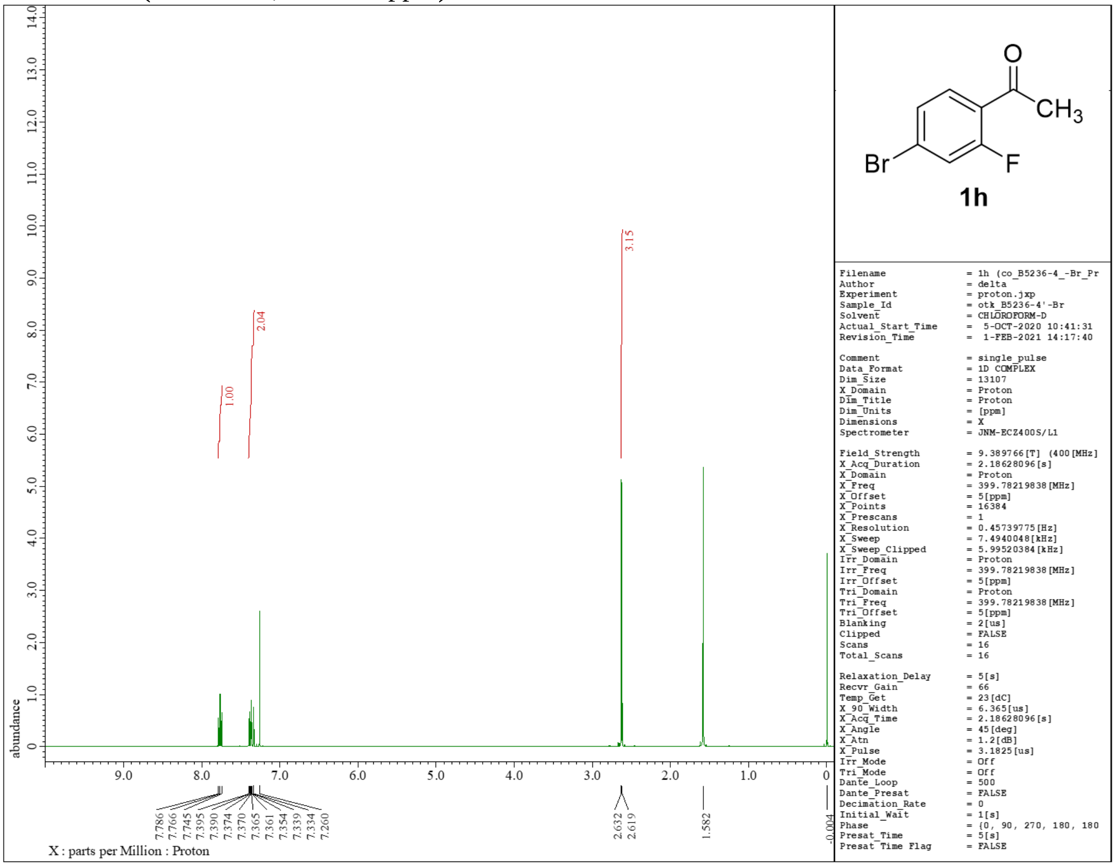

$^{13}C\{^1H\}$ NMR (100 MHz, $CDCl_3$, ppm)

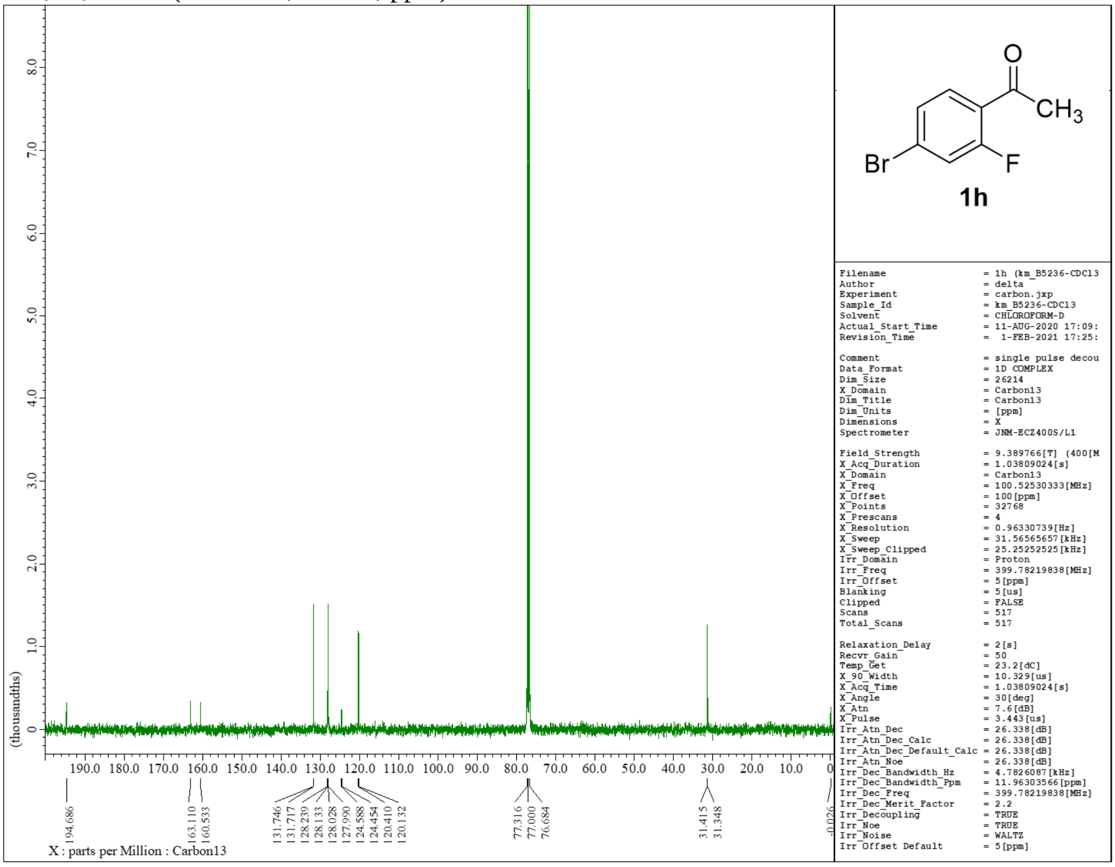

^{1}H NMR (400 MHz, $CDCl_3$, ppm)

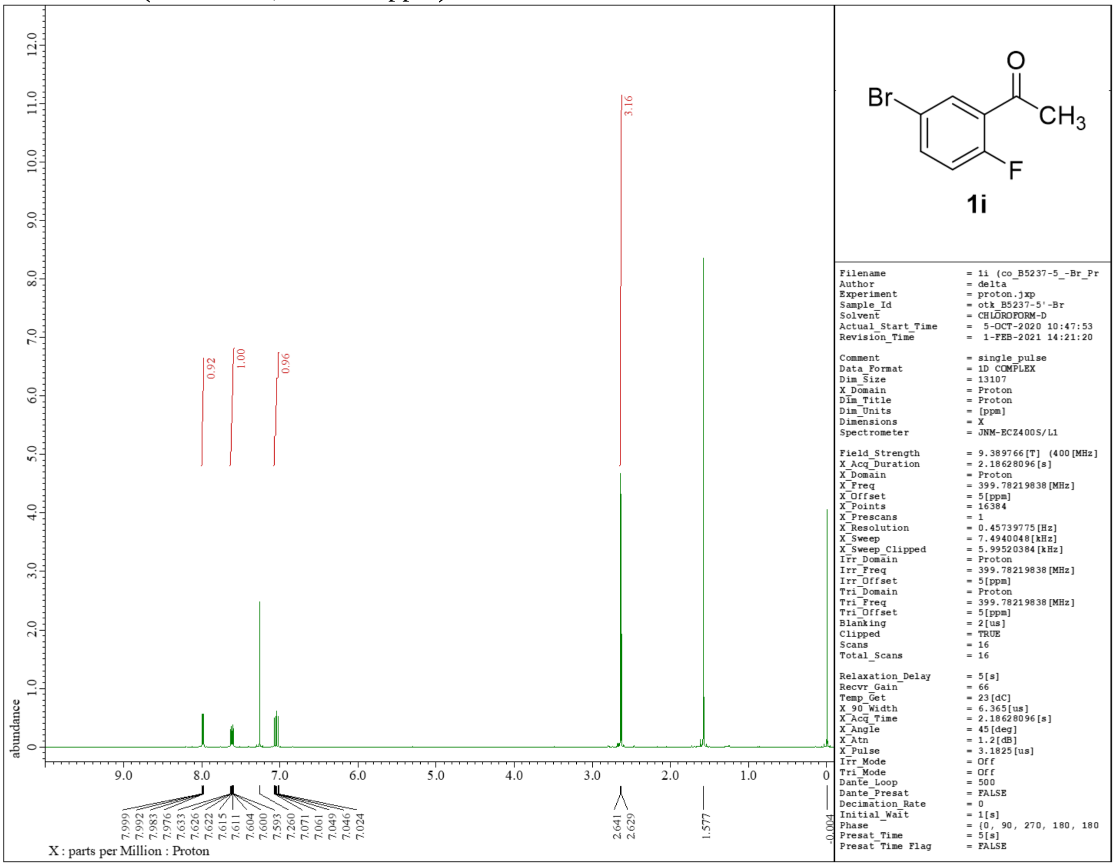

$^{13}C\{^{1}H\}$ NMR (100 MHz, $CDCl_3$, ppm)

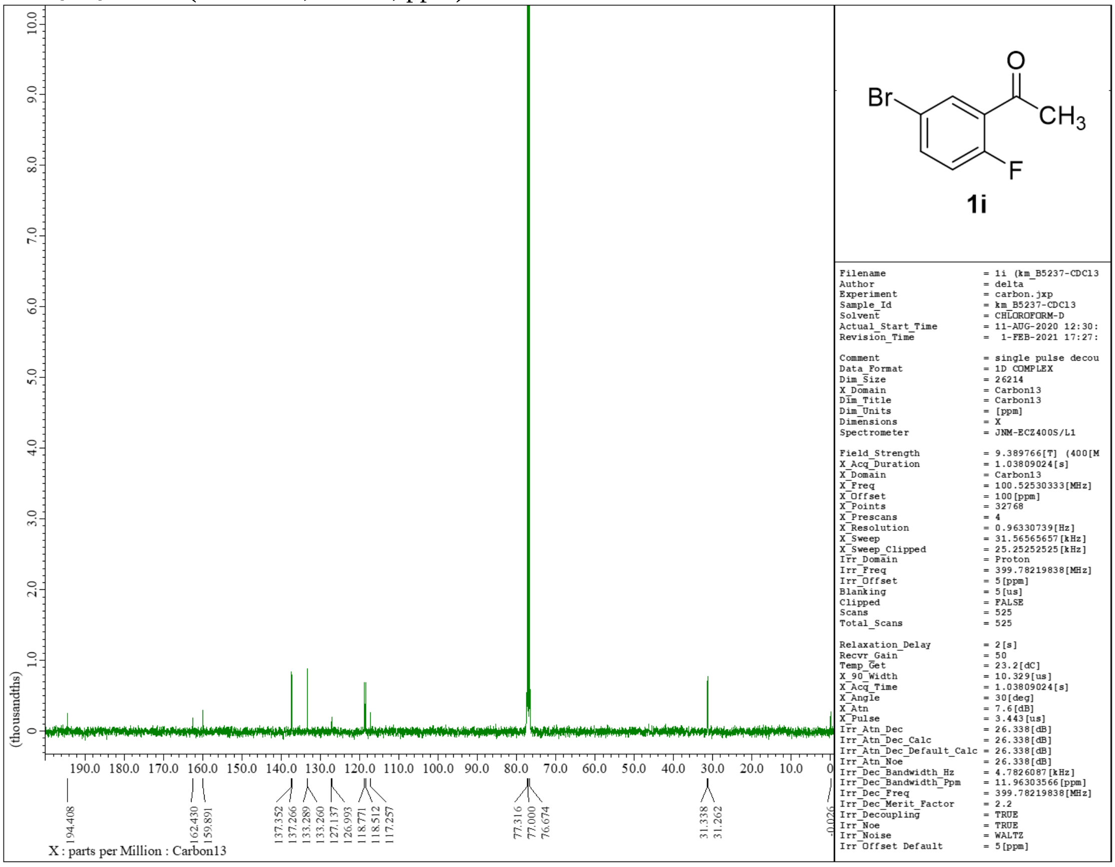

^{1}H NMR (400 MHz, $CDCl_3$, ppm)

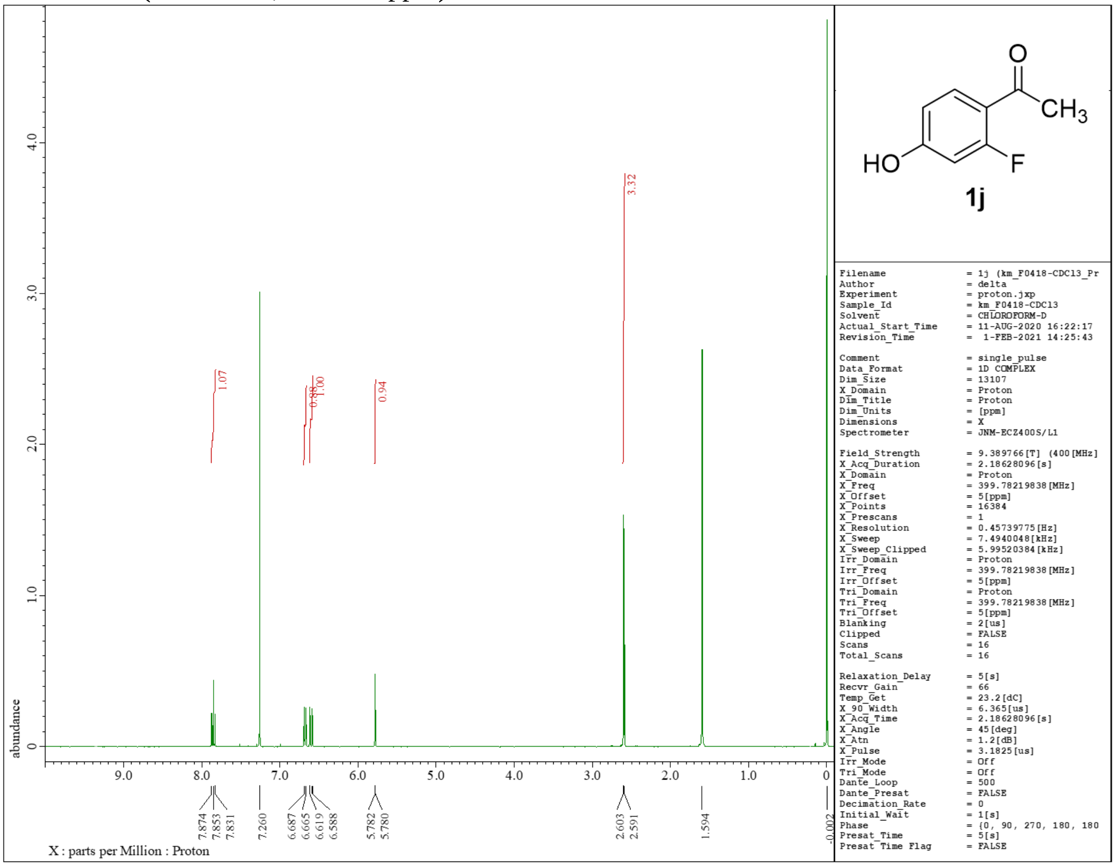

$^{13}C\{^{1}H\}$ NMR (100 MHz, $CDCl_3$, ppm)

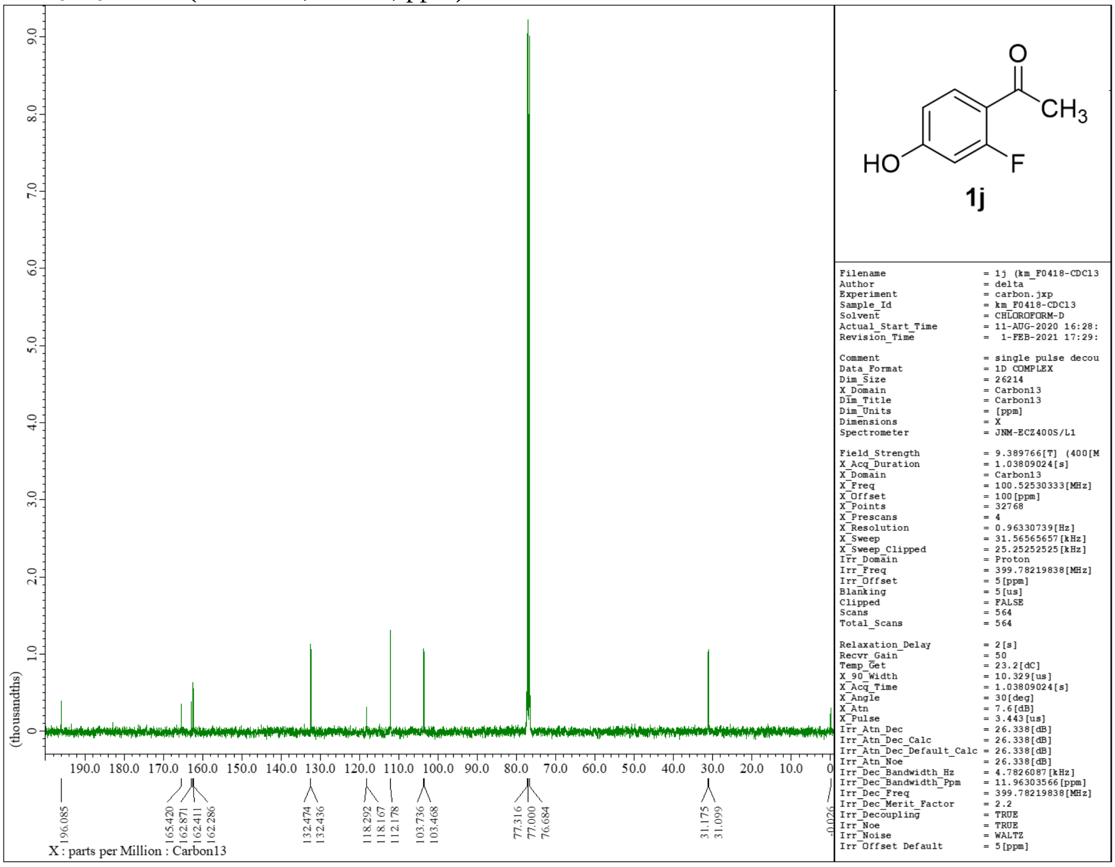

^{1}H NMR (400 MHz, $CDCl_3$, ppm)

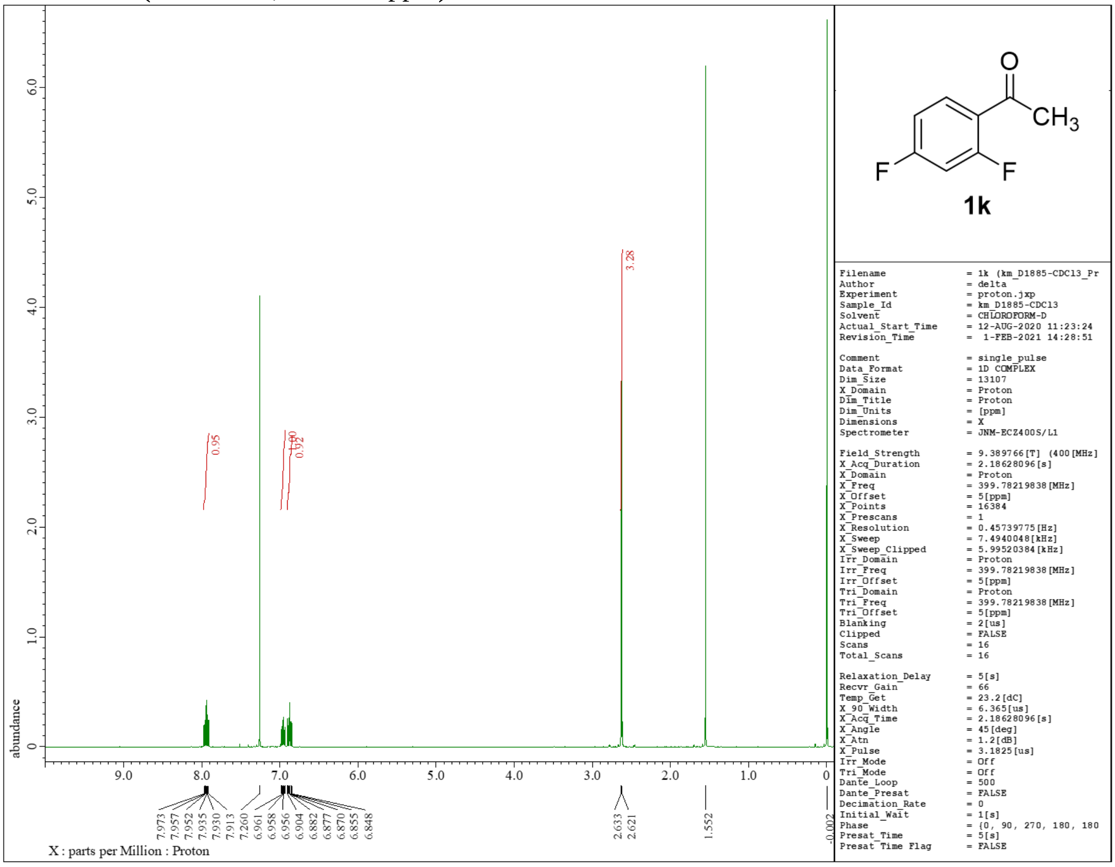

$^{13}C\{^{1}H\}$ NMR (100 MHz, $CDCl_3$, ppm)

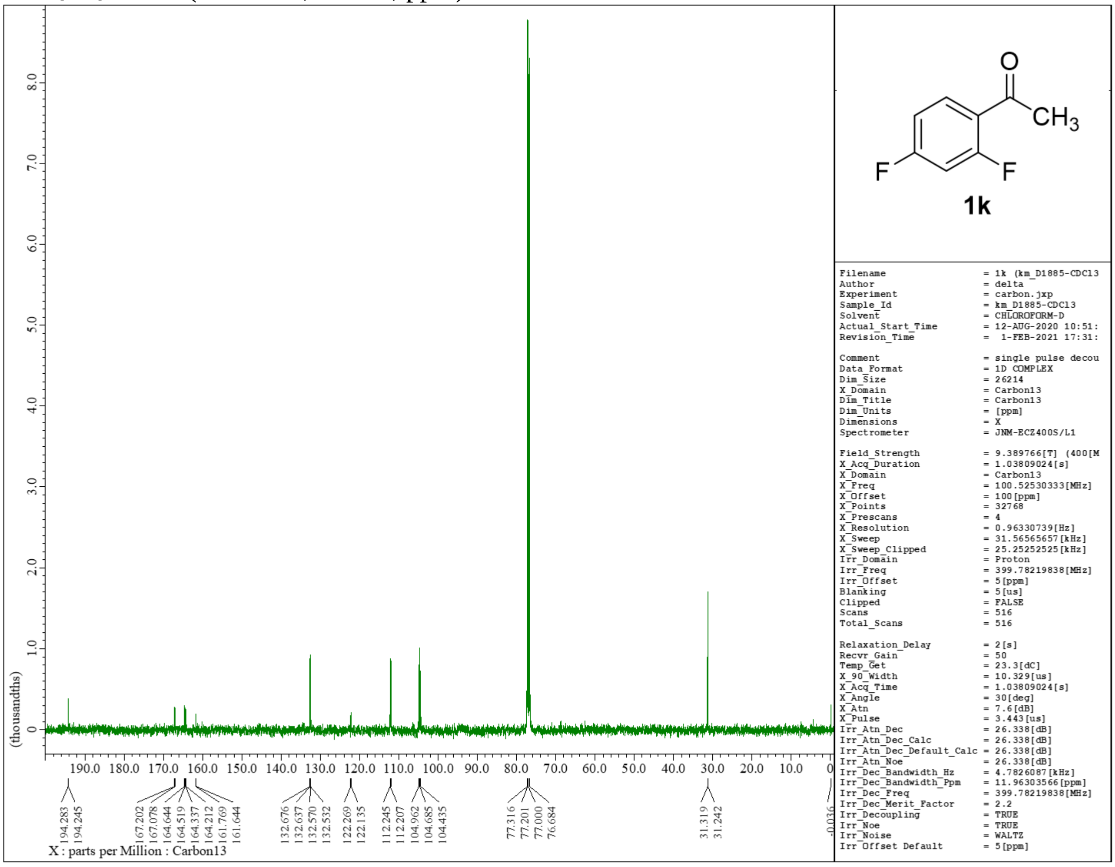

^{1}H NMR (400 MHz, $CDCl_3$, ppm)

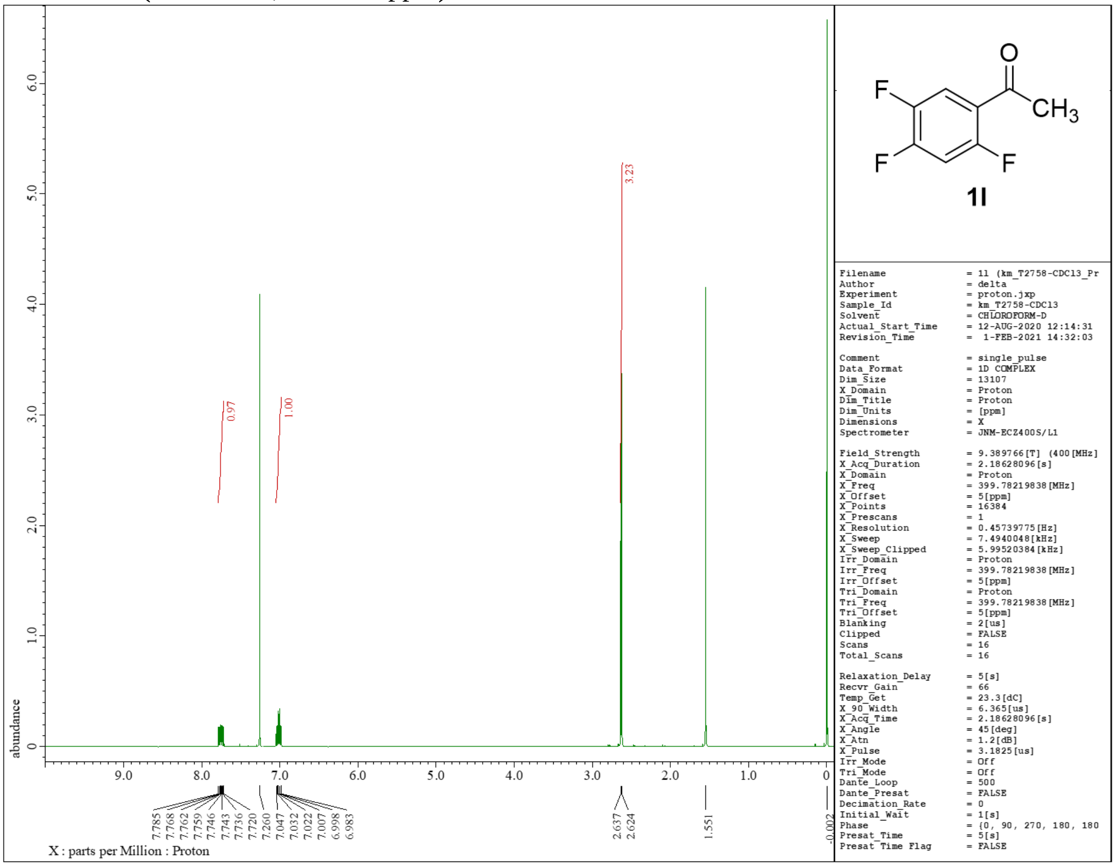

$^{13}C\{^{1}H\}$ NMR (100 MHz, $CDCl_3$, ppm)

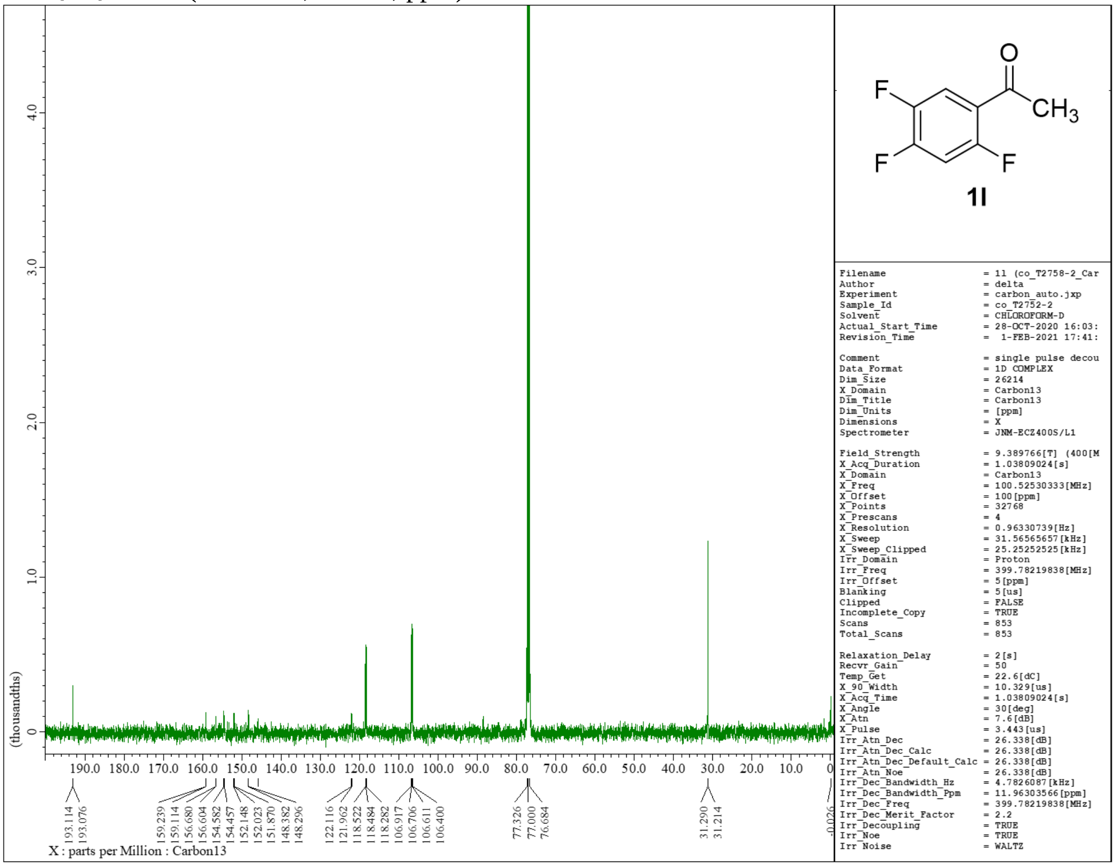

^{1}H NMR (400 MHz, $CDCl_3$, ppm)

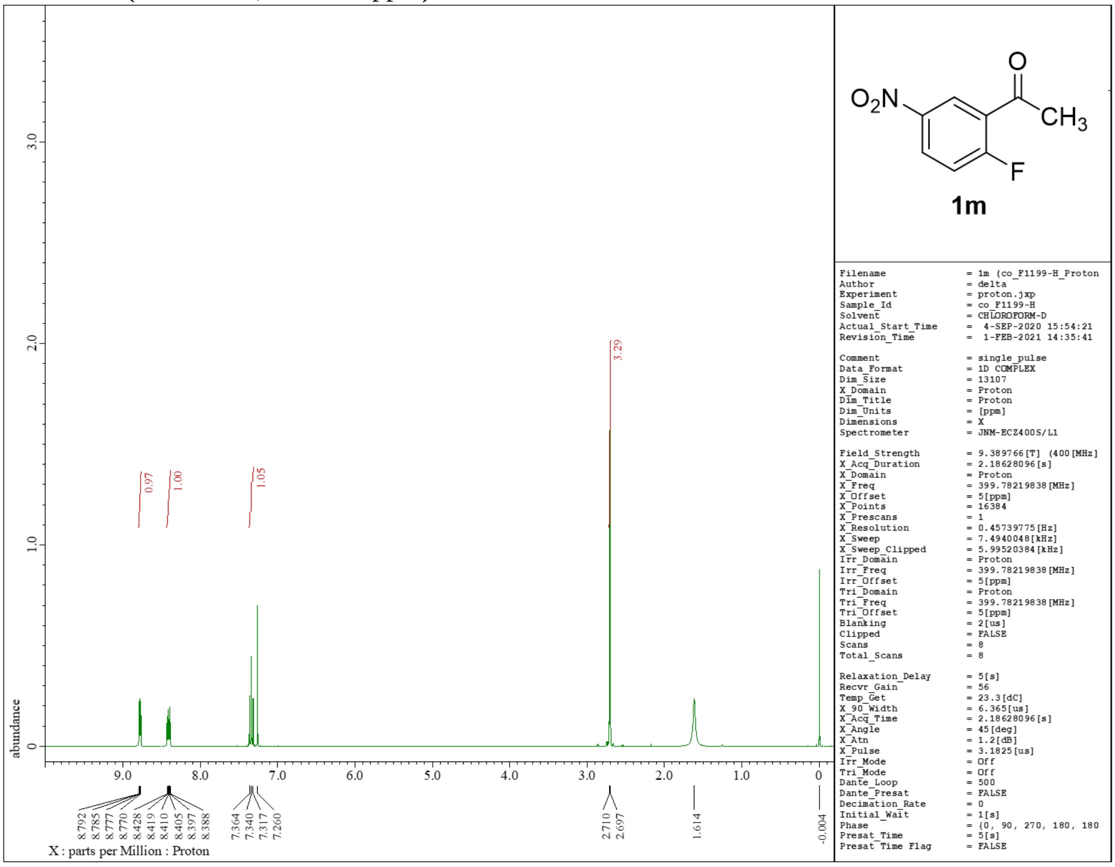

$^{13}C\{^{1}H\}$ NMR (100 MHz, $CDCl_3$, ppm)

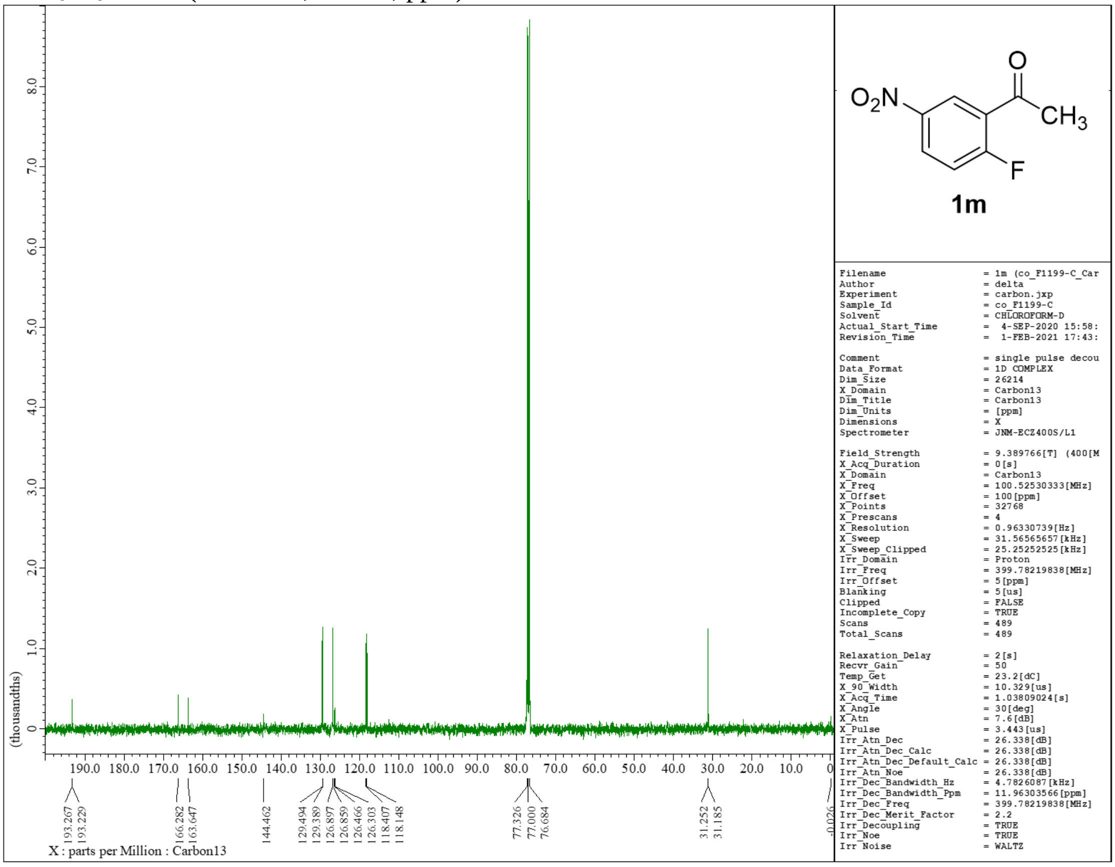

^{1}H NMR (400 MHz, $CDCl_3$, ppm)

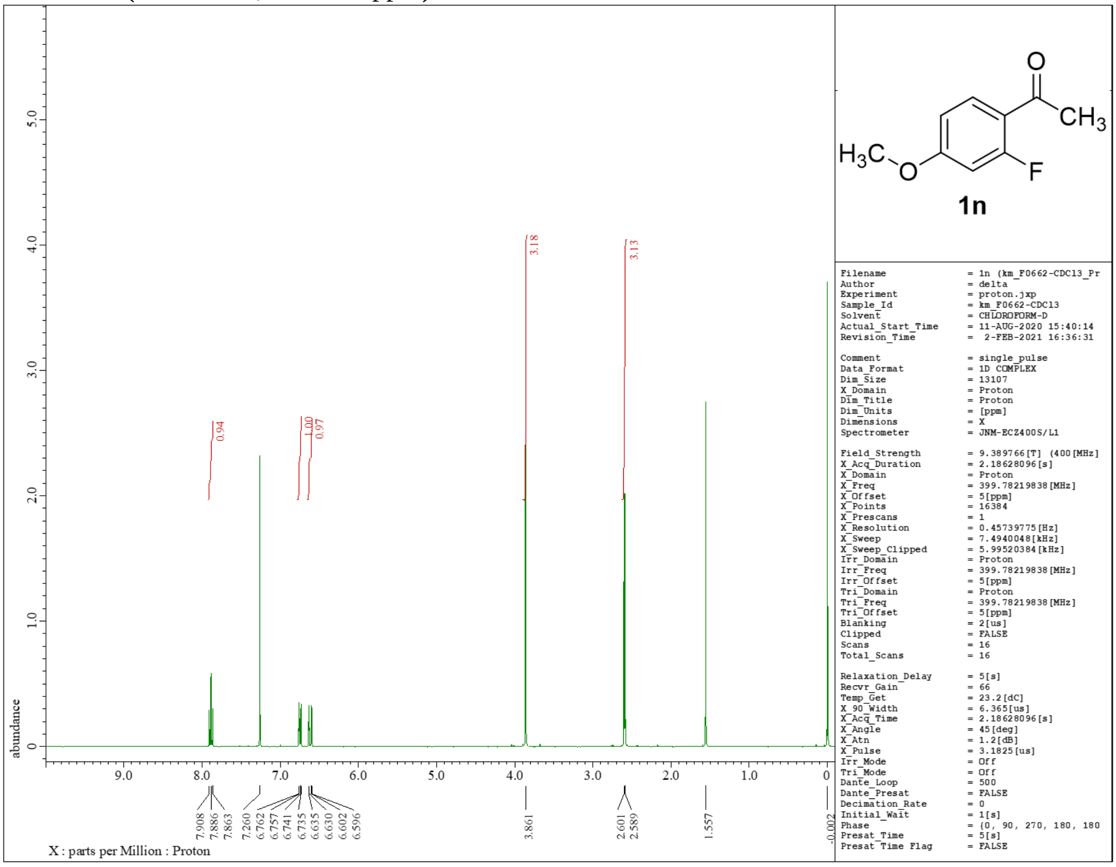

$^{13}C\{^{1}H\}$ NMR (100 MHz, $CDCl_3$, ppm)

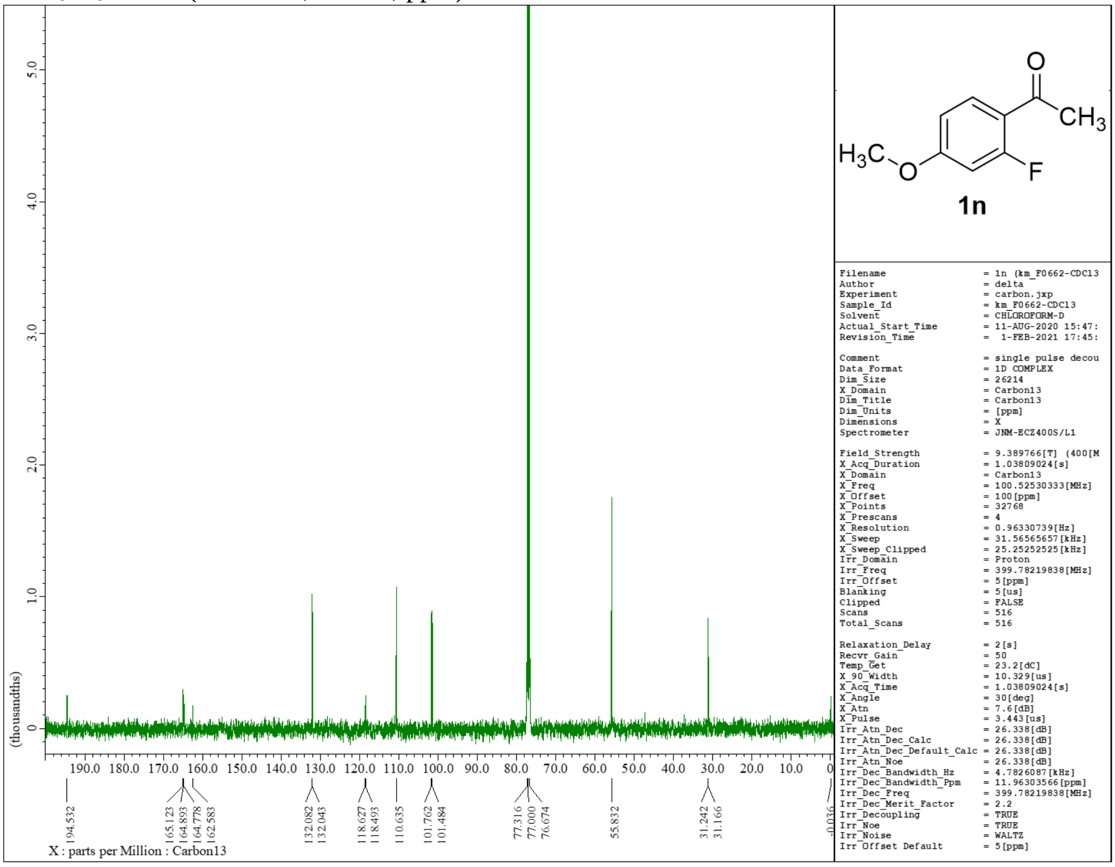

^1H NMR (400 MHz, $CDCl_3$, ppm)

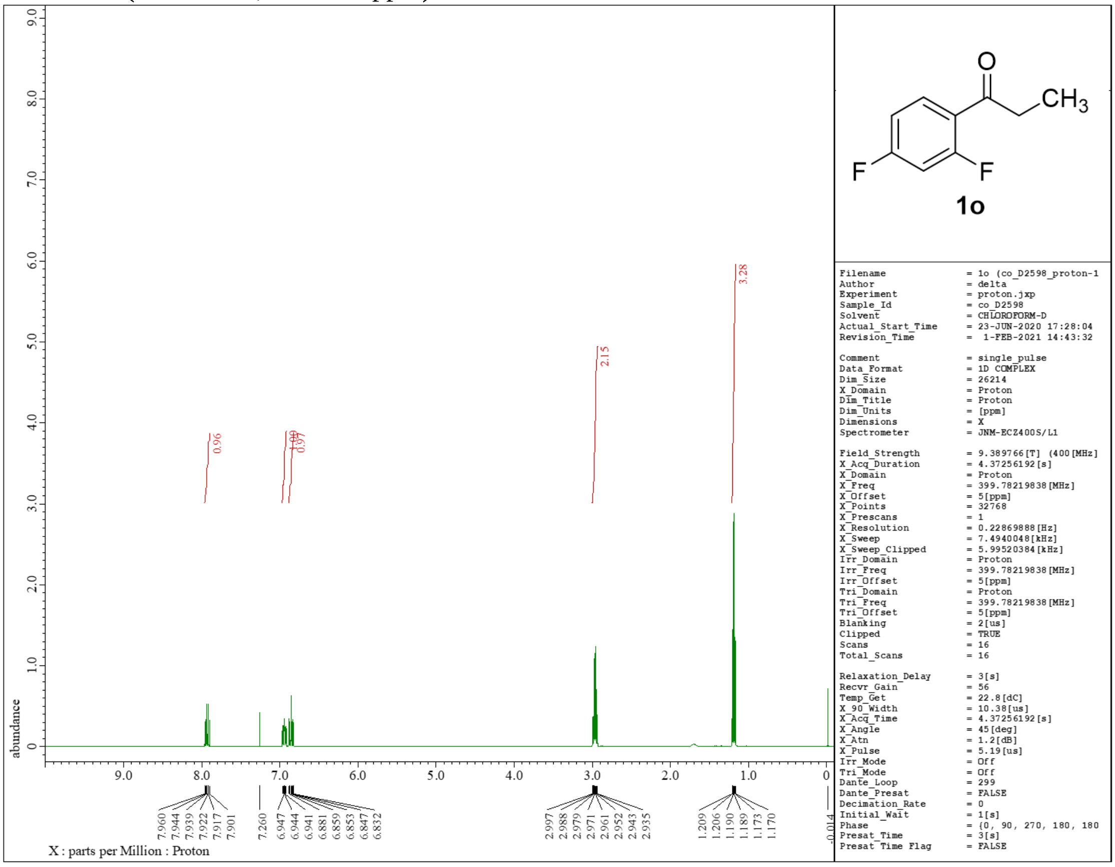

$^{13}C\{^1H\}$ NMR (100 MHz, $CDCl_3$, ppm)

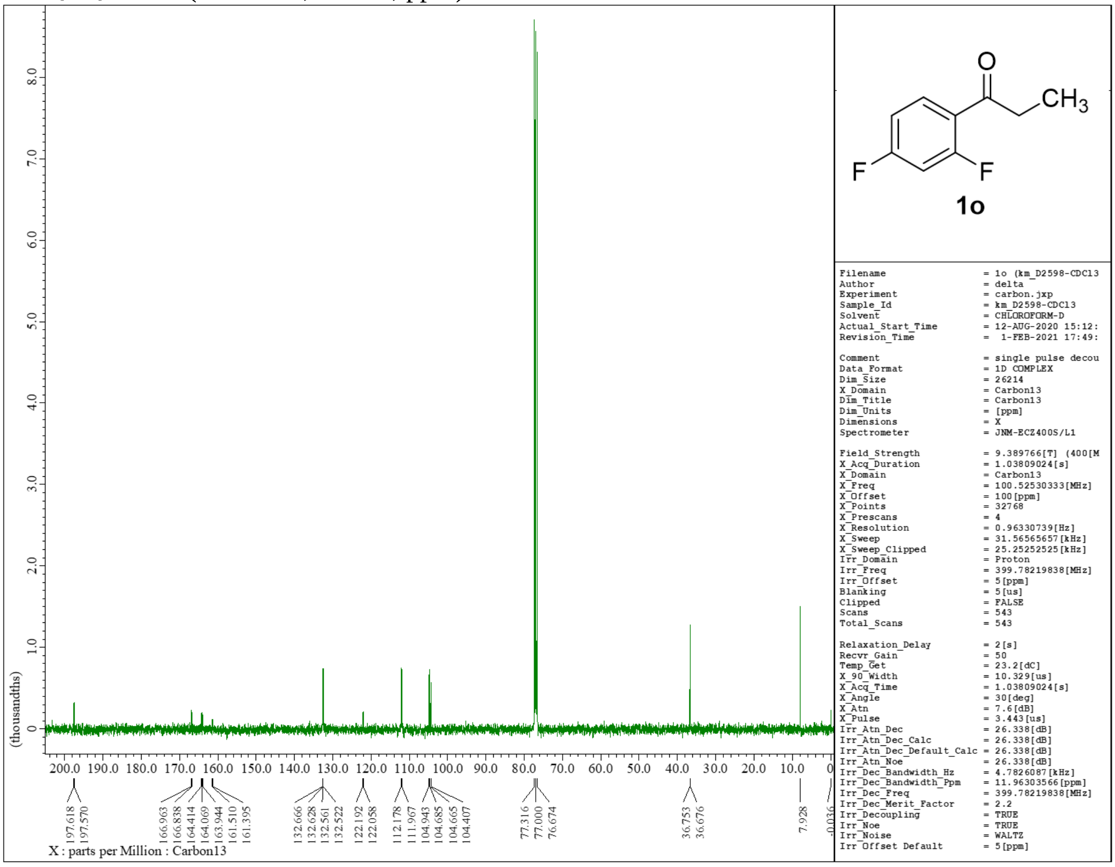

^1H NMR (400 MHz, $CDCl_3$, ppm)

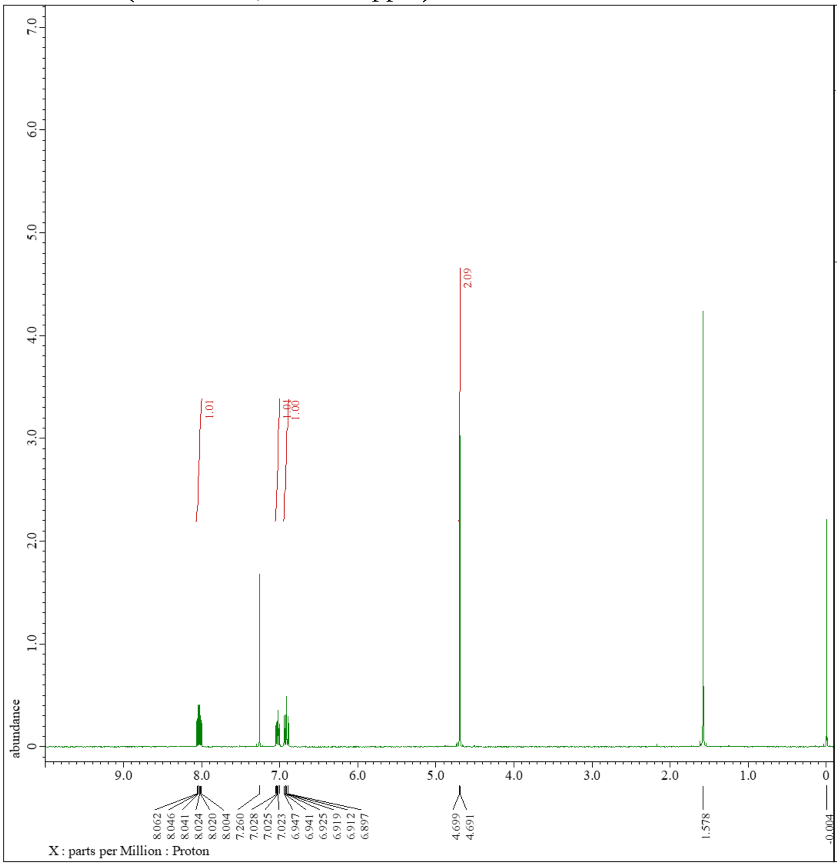

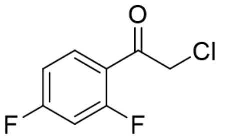

1p

```
Filename                = 1q (co_C2030_Proton-1
Author                  = delta
Experiment              = proton.jxp
Sample_Id               = co_C2030
Solvent                 = CHLOROFORM-D
Actual_Start_Time       =  7-SEP-2020 15:32:55
Revision_Time           =  1-FEB-2021 14:47:02

Comment                 = single_pulse
Data_Format             = 1D COMPLEX
Dim_Size                = 13107
X_Domain                = Proton
Dim_Title               = Proton
Dim_Units               = [ppm]
Dimensions              = X
Spectrometer            = JNM-ECZ400S/L1

Field_Strength          = 9.389766[T] (400[MHz]
X_Acq_Duration          = 2.18628096[s]
X_Domain                = Proton
X_Freq                  = 399.78219838[MHz]
X_Offset                = 5[ppm]
X_Points                = 16384
X_Prescans              = 1
X_Resolution            = 0.45739775[Hz]
X_Sweep                 = 7.4940048[kHz]
X_Sweep_Clipped         = 5.99520384[kHz]
Irr_Domain              = Proton
Irr_Freq                = 399.78219838[MHz]
Irr_Offset              = 5[ppm]
Tri_Domain              = Proton
Tri_Freq                = 399.78219838[MHz]
Tri_Offset              = 5[ppm]
Blanking                = 2[us]
Clipped                 = FALSE
Scans                   = 16
Total_Scans             = 16

Relaxation_Delay        = 5[s]
Recvr_Gain              = 66
Temp_Get                = 23.4[dC]
X_90_Width              = 6.365[us]
X_Acq_Time              = 2.18628096[s]
X_Angle                 = 45[deg]
X_Atn                   = 1.2[dB]
X_Pulse                 = 3.1825[us]
Irr_Mode                = Off
Tri_Mode                = Off
Dante_Loop              = 500
Dante_Presat            = FALSE
Decimation_Rate         = 0
Initial_Wait            = 1[s]
Phase                   = {0, 90, 270, 180, 180
Presat_Time             = 5[s]
Presat_Time_Flag        = FALSE
```

$^{13}C\{^1H\}$ NMR (100 MHz, $CDCl_3$, ppm)

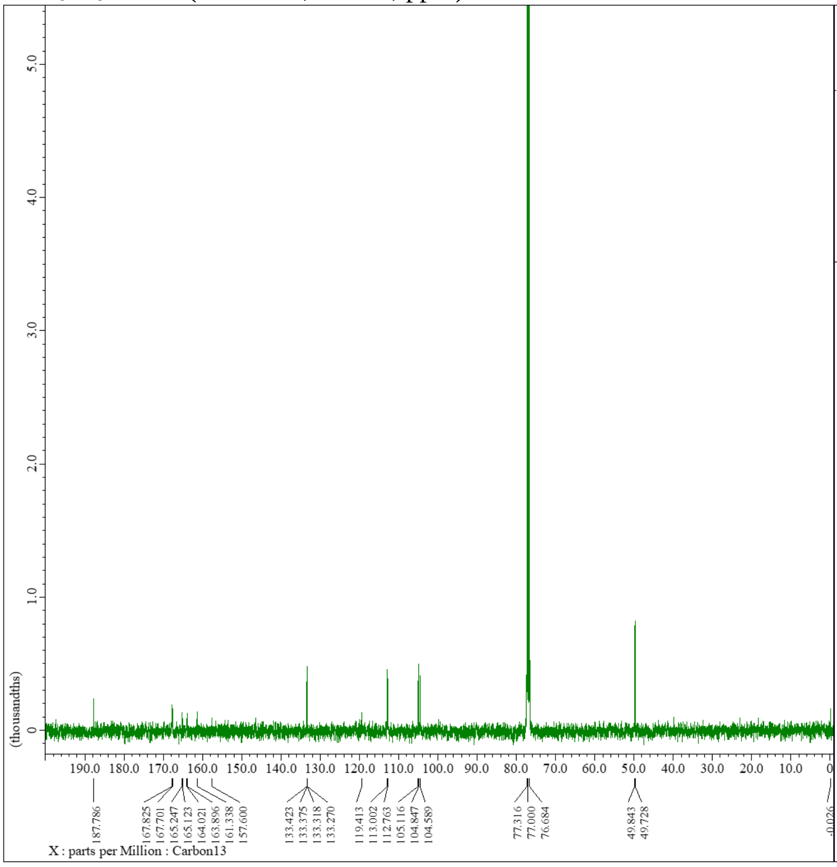

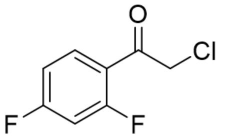

1p

```
Filename                    = 1q (co_C2030 1_Car
Author                      = delta
Experiment                  = carbon.jxp
Sample_Id                   = co_C2030 1
Solvent                     = CHLOROFORM-D
Actual_Start_Time           =  7-SEP-2020 15:39:
Revision_Time               =  1-FEB-2021 17:53:

Comment                     = single pulse decou
Data_Format                 = 1D COMPLEX
Dim_Size                    = 26214
X_Domain                    = Carbon13
Dim_Title                   = Carbon13
Dim_Units                   = [ppm]
Dimensions                  = X
Spectrometer                = JNM-ECZ400S/L1

Field_Strength              = 9.389766[T] (400[M
X_Acq_Duration              = 0[s]
X_Domain                    = Carbon13
X_Freq                      = 100.52530333[MHz]
X_Offset                    = 100[ppm]
X_Points                    = 32768
X_Prescans                  = 4
X_Resolution                = 0.96330739[Hz]
X_Sweep                     = 31.56565657[kHz]
X_Sweep_Clipped             = 25.25252525[kHz]
Irr_Domain                  = Proton
Irr_Freq                    = 399.78219838[MHz]
Irr_Offset                  = 5[ppm]
Blanking                    = 5[us]
Clipped                     = FALSE
Incomplete_Copy             = TRUE
Scans                       = 620
Total_Scans                 = 620

Relaxation_Delay            = 2[s]
Recvr_Gain                  = 50
Temp_Get                    = 23.2[dC]
X_90_Width                  = 10.329[us]
X_Acq_Time                  = 1.03809024[s]
X_Angle                     = 30[deg]
X_Atn                       = 7.6[dB]
X_Pulse                     = 3.443[us]
Irr_Atn_Dec                 = 26.338[dB]
Irr_Atn_Dec_Calc            = 26.338[dB]
Irr_Atn_Dec_Default_Calc    = 26.338[dB]
Irr_Atn_Noe                 = 26.338[dB]
Irr_Dec_Bandwidth_Hz        = 4.7826087[kHz]
Irr_Dec_Bandwidth_Ppm       = 11.96303566[ppm]
Irr_Dec_Freq                = 399.78219838[MHz]
Irr_Dec_Merit_Factor        = 2.2
Irr_Decoupling              = TRUE
Irr_Noe                     = TRUE
Irr_Noise                   = WALTZ
```

^{1}H NMR (400 MHz, $CDCl_3$, ppm)

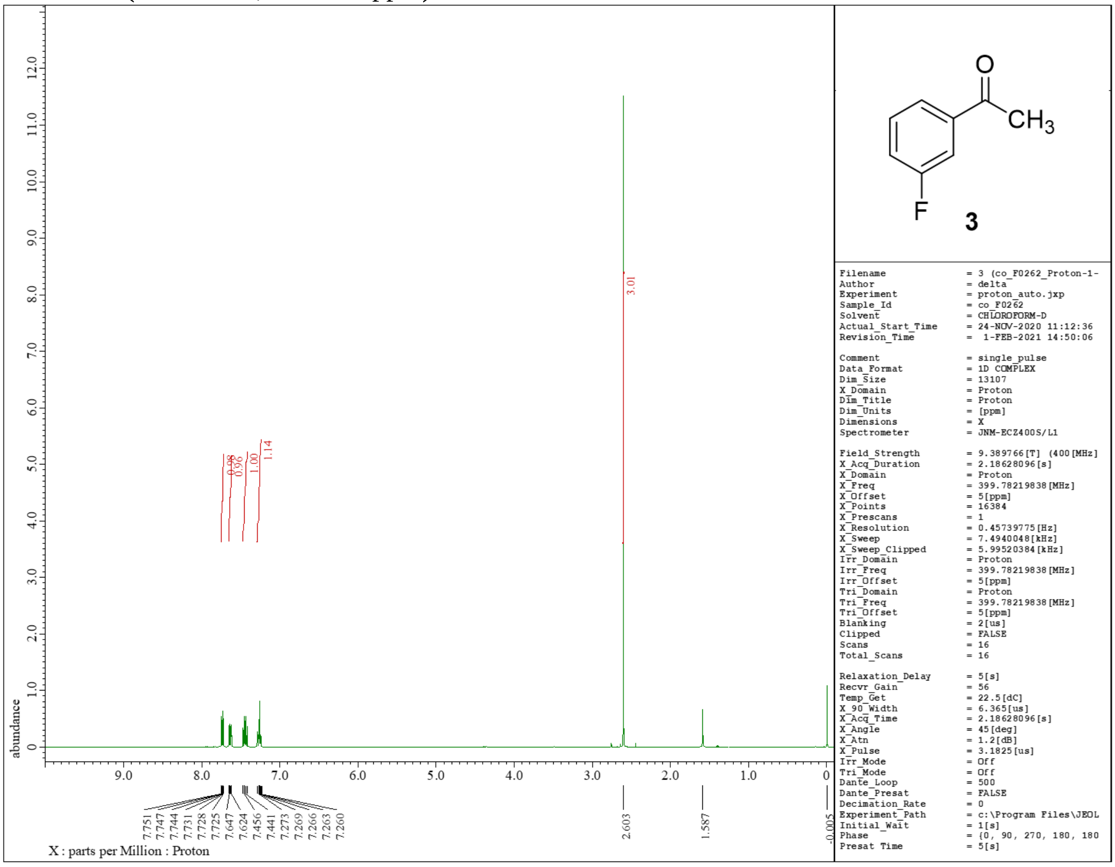

$^{13}C\{^{1}H\}$ NMR (100 MHz, $CDCl_3$, ppm)

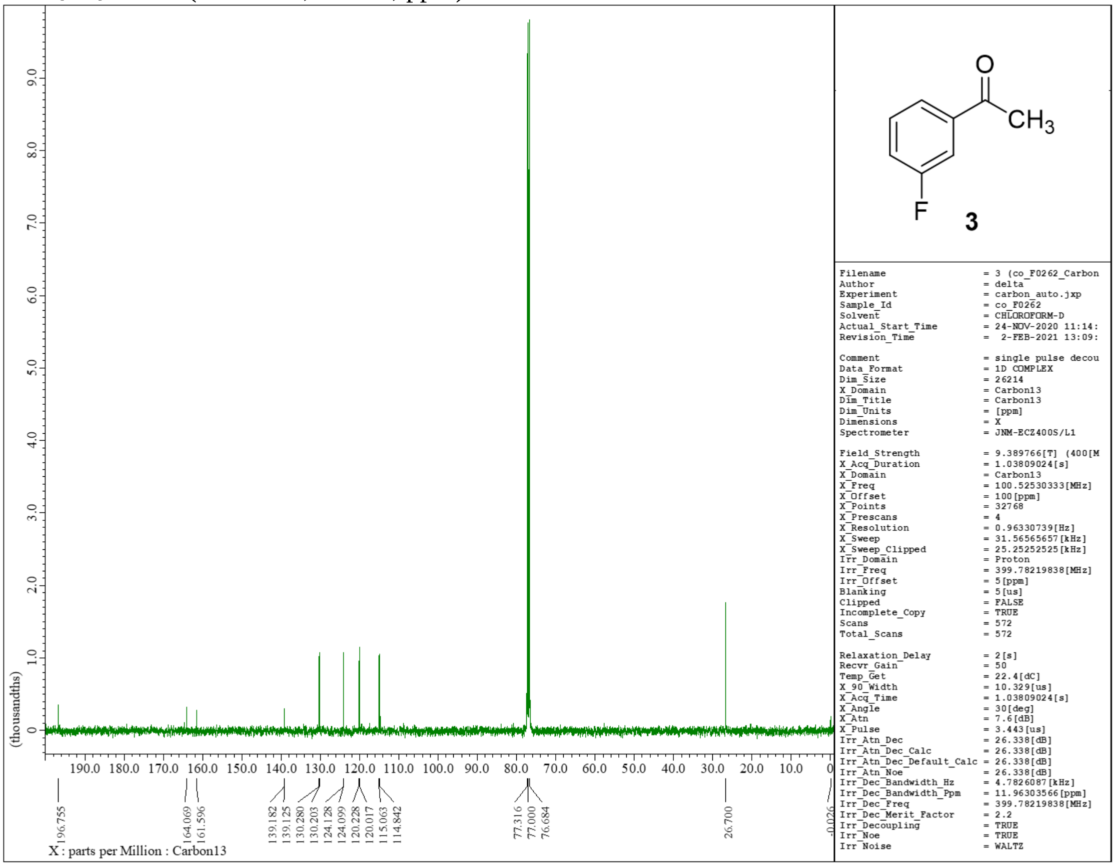

^1H NMR (400 MHz, $CDCl_3$, ppm)

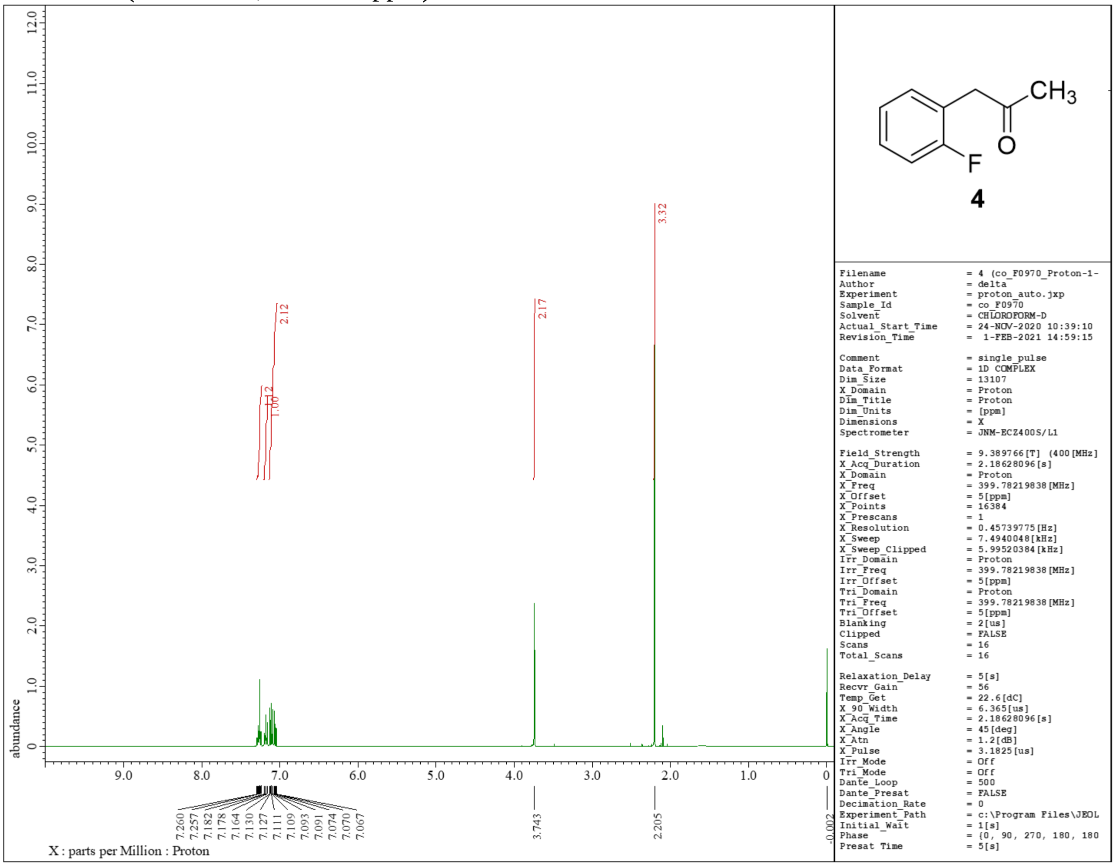

$^{13}C\{^1H\}$ NMR (100 MHz, $CDCl_3$, ppm)

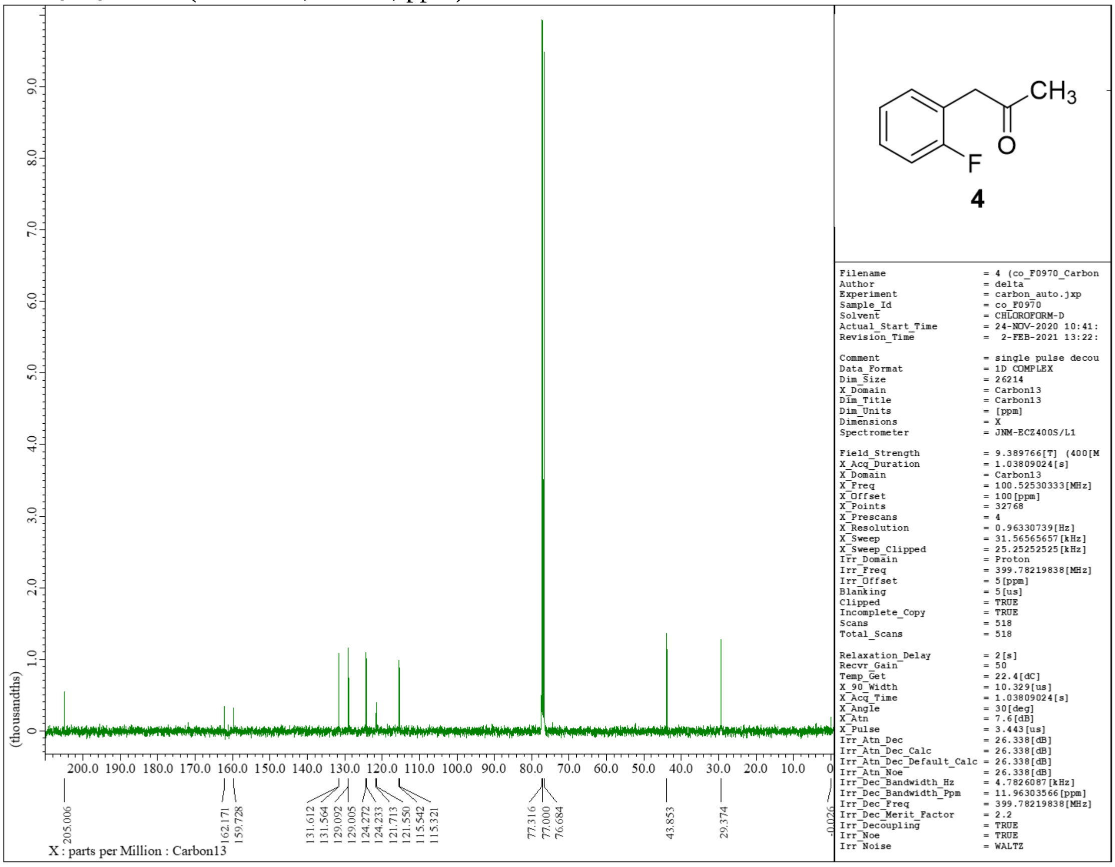

^{1}H NMR (400 MHz, $CDCl_3$, ppm)

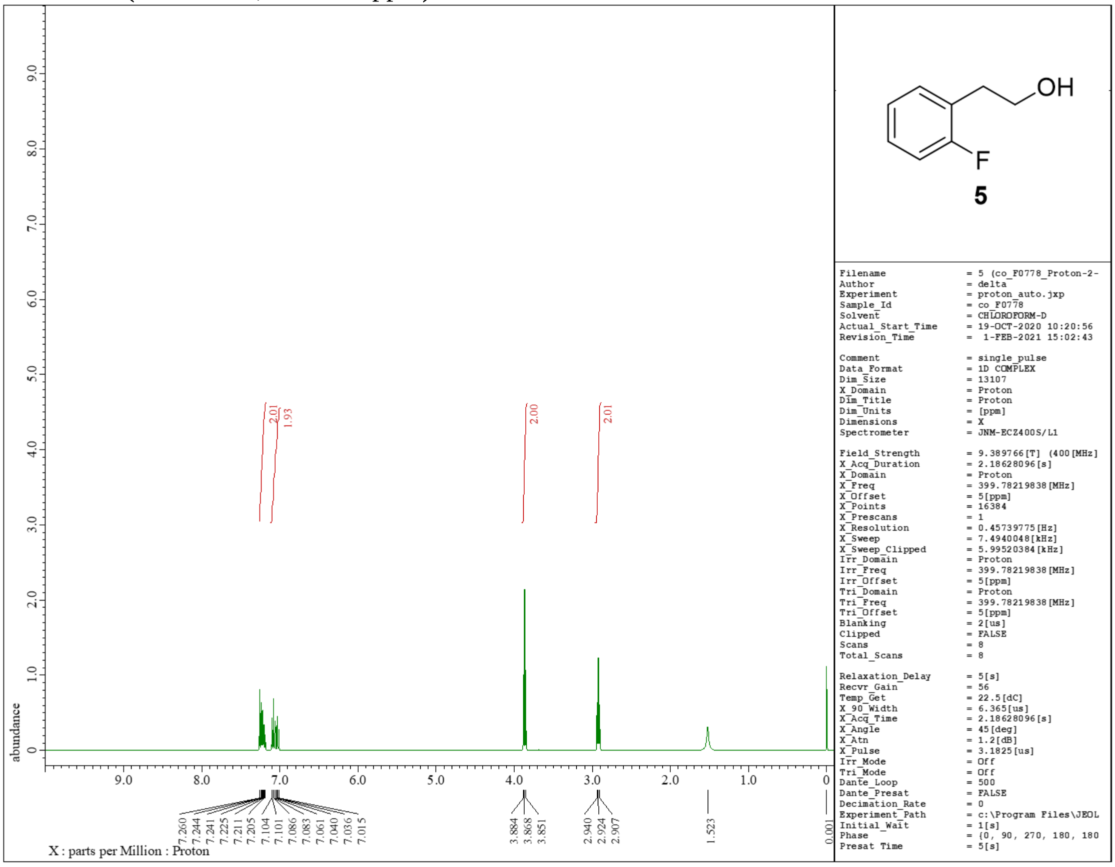

^{13}C{^{1}H} NMR (100 MHz, $CDCl_3$, ppm)

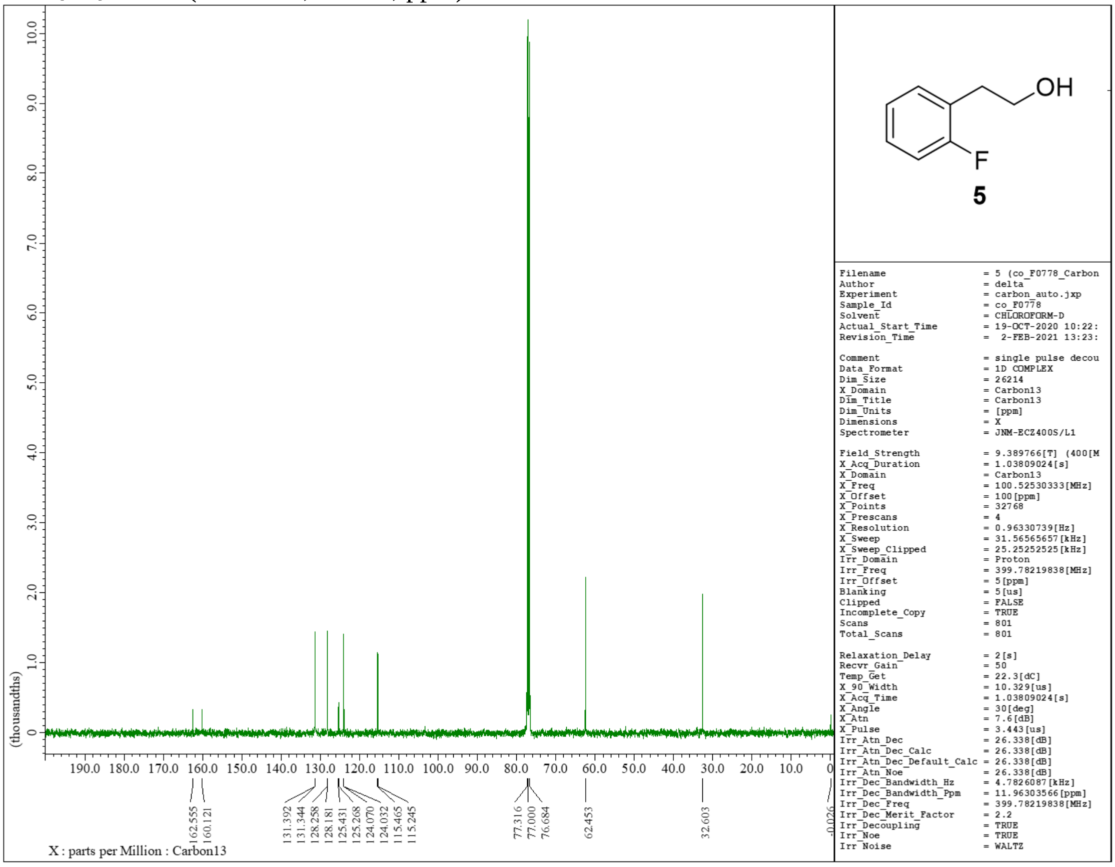

Supplement: Supplementary file 1 — jo1c00051_si_001.pdf [file jo1c00051_si_001.pdf]
